# Surveillance for Health Care Access and Health Services Use, Adults Aged 18**–**64 Years — Behavioral Risk Factor Surveillance System, United States, 2014

**DOI:** 10.15585/mmwr.ss6607a1

**Published:** 2017-02-24

**Authors:** Catherine A. Okoro, Guixiang Zhao, Jared B. Fox, Paul I. Eke, Kurt J. Greenlund, Machell Town

**Affiliations:** 1 Population Health Surveillance Branch, Division of Population Health, National Center for Chronic Disease Prevention and Health Promotion, CDC; 2 Policy Research, Analysis, and Development Office, Office of the Associate Director for Policy, CDC; 3 Division of Population Health, Office of the Director, National Center for Chronic Disease Prevention and Health Promotion, CDC

## Abstract

**Problem/Condition:**

As a result of the 2010 Patient Protection and Affordable Care Act, millions of U.S. adults attained health insurance coverage. However, millions of adults remain uninsured or underinsured. Compared with adults without barriers to health care, adults who lack health insurance coverage, have coverage gaps, or skip or delay care because of limited personal finances might face increased risk for poor physical and mental health and premature mortality.

**Period Covered:**

2014.

**Description of System:**

The Behavioral Risk Factor Surveillance System (BRFSS) is an ongoing, state-based, landline- and cellular-telephone survey of noninstitutionalized adults aged ≥18 years residing in the United States. Data are collected from states, the District of Columbia, and participating U.S. territories on health risk behaviors, chronic health conditions, health care access, and use of clinical preventive services (CPS). An optional Health Care Access module was included in the 2014 BRFSS.

This report summarizes 2014 BRFSS data from all 50 states and the District of Columbia on health care access and use of selected CPS recommended by the U.S. Preventive Services Task Force or the Advisory Committee on Immunization Practices among working-aged adults (aged 18–64 years), by state, state Medicaid expansion status, expanded geographic region, and federal poverty level (FPL). This report also provides analysis of primary type of health insurance coverage at the time of interview, continuity of health insurance coverage during the preceding 12 months, and other health care access measures (i.e., unmet health care need because of cost, unmet prescription need because of cost, medical debt [medical bills being paid off over time], number of health care visits during the preceding year, and satisfaction with received health care) from 43 states that included questions from the optional BRFSS Health Care Access module.

**Results:**

In 2014, health insurance coverage and other health care access measures varied substantially by state, state Medicaid expansion status, expanded geographic region (i.e., states categorized geographically into nine regions), and FPL category. The following proportions refer to the range of estimated prevalence for health insurance and other health care access measures by examined geographical unit (unless otherwise specified), as reported by respondents. Among adults with health insurance coverage, the range was 70.8%–94.5% for states, 78.8%–94.5% for Medicaid expansion states, 70.8%–89.1% for nonexpansion states, 73.3%–91.0% for expanded geographic regions, and 64.2%–95.8% for FPL categories. Among adults who had a usual source of health care, the range was 57.2%–86.6% for states, 57.2%–86.6% for Medicaid expansion states, 61.8%–83.9% for nonexpansion states, 64.4%–83.6% for expanded geographic regions, and 61.0%–81.6% for FPL categories. Among adults who received a routine checkup, the range was 52.1%–75.5% for states, 56.0%–75.5% for Medicaid expansion states, 52.1%–71.1% for nonexpansion states, 56.8%–70.2% for expanded geographic regions, and 59.9%–69.2% for FPL categories. Among adults who had unmet health care need because of cost, the range was 8.0%–23.1% for states, 8.0%–21.9% for Medicaid expansion states, 11.9%–23.1% for nonexpansion states, 11.6%–20.3% for expanded geographic regions, and 5.3%–32.9% for FPL categories. Estimated prevalence of cancer screenings, influenza vaccination, and having ever been tested for human immunodeficiency virus also varied by state, state Medicaid expansion status, expanded geographic region, and FPL category.

The prevalence of insurance coverage varied by approximately 25 percentage points among racial/ethnic groups (range: 63.9% among Hispanics to 88.4% among non-Hispanic Asians) and by approximately 32 percentage points by FPL category (range: 64.2% among adults with household income <100% of FPL to 95.8% among adults with household income >400% of FPL). The prevalence of unmet health care need because of cost varied by nearly 14 percentage points among racial/ethnic groups (range: 11.3% among non-Hispanic Asians to 25.0% among Hispanics), by approximately 17 percentage points among adults with and without disabilities (30.8% versus 13.7%), and by approximately 28 percentage points by FPL category (range: 5.3% among adults with household income >400% of FPL to 32.9% among adults with household income <100% of FPL).

Among the 43 states that included questions from the optional module, a majority of adults reported private health insurance coverage (63.4%), followed by public health plan coverage (19.4%) and no primary source of insurance (17.1%). Financial barriers to health care (unmet health care need because of cost, unmet prescribed medication need because of cost, and medical bills being paid off over time [medical debt]) were typically lower among adults in Medicaid expansion states than those in nonexpansion states regardless of source of insurance. Approximately 75.6% of adults reported being continuously insured during the preceding 12 months, 12.9% reported a gap in coverage, and 11.5% reported being uninsured during the preceding 12 months. The largest proportion of adults reported ≥3 visits to a health care professional during the preceding 12 months (47.3%), followed by 1–2 visits (37.1%), and no health care visits (15.6%). Adults in expansion and nonexpansion states reported similar levels of satisfaction with received health care by primary source of health insurance coverage and by continuity of health insurance coverage during the preceding 12 months.

**Interpretation:**

This report presents for the first time estimates of population-based health care access and use of CPS among adults aged 18–64 years. The findings in this report indicate substantial variations in health insurance coverage; other health care access measures; and use of CPS by state, state Medicaid expansion status, expanded geographic region, and FPL category. In 2014, health insurance coverage, having a usual source of care, having a routine checkup, and not experiencing unmet health care need because of cost were higher among adults living below the poverty level (i.e., household income <100% of FPL) in states that expanded Medicaid than in states that did not. Similarly, estimates of breast and cervical cancer screening and influenza vaccination were higher among adults living below the poverty level in states that expanded Medicaid than in states that did not. These disparities might be due to larger differences to begin with, decreased disparities in Medicaid expansion states versus nonexpansion states, or increased disparities in nonexpansion states.

**Public Health Action:**

BRFSS data from 2014 can be used as a baseline by which to assess and monitor changes that might occur after 2014 resulting from programs and policies designed to increase access to health care, reduce health disparities, and improve the health of the adult population. Post-2014 changes in health care access, such as source of health insurance coverage, attainment and continuity of coverage, financial barriers, preventive care services, and health outcomes, can be monitored using these baseline estimates.

## Introduction

The number of adults with health insurance coverage fluctuates because of economic conditions, demographics, geographic location, and policy changes that affect access to health care such as the 2010 Patient Protection and Affordable Care Act (ACA). Since the 2010 passage of ACA, approximately 20 million uninsured working-aged adults (aged 18–64 years) have gained health insurance coverage (*1*). During 2013–2014, when many of the major coverage provisions of ACA went into effect (e.g., creation of the Health Insurance Marketplace, barring coverage exclusions for pre-existing health conditions, expansion of Medicaid, establishment of tax credits, reductions in cost sharing, and other provisions to increase availability and affordability of coverage), the percentage of working-aged adults with health insurance increased by approximately 3 percentage points, according to the Behavioral Risk Factor Surveillance System (BRFSS) and other surveys (*1*–*7*). During 2011–2014 at the state level, all but Hawaii, Maine, New Hampshire, and the District of Columbia experienced significant gains in health insurance coverage among working-aged adults (*3*). Under ACA, states have the option of expanding Medicaid to increase coverage, and the gains in health insurance coverage have occurred to varying degrees in Medicaid expansion states and nonexpansion states (*1*,*4*–*6*,*8*–*13*).

Health insurance coverage has been associated with increased access to clinical preventive services (CPS) and other medical services and, as a result, improvements in adult health (e.g., self-reported health and clinical depression), mortality, and financial security (*12*,*14*–*33*). However, adults who lack health insurance coverage or have inadequate coverage, gaps in coverage, or difficulties accessing or navigating the U.S. health care system might delay or forgo CPS and other needed medical care (*34*–*38*). Such delays might lead to poor physical and mental health, premature mortality, increased health disparities, and increased financial risk, particularly among racial/ethnic minorities, persons with disabilities, and other vulnerable population groups (e.g., homeless persons, cancer survivors, or pregnant women) (*35*,*36*,*38*–*42*). In addition to increasing access to affordable insurance coverage, ACA requires most health plans to cover CPS without copays or deductibles for services given an A or B recommendation by the U.S. Preventive Services Task Force (USPSTF) (https://www.uspreventiveservicestaskforce.org/Page/Name/uspstf-a-and-b-recommendations), vaccinations recommended by the Advisory Committee on Immunization Practices (ACIP) (https://www.cdc.gov/vaccines/hcp/acip-recs/), and preventive health care services recommended by the Health Resources and Services Administration for women (https://www.hrsa.gov/womensguidelines/) and children (https://www.aap.org/en-us/Documents/periodicity_schedule.pdf); ACA also includes provisions aimed at improving the quality of care (*43*).

Population health indicators that can be used to assess national and state policies intending to improve the availability of health insurance coverage, access to CPS and other medical services, and the quality of care received are essential measures in monitoring and improving the health of adults in the United States (*5*,*44*). Since 1984, national, state, and local public health professionals and policymakers have used population-based data from the BRFSS to monitor health of the adult population. Specifically, BRFSS has provided uniformly collected state-specific data on demographic characteristics, health-related behaviors, chronic health conditions, health care access, and use of preventive health services that are associated with the leading causes of death and disability in the United States.

This report summarizes 2014 BRFSS data on health care access and use of selected CPS recommended by USPSTF and ACIP among working-aged adults (aged 18–64 years), by state, state Medicaid expansion status, expanded geographic region, and federal poverty level (FPL). This report also provides analysis of primary type of health insurance coverage at the time of interview, continuity of health insurance coverage during the preceding 12 months, and other health care access measures (i.e., cost barrier to prescribed medication need, medical bills being paid off over time [medical debt], number of health care visits in the preceding 12 months, and satisfaction with received health care) in states that included an optional Health Care Access module in the 2014 BRFSS survey. The state-specific BRFSS data were collected during the first year that several provisions of ACA went into effect; therefore, findings in this report might provide public health professionals, policymakers, and researchers a baseline to monitor effects that might occur after 2014 of programs and policies designed to increase access to health care, reduce health disparities, and improve the health of the adult population.

## Methods

**FIGURE 1:**
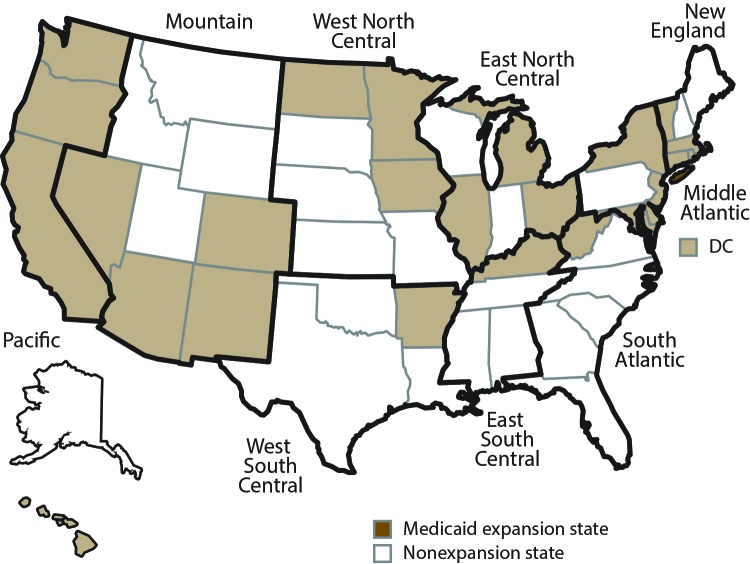
Expanded geographic regions* and state Medicaid expansion status^†^ as of January 1, 2014 **Abbreviation:** DC = District of Columbia. * Expanded geographic regions are the four U.S. census regions subdivided into nine regions. For this report, the nine census regions were modified by moving Delaware, District of Columbia, and Maryland into the Middle Atlantic region. *New England:* Connecticut, Maine, Massachusetts, New Hampshire, Rhode Island, and Vermont. *Middle Atlantic:* Delaware, District of Columbia, Maryland, New Jersey, New York, and Pennsylvania. *East North Central:* Illinois, Indiana, Michigan, Ohio, and Wisconsin. *West North Central:* Iowa, Kansas, Minnesota, Missouri, Nebraska, North Dakota, and South Dakota. *South Atlantic:* Florida, Georgia, North Carolina, South Carolina, Virginia, and West Virginia. *East South Central:* Alabama, Kentucky, Mississippi, and Tennessee. *West South Central:* Arkansas, Louisiana, Oklahoma, and Texas. *Mountain:* Arizona, Colorado, Idaho, Montana, Nevada, New Mexico, Utah, and Wyoming. *Pacific:* Alaska, California, Hawaii, Oregon, and Washington. ^†^ States were categorized by Medicaid expansion status as of January 1, 2014, into two groups: Medicaid expansion states (n = 26) and nonexpansion states (n = 25). *Medicaid expansion states:* Arizona, Arkansas, California, Colorado, Connecticut, Delaware, District of Columbia, Hawaii, Illinois, Iowa, Kentucky, Maryland, Massachusetts, Michigan, Minnesota, Nevada, New Jersey, New Mexico, New York, North Dakota, Ohio, Oregon, Rhode Island, Vermont, Washington, and West Virginia. *Nonexpansion states:* Alabama, Alaska, Florida, Georgia, Idaho, Indiana, Kansas, Louisiana, Maine, Mississippi, Missouri, Montana, Nebraska, New Hampshire, North Carolina, Oklahoma, Pennsylvania, South Carolina, South Dakota, Tennessee, Texas, Utah, Virginia, Wisconsin, and Wyoming.

To assess health insurance coverage, access to health care, and use of selected CPS recommended by USPSTF and ACIP by state, state Medicaid expansion status, expanded geographic region, and FPL among noninstitutionalized adults aged 18–64 years, CDC analyzed 2014 BRFSS data from 50 states and the District of Columbia (hereafter referred to as states) (Figure 1). To further assess health care access, CPS use, number of health care visits during the preceding 12 months, and satisfaction with received health care among working-aged adults by type of insurance coverage (i.e., public, private) and continuity of coverage during the preceding 12 months (i.e., continuously insured, gaps in coverage, uninsured) and state Medicaid expansion status, CDC analyzed 2014 data from 43 states that used questions from an optional BRFSS Health Care Access module. These states were Alabama, Alaska, Arizona, Colorado, Connecticut, Delaware, District of Columbia, Georgia, Idaho, Illinois, Indiana, Iowa, Kentucky, Louisiana, Maine, Maryland, Massachusetts, Michigan, Minnesota, Mississippi, Missouri,* Montana, Nebraska, Nevada, New Hampshire, New Jersey, New Mexico, New York, North Carolina, North Dakota, Ohio, Oklahoma, Oregon, Pennsylvania, Rhode Island, South Carolina, Tennessee, Utah, Vermont, Virginia, Washington, West Virginia, and Wisconsin.

### Project Description

BRFSS is an ongoing, state-based system of landline- and cellular-telephone health surveys (*45*,*46*). Since 1984, BRFSS has provided state-specific, population-based data on demographic characteristics, health risk behaviors, health care access, and use of CPS that are associated with the leading causes of illness, disability, and death in the United States. BRFSS uses a multistage sampling design to select a representative sample of the noninstitutionalized civilian population aged ≥18 years residing in states and participating U.S. territories. Additional details on survey methodology, random sampling procedures, design, and reliability and validity of measures used in BRFSS have been described elsewhere (*47*,*48*).

In 2014, a total of 456,119 adults aged ≥18 years completed interviews by landline and cellular telephones in all states. For data collected by landline telephone, 293,226 respondents completed the interview and the number of respondents ranged from 2,371 in Nevada to 12,962 in Nebraska (median: 4,981). For data collected by cellular telephone, 162,893 respondents completed the interview and the number of respondents ranged from 798 in the District of Columbia to 9,952 in Nebraska (median: 2,906). Response rates for BRFSS were calculated using the standard set by the American Association of Public Opinion Research response rate formula 4 (RR4), which is the number of respondents who completed the survey as a proportion of all eligible and likely eligible persons (*15*). For landline telephone data, the RR4 response rate ranged from 26.7% in California to 61.6% in Kentucky (median: 48.7%); for cellular telephone data, the RR4 response rate ranged from 22.2% in California to 60.0% in Alaska (median: 40.5%). For combined landline telephone and cellular telephone data, the weighted response rate (determined using a combination of the landline telephone response rate with the cellular telephone response rate proportional to the total sample used to collect the data for a state) ranged from 25.1% in California to 60.1% in South Dakota (median: 47.0%). Detailed information on response, cooperation, and refusal rates for landline and cellular telephone data collection, including data from participating territories, can be found in the BRFSS 2014 Summary Data Quality Report (*49*).

### Data Collection

Trained interviewers use a standardized BRFSS data collection process to collect uniform data (*50*). The standard BRFSS questionnaire consists of three sections: 1) core questions, 2) optional modules that include sets of questions on specific topics, and 3) state-added questions. Trained interviewers ask the same core questions of survey respondents (*45*). States can select from optional BRFSS modules and include state-added questions according to the specific needs and goals of the programs in their health departments.

In 2014, all states collected answers to the core questions via landline and cellular telephones, as did 43 states that asked questions from the optional Health Care Access module. As in previous years, 2014 BRFSS included four health care access measures (i.e., health insurance coverage, usual health care provider, cost barrier to health care need, and receipt of a routine checkup) and selected CPS recommended by USPSTF (i.e., colorectal, breast, and cervical cancer screenings and human immunodeficiency virus [HIV] testing) and for selected vaccinations recommended by ACIP (e.g., influenza vaccination) in the core survey module (*51*). In 2014, BRFSS included an optional Health Care Access module that assessed primary source of health insurance coverage, continuity of health insurance during the preceding 12 months, cost barrier to prescribed medication need, medical debt (i.e., medical bills being paid off over time), number of health care visits during the preceding 12 months, and satisfaction with received health care (*51*).

### Questionnaire

For this report, four health care access measures and five CPS recommended by USPSTF or ACIP for which data are collected in the core section of BRFSS by all states were analyzed (Box 1). In addition, the following health care access measures derived from questions in the optional Health Care Access module conducted in 43 states were summarized: primary type of health insurance coverage at the time of interview, continuity of health insurance coverage during the preceding 12 months, cost barrier to prescribed medication, medical debt, number of health care visits during the preceding 12 months, and satisfaction with received health care (Box 2).

BOX 1 Characteristics of measures of health care access and use of clinical preventive services* from 2014 Behavioral Risk Factor Surveillance System core questionnaire

**Table:** 

**Measure**	**Definition**	**Age group (yrs)**
**Health care access**		
Health insurance coverage	Had any kind of health care coverage at the time of interview	18−64
Usual source of health care	Had personal doctor(s) or health care provider(s)	18−64
Cost barrier to health care need	Had unmet health care need because of cost during preceding 12 months	18−64
Routine medical checkup	Received routine checkup within preceding 12 months	18−64
**Clinical preventive services**		
Breast cancer screening^†^	Received mammogram in past 2 years (women)	50−64
Cervical cancer screening	Received Pap test in past 3 years (women with an intact uterus)	21–64
Colorectal cancer screening	Received FOBT within the past year, or sigmoidoscopy within the past 5 years and an FOBT within the past 3 years, or colonoscopy within the past 10 years	50−64
HIV test	Ever received HIV test (excluding tests done for blood donations)	18−64
Influenza immunization	Received influenza vaccine in past year	18−64
**Abbreviations:** FOBT = fecal occult blood test; HIV = human immunodeficiency virus; Pap = Papanicolaou. * Affordable Care Act preventive services mandate (Patient Protection and Affordable Care Act PL 111–148 124 Stat. 110 Mar. 23, 2010 and PL 111–152 Stat. 1029 Mar. 30, 2010). ^†^ The Affordable Care Act requires coverage of mammograms beginning at age 40 years.

BOX 2 Optional Health Care Access module questions — Behavioral Risk Factor Surveillance System, 2014*

1) Do you have Medicare?

2) What is the primary source of your health care coverage? Is it … a plan purchased through an employer or union (includes plans purchased through another person’s employer); a plan that you or another family member buys on your own; Medicare; Medicaid or other state program; TRICARE (formerly CHAMPUS), VA, or military; Alaska Native, Indian Health Service, Tribal Health Services; or some other source; none (no coverage)?

**Interviewer note:** If the respondent indicates that they purchased health insurance through the Health Insurance Marketplace (name of state marketplace), ask if it was a private health insurance plan purchased on their own or by a family member (private) or if they received Medicaid (state plan)? If purchased on their own (or by a family member), select 02 (a plan that you or another family member buys on your own), if Medicaid select 04 (Medicaid or other state program).

3) Other than cost, there are many other reasons people delay getting needed medical care. Have you delayed getting needed medical care for any of the following reasons in the past 12 months? Select the most important reason. You couldn’t get through on the telephone; you couldn’t get an appointment soon enough; once you got there, you had to wait too long to see the doctor; the (clinic/doctor’s) office wasn’t open when you got there; or you didn’t have transportation.

4a) If respondent has health care coverage, ask: In the past 12 months was there any time when you did not have any health insurance or coverage?

4b) If respondent does not have health care coverage, doesn’t know, or refused to answer, ask: About how long has it been since you last had health care coverage?

5) How many times have you been to a doctor, nurse, or other health professional in the past 12 months?

6) Was there a time in the past 12 months when you did not take your medication as prescribed because of cost? Do not include over-the-counter (OTC) medication.

7) In general, how satisfied are you with the health care you received? Would you say very satisfied, somewhat satisfied, or not at all satisfied?

8) Do you currently have any medical bills that are being paid off over time?

**Interviewer note:** This could include medical bills being paid off with a credit card, through personal loans, or bill paying arrangements with hospitals or other providers. The bills can be from earlier years as well as this year.

**Interviewer note:** Health care bills can include medical, dental, physical therapy, and/or chiropractic cost.

**Abbreviation:** VA = Veterans Affairs.

* In this report, data were analyzed from 41 states and the District of Columbia that fielded the questions as an optional BRFSS module and for one state (Missouri) that fielded the questions as state-added questions. The data for 41 states and the District of Columbia are available as public use data sets on the BRFSS website at https://www.cdc.gov/brfss/annual_data/annual_2014.html. Data from Missouri are available from the Missouri Department of Health and Senior Services.

In the survey’s core section, respondents were asked whether they had any kind of health care coverage, including health insurance, prepaid plans such as health maintenance organizations, government plans such as Medicare, or Indian Health Service (IHS). Respondents who answered yes were defined as having health insurance coverage at the time of interview. Responses for having a usual health care provider were dichotomized into one or more and none, and responses for having had a routine checkup within the preceding 12 months were dichotomized into within the past year or not within the past year. Cost barrier to health care need during the preceding 12 months was assessed with the question “Was there a time in the past 12 months when you needed to see a doctor but could not because of cost?” Respondents who answered yes were defined as having unmet health care need because of cost during the preceding 12 months.

To summarize these measures, states were categorized by Medicaid expansion status and expanded geographic region and respondents by FPL. Under ACA, effective January 1, 2014, states can choose at any time to expand Medicaid eligibility to adults in households with incomes ≤138% of FPL. For analysis, states were categorized by Medicaid expansion status as of January 1, 2014, into two groups: Medicaid expansion states (n = 26) and nonexpansion states (n = 25) (Figure 1). The Medicaid expansion states were Arizona, Arkansas, California, Colorado, Connecticut, Delaware, District of Columbia, Hawaii, Illinois, Iowa, Kentucky, Maryland, Massachusetts, Michigan,^†^ Minnesota, Nevada, New Jersey, New Mexico, New York, North Dakota, Ohio, Oregon, Rhode Island, Vermont, Washington, and West Virginia. The nonexpansion states were Alabama, Alaska, Florida, Georgia, Idaho, Indiana, Kansas, Louisiana, Maine, Mississippi, Missouri, Montana, Nebraska, New Hampshire,^§^ North Carolina, Oklahoma, Pennsylvania, South Carolina, South Dakota, Tennessee, Texas, Utah, Virginia, Wisconsin, and Wyoming.

Similar to methodology used by the National Center for Health Statistics (*7*) and other researchers (*52*), the four U.S. census regions were subdivided into nine geographic regions. The regions were then modified by moving Delaware, the District of Columbia, and Maryland into the Middle Atlantic region. The expanded geographic regions and respective states were New England (Connecticut, Maine, Massachusetts, New Hampshire, Rhode Island, and Vermont); Middle Atlantic (Delaware, District of Columbia, Maryland, New Jersey, New York, and Pennsylvania); East North Central (Illinois, Indiana, Michigan, Ohio, and Wisconsin); West North Central (Iowa, Kansas, Minnesota, Missouri, Nebraska, North Dakota, and South Dakota); South Atlantic (Florida, Georgia, North Carolina, South Carolina, Virginia, and West Virginia); East South Central (Alabama, Kentucky, Mississippi, and Tennessee); West South Central (Arkansas, Louisiana, Oklahoma, and Texas); Mountain (Arizona, Colorado, Idaho, Montana, Nevada, New Mexico, Utah, and Wyoming); and Pacific (Alaska, California, Hawaii, Oregon, and Washington) (Figure 1).

ACA provides tax credits to assist with the cost of health insurance premiums for persons in households with incomes between 100% and 400% of FPL. In Medicaid expansion states, persons in households with incomes between 0% and 400% of FPL are eligible for coverage options through tax credits and expanded Medicaid eligibility (≤138% of FPL). In nonexpansion states, persons in households with incomes between 100% and 400% of FPL are eligible for a tax credit to assist with the cost of coverage, but no new financial assistance was provided for those with household incomes below 100% of FPL. FPL categories for 2014 tax credit eligibility were determined on the basis of the ratio of the adult’s annual household income to the appropriate simplified 2013 federal poverty threshold (given family size and number of children) defined by FPL Guidelines for the Health Insurance Marketplace (*53*). Three core variables were used to calculate federal poverty threshold: 1) annual household income, 2) number of adults (1–14) in the household, and 3) number of children (≥0) in the household. Federal poverty thresholds were then used to categorize respondents into four FPL categories: 1) <100% of FPL, 2) ≥100%–≤400% of FPL, 3) >400% of FPL, and 4) unknown. Additional details on this variable have been described elsewhere (*54*).

The following sociodemographic characteristics were described overall and by state for two health care access measures (health insurance at the time of interview and unmet health care need because of cost): age (18–24, 25–44, and 45–64 years); sex; race/ethnicity (non-Hispanic white, non-Hispanic black, Hispanic, non-Hispanic Asian, non-Hispanic American Indian or Alaskan Native [AIAN], non-Hispanic Native Hawaiian or other Pacific Islander [NHPI], non-Hispanic multiple race, and other); educational attainment (less than high school, high school, and greater than high school); marital status (married, previously married [including divorced, widowed, and separated] and never married or member of an unmarried couple); employment status (employed, unemployed, or not in labor market [including homemaker, student, retired, and unable to work]); FPL (<100%, ≥100%–≤400%, >400%, or unknown); and self-reported disability indicated by a yes response to either of two questions: 1) “Are you limited in any way in any activities because of physical, mental, or emotional problems?” and 2) “Do you now have any health problem that requires you to use special equipment, such as a cane, a wheelchair, a special bed, or a special telephone?”

Data from 43 states that administered questions from the optional Health Care Access module were analyzed for the following measures: health care access and CPS measures (Box 1); cost barrier to prescribed medication need during the preceding 12 months; medical bills being paid off over time (i.e., medical debt); number of health care visits during the preceding 12 months; and satisfaction with received health care, by type of insurance coverage (i.e., none, public, private) and continuity of coverage during the preceding year (i.e., continuously insured, a gap or gaps in coverage, uninsured), by state Medicaid expansion status and FPL. To classify respondents by type of health insurance, respondents were asked the core question that assessed health insurance coverage at the time of interview and the optional module question: “What is the primary source of your health care coverage? Is it a plan purchased through an employer or union (includes plans purchased through another person’s employer); a plan that you or another family member buys on your own; Medicare; Medicaid or other state program; TRICARE (formerly CHAMPUS), VA [Veterans Affairs], or military; Alaska Native, Indian Health Service, Tribal Health Services; or some other source?” Respondents were then categorized into three groups: 1) no primary source of health care coverage, 2) public health plan coverage, and 3) private health insurance coverage. No primary source of health care coverage included respondents who answered no to the core question as well as respondents who answered yes but, subsequently, indicated they had no primary source of health care coverage (i.e., none). The public health plan coverage category was defined as Medicare, Medicaid or other state program, military (i.e., TRICARE, VA [Veterans Affairs], or military), IHS (i.e., Alaska Native, Indian Health Service, or Tribal Health Services), or some other source. The private health insurance coverage category was defined as an employer-based plan or a self-purchased plan (i.e., a plan that a person or another family member buys on their own).

To classify respondents by continuity of coverage during the preceding year, the core question was used that assessed health insurance coverage at the time of interview, as well as two optional module questions: 1) “In the past 12 months was there any time when you did not have any health insurance or coverage?” and 2) “About how long has it been since you last had health care coverage?” Respondents were then categorized into three groups: 1) continuously insured in the preceding 12 months before interview, 2) having a gap in insurance in the preceding 12 months before interview, and 3) having no health insurance for >12 months. The continuously insured category included respondents who were insured and had not been uninsured at any time in the preceding 12 months. The gap in insurance category included respondents who were insured but had been uninsured at some time during the preceding 12 months or who were uninsured but had been insured at some time during the preceding 12 months. The no health insurance category included respondents who were uninsured and had been uninsured for >12 months.

The optional Health Care Access module included asking respondents how many times they had been to a health care professional during the preceding year. Number of health care visits was categorized as none, 1–2, and ≥3. Among respondents who had ≥1 health care visit, health care satisfaction was assessed using the question “In general, how satisfied are you with the health care you received? Would you say very satisfied, satisfied, or not at all satisfied?” Data from the optional module were also examined for the following financial-related barriers to medical care: cost barrier to prescribed medication need during the preceding 12 months and health care bills that are being paid off over time (Box 2).

### Data Weighting and Statistical Analysis

An iterative proportional fitting (or raking) was used to weight each state in the BRFSS. BRFSS raking includes categories of age by gender, detailed race/ethnicity groups, educational attainment, marital status, regions within states, sex by race/ethnicity, telephone source, home renter or owner status, and age group by race/ethnicity. Raking adjusts the data so that groups underrepresented in the sample are more accurately represented in the final data set, and this weighting method has been shown to reduce bias within estimates (*47*). In 2014, the inclusion of cellular telephone respondents who also have landline telephones in their household required an adjustment to the design weights to account for the overlapping sample (*55*).

For the optional Health Care Access module, four states (Colorado, Indiana, Maine, and Oklahoma) used multiple questionnaires to collect data on a subset of the state sample rather than on the entire sample. For these states, the survey weights provided in the multiple questionnaire data files were used. Details on the weighting methodology and the weights to be used for each of these states have been described elsewhere (*54*,*56*).

To account for the complex sampling design of BRFSS, all the prevalence estimates were computed on the basis of a statistical analysis (SAS version 9.3, SAS Institute Inc., Cary, North Carolina, USA; SAS-callable SUDAAN version 11.0.1, RTI International, Research Triangle Park, North Carolina, USA) using weights and strata. Prevalence estimates are presented as percentages with 95% confidence intervals (CI). Both crude and age-standardized (standardized to the 2000 projected U.S. population) (*57*) prevalence estimates are provided in all tables but, unless otherwise noted, only age-standardized estimates are provided in the text. Prevalence estimates are not presented if the sample size for the denominator was <50 or the relative standard error (RSE) was >30%. Prevalence estimates are flagged as unstable if the RSE is 20%–30%. RSE was calculated by dividing the standard error by the estimated prevalence and multiplying by 100 (for percent). Differences were considered statistically significant if the CIs of the age-standardized estimates did not overlap. While the use of CIs in this way is conservative, it might identify some estimates as similar when they are statistically different.

## Results

### Health Care Access, Overall and by State

#### Health Insurance Coverage

**FIGURE 2:**
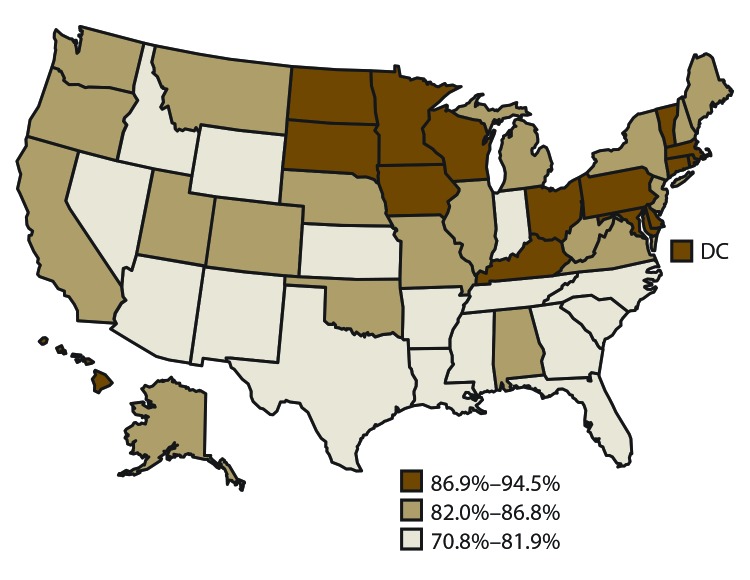
Age-standardized* prevalence estimates of current health insurance coverage^†^ among adults aged 18–64 years — Behavioral Risk Factor Surveillance System, United States,^§^ 2014 **Abbreviation:** DC = District of Columbia. * Age standardized to the 2000 projected population for the United States. ^†^ Survey question used to assess current health insurance coverage: Do you have any kind of health care coverage, including health insurance, prepaid plans such as health maintenance organizations, government plans such as Medicare, or Indian Health Service? ^§^ States are divided into tertiles.

**FIGURE 3:**
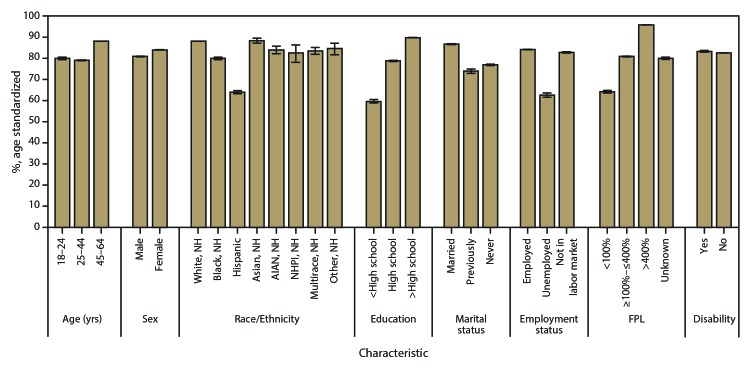
Age-standardized* prevalence estimates of current health insurance coverage among adults aged 18–64 years, by sociodemographic characteristics — Behavioral Risk Factor Surveillance System, United States, 2014 **Abbreviations**: AIAN = American Indian or Alaskan Native; FPL = federal poverty level; NH = non-Hispanic; NHPI = Native Hawaiian or other Pacific Islander. * Age standardized to the 2000 projected U.S. population, except for age groups. Bars represent 95% confidence intervals.

Of 299,157 BRFSS respondents aged 18–64 years, 297,734 answered the question on insurance coverage; among these adults, 82.4% (n = 262,611; N = 163.8 million) reported having health insurance at the time of the interview. The median estimated prevalence of adults who were insured at the time of interview was 84.2% but varied by state. For example, 94.5% and 91.2% were insured in Massachusetts and Vermont, respectively, whereas 74.6% and 70.8% were insured in Georgia and Texas, respectively (Table 1) (Figure 2). The estimated prevalence of insurance coverage was higher for adults aged 45–64 years (88.0%) than for adults aged 18–24 years (79.9%) and 25–44 years (79.0%) (Figure 3). The estimated prevalence of insurance coverage was higher among women (84.0%) than men (80.8%). By race/ethnicity, the estimated prevalence for adults insured at the time of interview was as follows: 63.9% (Hispanic), 80.0% (non-Hispanic black), 82.6% (non-Hispanic NHPI), 83.5% (non-Hispanic multiple race), 84.0% (non-Hispanic AIAN), 84.6% (non-Hispanic other race), 88.0% (non-Hispanic white), and 88.4% (non-Hispanic Asian). The estimated prevalence of insurance coverage at the time of interview increased with increasing level of educational attainment: 59.6% of adults with less than high school education, 78.7% of adults with a high school education, and 89.8% of adults with greater than high school education had insurance coverage. Married adults (86.7%) were more likely to be insured at the time of interview than those who were previously married (divorced, separated, widowed) (73.9%) or never married (76.9%). A majority of employed adults (84.1%) were insured at the time of interview, whereas 62.5% of unemployed adults were insured. Adults with household income <100% of FPL were less likely to be insured (64.2%) than adults in other income groups (80.8% and 95.8% among adults with household income ≥100%–≤400% of FPL and >400% of FPL, respectively). No difference in the estimated prevalence of insurance coverage by disability status was found. Unadjusted state-specific estimates of insurance coverage by sociodemographic characteristics are available (supplemental Table S1 https://stacks.cdc.gov/view/cdc/43255).

**TABLE 1:** Crude and age-standardized* prevalence estimates of health insurance coverage, usual source of health care, routine checkup within the preceding 12 months, and cost barrier to health care need during the preceding 12 months among adults aged 18–64 years, by state Medicaid expansion status — Behavioral Risk Factor Surveillance System, United States, 2014

State Medicaid expansion status	n^†^	Insured at the time of interview^§^		Usual source of health care		Routine checkup within preceding 12 mos		Cost barrier to health care need during preceding 12 mos	
		Crude	Age standardized	Crude	Age standardized	Crude	Age standardized	Crude	Age standardized
		%^¶^ (95% CI)	% (95% CI)	% (95% CI)	% (95% CI)	% (95% CI)	% (95% CI)	% (95% CI)	% (95% CI)
Medicaid expansion states**	**150,756**								
Arizona	8,282	82.1 (80.7–83.5)	81.8 (80.3–83.3)	66.6 (65.0–68.3)	65.9 (64.2–67.6)	58.1 (56.4–59.8)	57.5 (55.8–59.2)	18.1 (16.8–19.5)	18.2 (16.9–19.7)
Arkansas	2,845	79.9 (77.5–82.1)	79.3 (76.8–81.6)	73.3 (70.7–75.8)	72.0 (69.3–74.5)	61.4 (58.7–64.0)	60.5 (57.8–63.2)	21.7 (19.6–24.0)	21.9 (19.7–24.3)
California	6,612	82.4 (81.2–83.6)	82.1 (80.8–83.3)	70.5 (69.1–71.9)	69.7 (68.2–71.1)	62.8 (61.3–64.3)	62.3 (60.8–63.8)	15.2 (14.2–16.4)	15.4 (14.3–16.5)
Colorado	9,108	84.8 (83.8–85.8)	84.3 (83.2–85.3)	72.4 (71.2–73.6)	71.4 (70.1–72.6)	59.0 (57.7–60.2)	58.3 (57.0–59.6)	14.9 (14.0–15.9)	15.1 (14.1–16.1)
Connecticut	5,317	89.6 (88.3–90.8)	88.8 (87.3–90.1)	81.3 (79.7–82.7)	79.3 (77.6–80.9)	68.3 (66.5–70.1)	67.0 (65.1–68.8)	12.9 (11.8–14.2)	13.4 (12.1–14.8)
Delaware	2,732	89.3 (87.6–90.8)	88.6 (86.7–90.2)	83.4 (81.3–85.2)	82.2 (80.0–84.2)	69.4 (66.9–71.9)	68.2 (65.5–70.8)	12.5 (10.9–14.3)	12.6 (11.0–14.5)
District of Columbia	2,531	90.5 (87.9–92.5)	90.6 (88.1–92.6)	72.0 (68.9–74.8)	72.5 (69.6–75.3)	71.4 (68.5–74.2)	71.8 (68.9–74.5)	11.7 ( 9.6–14.2)	11.7 ( 9.6–14.1)
Hawaii	5,097	90.4 (89.1–91.5)	90.1 (88.7–91.3)	82.0 (80.4–83.5)	81.2 (79.6–82.8)	63.0 (61.1–64.8)	62.2 (60.3–64.1)	10.1 ( 9.0–11.3)	10.1 ( 9.0–11.3)
Illinois	3,410	85.7 (83.9–87.3)	85.1 (83.2–86.7)	77.7 (75.8–79.5)	76.9 (74.9–78.7)	65.4 (63.3–67.4)	64.8 (62.7–66.9)	13.9 (12.4–15.5)	14.1 (12.5–15.8)
Iowa	5,178	90.4 (89.3–91.5)	89.9 (88.6–91.0)	77.0 (75.4–78.5)	76.2 (74.5–77.8)	66.8 (65.1–68.4)	65.8 (64.0–67.5)	10.3 ( 9.3–11.5)	10.7 ( 9.6–11.9)
Kentucky	7,250	87.9 (86.4–89.2)	87.4 (85.8–88.8)	75.7 (73.9–77.4)	74.4 (72.6–76.2)	67.8 (66.0–69.6)	66.8 (64.9–68.7)	18.4 (16.9–19.9)	18.8 (17.2–20.4)
Maryland	7,891	89.1 (87.5–90.5)	88.2 (86.4–89.7)	79.7 (77.9–81.4)	78.4 (76.5–80.2)	72.0 (70.2–73.8)	71.2 (69.2–73.0)	11.6 (10.3–13.0)	12.1 (10.7–13.6)
Massachusetts	10,001	94.7 (93.9–95.4)	94.5 (93.7–95.3)	87.5 (86.4–88.5)	86.6 (85.4–87.7)	75.2 (73.8–76.4)	74.2 (72.8–75.6)	9.5 ( 8.6–10.5)	9.7 ( 8.8–10.7)
Michigan	5,578	87.4 (86.2–88.5)	86.8 (85.5–88.0)	80.7 (79.3–82.0)	79.4 (77.8–80.8)	67.8 (66.2–69.3)	66.5 (64.8–68.1)	16.8 (15.6–18.1)	17.4 (16.0–18.8)
Minnesota	11,892	91.3 (90.5–91.9)	90.8 (90.0–91.6)	72.1 (71.1–73.1)	70.7 (69.6–71.7)	65.6 (64.6–66.7)	64.8 (63.6–65.8)	10.6 ( 9.9–11.3)	10.9 (10.1–11.6)
Nevada	2,467	79.4 (76.7–81.9)	78.8 (76.0–81.4)	58.5 (55.5–61.4)	57.2 (54.2–60.2)	59.0 (56.0–62.0)	58.4 (55.3–61.4)	20.1 (17.8–22.7)	20.3 (17.9–23.0)
New Jersey	9,095	85.0 (83.9–86.1)	84.2 (82.9–85.3)	78.5 (77.3–79.8)	77.2 (75.8–78.6)	72.3 (70.9–73.7)	71.3 (69.8–72.7)	15.9 (14.8–17.0)	16.2 (15.1–17.4)
New Mexico	5,977	81.2 (79.5–82.8)	80.4 (78.6–82.0)	64.5 (62.6–66.4)	63.3 (61.3–65.2)	57.7 (55.8–59.6)	56.7 (54.7–58.7)	19.9 (18.4–21.5)	20.3 (18.8–22.0)
New York	4,785	85.4 (84.0–86.7)	84.7 (83.2–86.1)	78.3 (76.8–79.8)	77.3 (75.7–78.9)	71.5 (69.8–73.1)	70.6 (68.9–72.3)	15.8 (14.5–17.2)	16.3 (14.9–17.7)
North Dakota	5,009	89.6 (88.0–91.0)	89.5 (87.9–90.9)	66.1 (63.9–68.2)	65.5 (63.3–67.6)	59.7 (57.5–61.8)	59.2 (57.0–61.4)	7.9 ( 6.8–9.3)	8.0 ( 6.9–9.4)
Ohio	6,941	87.6 (86.2–88.8)	87.1 (85.6–88.5)	76.3 (74.6–77.9)	74.8 (73.0–76.5)	66.0 (64.3–67.7)	64.5 (62.7–66.3)	15.1 (13.9–16.5)	15.3 (13.9–16.7)
Oregon	3,199	86.5 (84.8–88.1)	85.9 (84.1–87.6)	72.7 (70.6–74.6)	71.2 (69.0–73.3)	56.9 (54.7–59.1)	56.0 (53.7–58.3)	17.1 (15.5–18.8)	17.6 (15.9–19.4)
Rhode Island	4,270	90.3 (88.7–91.7)	89.7 (88.0–91.2)	83.7 (81.9–85.4)	82.6 (80.6–84.4)	76.7 (74.8–78.5)	75.5 (73.5–77.5)	13.7 (12.2–15.3)	14.1 (12.5–15.8)
Vermont	4,659	91.7 (90.6–92.6)	91.2 (90.0–92.3)	84.2 (82.7–85.5)	83.0 (81.4–84.4)	63.3 (61.6–65.0)	61.6 (59.8–63.4)	10.5 ( 9.4–11.6)	10.6 ( 9.6–11.8)
Washington	6,465	87.1 (85.8–88.3)	86.6 (85.2–87.9)	70.4 (68.8–72.0)	68.9 (67.2–70.5)	60.4 (58.8–62.0)	59.6 (57.9–61.3)	14.0 (12.9–15.2)	14.2 (13.1–15.5)
West Virginia	4,165	87.0 (85.6–88.3)	86.2 (84.7–87.6)	72.6 (70.9–74.3)	70.4 (68.6–72.2)	72.7 (71.0–74.4)	71.2 (69.3–73.0)	19.7 (18.2–21.2)	20.4 (18.9–22.1)
*Median*		*87.5*	*87.0*	*76.0*	*74.6*	*65.8*	*64.8*	*14.5*	*14.7*
*Range*		*79.4–94.7*	*78.8–94.5*	*58.5–87.5*	*57.2–86.6*	*56.9–76.7*	*56.0–75.5*	*7.9–21.7*	*8.0–21.9*
**Medicaid nonexpansion states^††^**	**148,401**								
Alabama	5,516	82.7 (81.1–84.1)	81.9 (80.2–83.4)	71.6 (69.8–73.3)	70.3 (68.5–72.1)	67.7 (65.8–69.4)	66.7 (64.8–68.6)	20.3 (18.8–21.8)	20.5 (19.0–22.1)
Alaska	3,400	83.0 (81.0–84.8)	82.6 (80.6–84.4)	62.9 (60.5–65.2)	61.8 (59.4–64.1)	54.6 (52.2–56.9)	53.9 (51.5–56.3)	13.4 (11.8–15.1)	13.6 (12.0–15.4)
Florida	5,504	77.3 (75.8–78.9)	76.7 (75.0–78.3)	69.8 (68.1–71.4)	68.0 (66.3–69.7)	67.3 (65.7–68.9)	66.3 (64.6–68.0)	21.8 (20.4–23.3)	22.1 (20.6–23.7)
Georgia	4,162	75.2 (73.2–77.0)	74.6 (72.6–76.4)	67.2 (65.1–69.1)	66.1 (64.1–68.1)	68.0 (66.0–69.9)	67.3 (65.3–69.3)	21.5 (19.8–23.2)	21.7 (20.0–23.5)
Idaho	3,552	79.9 (77.9–81.7)	79.4 (77.4–81.3)	65.5 (63.2–67.8)	64.7 (62.3–67.0)	52.9 (50.6–55.2)	52.1 (49.7–54.4)	18.3 (16.5–20.2)	18.7 (16.9–20.6)
Indiana	7,365	82.1 (80.9–83.3)	81.4 (80.0–82.6)	76.8 (75.5–78.1)	75.6 (74.1–76.9)	60.5 (59.0–62.0)	59.5 (58.0–61.1)	17.9 (16.7–19.1)	18.4 (17.2–19.7)
Kansas	9,319	82.5 (81.4–83.4)	81.9 (80.8–82.9)	77.1 (75.9–78.1)	76.2 (75.1–77.3)	63.4 (62.2–64.6)	62.5 (61.2–63.7)	14.5 (13.6–15.4)	14.8 (13.9–15.8)
Louisiana	4,698	77.4 (75.8–78.9)	76.9 (75.3–78.5)	69.8 (68.1–71.5)	68.8 (67.1–70.6)	71.8 (70.1–73.4)	71.1 (69.4–72.8)	19.9 (18.5–21.4)	20.1 (18.6–21.6)
Maine	5,784	86.1 (84.8–87.3)	85.5 (84.0–86.9)	85.3 (83.9–86.6)	83.9 (82.3–85.3)	67.2 (65.5–68.8)	65.2 (63.3–67.0)	13.4 (12.2–14.6)	13.8 (12.5–15.2)
Mississippi	2,632	77.1 (74.7–79.3)	76.6 (74.1–78.9)	69.1 (66.6–71.6)	68.6 (66.1–71.0)	68.9 (66.4–71.3)	68.4 (65.8–70.8)	22.7 (20.7–25.0)	23.1 (20.9–25.4)
Missouri	4,304	83.9 (82.2–85.5)	83.2 (81.4–84.9)	75.0 (73.0–76.9)	73.7 (71.6–75.7)	62.1 (60.0–64.1)	60.8 (58.6–62.9)	16.2 (14.7–17.8)	16.8 (15.2–18.5)
Montana	4,684	83.9 (82.2–85.4)	83.5 (81.8–85.1)	65.4 (63.4–67.3)	63.7 (61.6–65.8)	58.4 (56.4–60.4)	57.4 (55.3–59.5)	14.0 (12.6–15.5)	14.1 (12.7–15.7)
Nebraska	14,554	84.8 (83.7–85.8)	84.2 (83.1–85.2)	76.1 (74.9–77.3)	75.3 (74.1–76.5)	58.6 (57.3–59.9)	57.8 (56.4–59.1)	13.8 (12.8–14.7)	14.1 (13.2–15.1)
New Hampshire	3,981	85.9 (84.2–87.5)	85.0 (83.0–86.7)	82.0 (80.0–83.8)	80.1 (78.0–82.1)	64.6 (62.5–66.8)	62.1 (59.8–64.3)	12.9 (11.5–14.5)	13.5 (12.0–15.3)
North Carolina	4,960	80.2 (78.9–81.5)	79.3 (77.9–80.7)	71.7 (70.2–73.2)	70.4 (68.8–71.9)	71.8 (70.3–73.3)	70.7 (69.1–72.2)	18.7 (17.5–20.0)	18.9 (17.6–20.3)
Oklahoma	5,422	82.9 (81.5–84.1)	82.4 (81.0–83.7)	70.5 (68.9–72.1)	69.7 (68.1–71.3)	59.2 (57.5–60.8)	58.5 (56.8–60.2)	17.6 (16.4–19.0)	17.9 (16.6–19.3)
Pennsylvania	7,013	87.4 (86.2–88.6)	86.9 (85.5–88.1)	81.7 (80.3–83.0)	80.2 (78.7–81.6)	67.9 (66.4–69.4)	66.5 (64.9–68.1)	14.2 (13.0–15.4)	14.7 (13.5–16.1)
South Carolina	7,034	78.7 (77.4–80.1)	78.1 (76.7–79.5)	72.0 (70.5–73.5)	70.7 (69.2–72.2)	62.3 (60.8–63.9)	61.2 (59.6–62.8)	21.3 (20.1–22.6)	21.7 (20.4–23.1)
South Dakota	4,874	87.8 (85.8–89.5)	87.6 (85.5–89.4)	71.7 (69.3–73.9)	70.7 (68.3–73.0)	65.3 (62.9–67.6)	64.6 (62.1–67.0)	11.7 (10.2–13.4)	11.9 (10.3–13.8)
Tennessee	3,137	82.4 (80.3–84.3)	81.8 (79.6–83.8)	70.9 (68.5–73.2)	69.6 (67.1–71.9)	70.3 (67.9–72.6)	69.4 (66.9–71.8)	18.2 (16.4–20.1)	18.1 (16.3–20.1)
Texas	10,086	71.0 (69.6–72.4)	70.8 (69.3–72.2)	62.6 (61.0–64.1)	62.0 (60.5–63.5)	63.5 (62.0–65.0)	63.1 (61.6–64.6)	19.8 (18.6–21.1)	19.9 (18.7–21.2)
Utah	11,430	83.9 (83.0–84.7)	84.1 (83.2–84.9)	67.7 (66.6–68.7)	68.3 (67.3–69.3)	54.1 (53.0–55.2)	54.4 (53.3–55.5)	15.9 (15.1–16.8)	16.0 (15.2–16.8)
Virginia	6,587	84.3 (83.0–85.6)	83.8 (82.4–85.1)	72.2 (70.6–73.7)	70.9 (69.3–72.5)	69.8 (68.2–71.3)	68.9 (67.3–70.5)	15.0 (13.8–16.2)	15.1 (13.9–16.4)
Wisconsin	4,777	89.6 (88.2–90.9)	89.1 (87.5–90.5)	78.1 (76.3–79.8)	76.6 (74.7–78.4)	65.4 (63.5–67.3)	64.5 (62.5–66.5)	12.5 (11.2–13.9)	12.6 (11.2–14.1)
Wyoming	3,626	81.6 (79.3–83.7)	81.1 (78.6–83.3)	65.1 (62.5–67.7)	64.0 (61.3–66.7)	54.3 (51.7–56.9)	53.2 (50.4–55.9)	13.5 (11.7–15.5)	13.8 (11.9–16.0)
*Median*		*82.7*	*81.9*	*71.6*	*70.3*	*64.6*	*63.1*	*16.2*	*16.8*
*Range*		*71.0–89.6*	*70.8–89.1*	*62.6–85.3*	*61.8–83.9*	*52.9–71.8*	*52.1–71.1*	*11.7–22.7*	*11.9–23.1*
***Overall median***		***84.8***	***84.2***	***72.4***	***71.2***	***65.4***	***64.5***	***15.1***	***15.3***
***Overall range***		***71.0–94.7***	***70.8–94.5***	***58.5–87.5***	***57.2–86.6***	***52.9–76.7***	***52.1–75.5***	***7.9–22.7***	***8.0–23.1***

**Abbreviations:** CI = confidence interval; n = sample size; N = weighted population.

* Age standardized to the 2000 projected population for the United States.

^†^ Unweighted sample size.

^§^ A person was defined as insured if he or she had any kind of health care coverage, including health insurance, prepaid plans such as health maintenance organizations, government plans such as Medicare, or Indian Health Service at the time of interview (overall: n = 297,734, N = 197,368,555; Medicaid expansion states: n = 150,079, N = 105,640,775; Medicaid nonexpansion states: n = 147,655, N = 91,727,780).

^¶^ Weighted percentage.

** States that expanded Medicaid as of January 1, 2014 (n = 26): Arizona, Arkansas, California, Colorado, Connecticut, Delaware, District of Columbia, Hawaii, Illinois, Iowa, Kentucky, Maryland, Massachusetts, Michigan, Minnesota, Nevada, New Jersey, New Mexico, New York, North Dakota, Ohio, Oregon, Rhode Island, Vermont, Washington, and West Virginia.

^††^ States that did not expand Medicaid as of January 1, 2014 (n = 25): Alabama, Alaska, Florida, Georgia, Idaho, Indiana, Kansas, Louisiana, Maine, Mississippi, Missouri, Montana, Nebraska, New Hampshire, North Carolina, Oklahoma, Pennsylvania, South Carolina, South Dakota, Tennessee, Texas, Utah, Virginia, Wisconsin, and Wyoming.

#### Usual Source of Health Care

**FIGURE 4:**
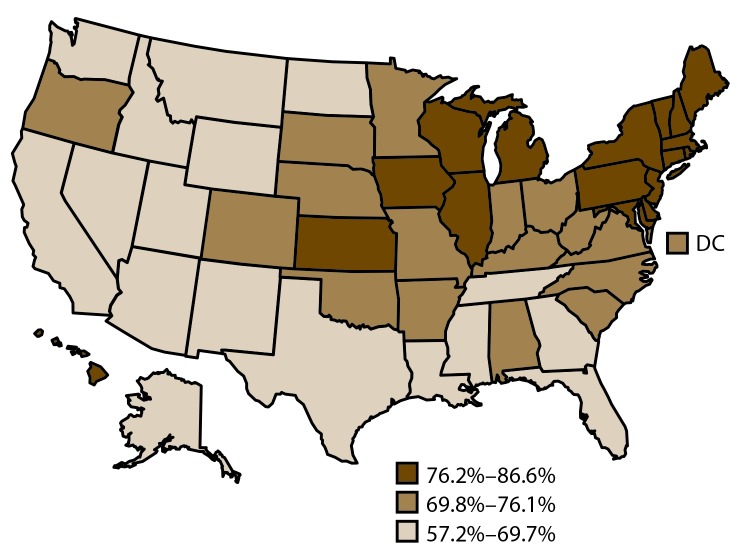
Age-standardized* prevalence estimates of usual source of health care^†^ among adults aged 18–64 years — Behavioral Risk Factor Surveillance System, United States,^§^ 2014 **Abbreviation:** DC = District of Columbia. * Age standardized to the 2000 projected population for the United States. ^†^ Survey question used to assess usual source of health care: Do you have one person you think of as your personal doctor or health care provider? ^§^ States are divided into tertiles.

Among adults aged 18–64 years, the median estimated prevalence of having a usual source of health care was 71.2%. The range was 57.2% in Nevada to 86.6% in Massachusetts (Table 1) (Figure 4).

#### Routine Checkup within the Preceding 12 Months

**FIGURE 5:**
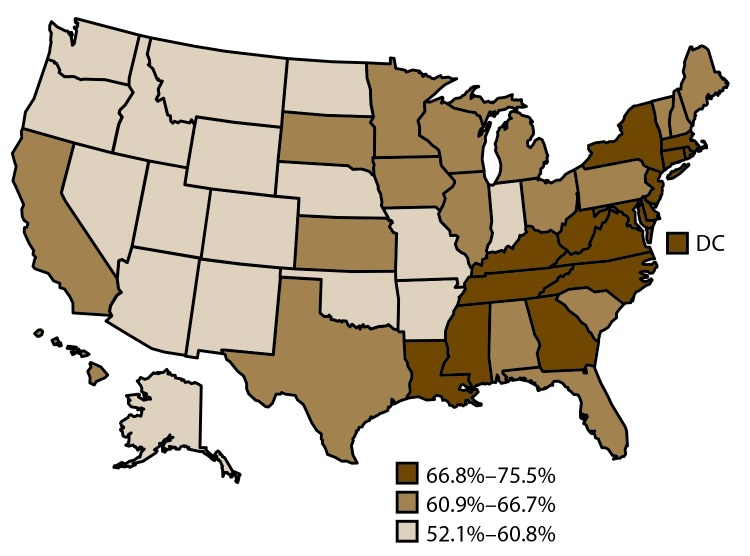
Age-standardized* prevalence estimates of routine checkup^†^ within preceding 12 months among adults aged 18–64 years — Behavioral Risk Factor Surveillance System, United States,^§^ 2014 **Abbreviation:** DC = District of Columbia. * Age standardized to the 2000 projected population for the United States. ^†^ Survey question used to assess routine checkup within preceding 12 months: About how long has it been since you last visited a doctor for a routine checkup? A routine checkup is a general physical exam, not an exam for a specific injury, illness, or condition. ^§^ States are divided into tertiles.

Among adults aged 18–64 years, the median estimated prevalence of receiving a routine checkup within the preceding 12 months was 64.5%. The range was 52.1% in Idaho to 75.5% in Rhode Island (Table 1) (Figure 5).

#### Unmet Health Care Need Because of Cost

**FIGURE 6:**
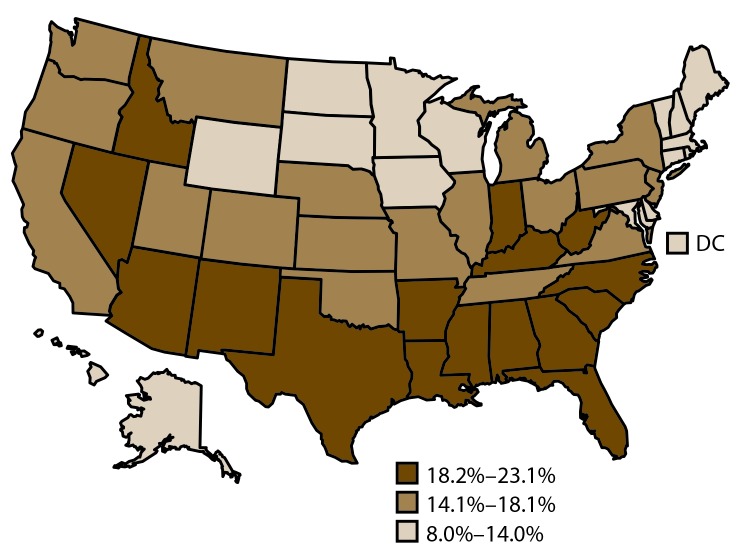
Age-standardized* prevalence estimates of cost barrier to health care need during preceding 12 months† among adults aged 18–64 years — Behavioral Risk Factor Surveillance System, United States,^§^ 2014 **Abbreviation:** DC = District of Columbia. * Age standardized to the 2000 projected population for the United States. ^†^ Survey question used to assess cost barrier to health care need during preceding 12 months: Was there a time in the past 12 months when you needed to see a doctor but could not because of cost? ^§^ States are divided into tertiles.

**FIGURE 7:**
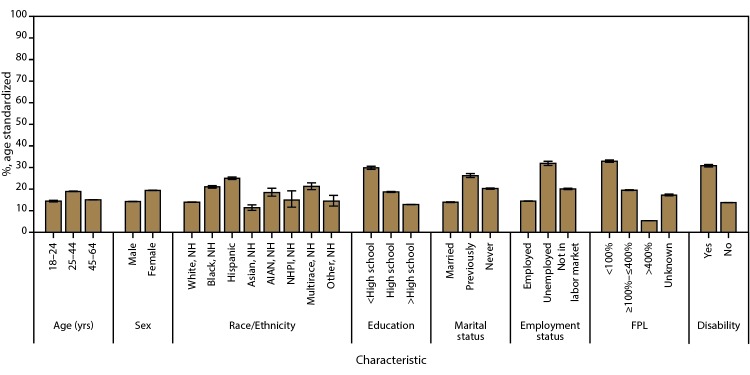
Age-standardized* prevalence estimates of cost barrier to health care need during preceding 12 months among adults aged 18–64 years, by sociodemographic characteristics — Behavioral Risk Factor Surveillance System, United States, 2014 **Abbreviations**: AIAN = American Indian or Alaskan Native; FPL = federal poverty level; NH = non-Hispanic; NHPI = Native Hawaiian or other Pacific Islander. * Age standardized to the 2000 projected U.S. population, except for age groups. Bars represent 95% confidence intervals.

Among adults aged 18–64 years, the median estimated prevalence of unmet health care need because of cost during the preceding 12 months was 15.3% and ranged from 8.0% in North Dakota to 23.1% in Mississippi (Table 1) (Figure 6). The estimated prevalence of unmet health care need because of cost was higher for adults aged 25–44 years (18.9%) than for adults aged 18–24 years (14.4%) and 45–64 years (15.0%) (Figure 7). The estimated prevalence of unmet health care need because of cost was higher among women (19.4%) than men (14.2%). By race/ethnicity, the estimated prevalence of adults reporting unmet health care need because of cost was as follows: 11.3% (non-Hispanic Asian), 13.9% (non-Hispanic white), 14.4% (non-Hispanic other race), 14.9% (non-Hispanic NHPI), 18.4% (non-Hispanic AIAN), 21.0% (non-Hispanic black), 21.2% (non-Hispanic multiple race), and 25.0% (Hispanic). The estimated prevalence of unmet health care need because of cost decreased with increasing level of educational attainment: 29.8% of adults with less than high school education, 18.7% of adults with a high school education, and 12.8% of adults with greater than high school education had unmet health care need because of cost. By marital status, 13.9% of married adults had unmet health care need because of cost, whereas 20.2% of never married adults and 26.2% of previously married (divorced, separated, widowed) adults had unmet health care need because of cost. By employment status, 14.4% of employed adults had unmet health care need because of cost, whereas 20.0% of adults not in the labor market and 31.9% of unemployed adults reported a cost barrier to health care need. Adults with household income <100% of FPL were the most likely to have unmet health care need because of cost (32.9%), compared with adults in other income groups (19.5% and 5.3% among adults with household income ≥100%–≤400% of FPL and >400% of FPL, respectively). The estimated prevalence of unmet health care need because of cost was higher for adults with disabilities (30.8%) than for adults without disabilities (13.7%). Crude state-specific estimates of cost barrier to health care need during the preceding 12 months by sociodemographic characteristics are available (supplemental Table S2 https://stacks.cdc.gov/view/cdc/43255).

### Health Care Access by State Medicaid Expansion Status, Expanded Geographic Region, and FPL

Under provisions of ACA, states have the option to expand Medicaid coverage to persons in households with incomes ≤138% of FPL (Figure 1). In Medicaid expansion states, the median estimated prevalence of working-aged adults who were insured at the time of interview was 87.0% and ranged from 78.8% in Nevada to 94.5% in Massachusetts (Table 1). In nonexpansion states, the median estimated prevalence of insurance coverage at the time of interview was 81.9% and ranged from 70.8% in Texas to 89.1% in Wisconsin (Table 1). In the nine expanded geographic regions, the estimated prevalence of insurance coverage at time of interview ranged from 73.3% in the West South Central region to 91.0% in the New England region (Table 2). The estimated prevalence of adults who were insured at the time of interview increased with increasing level of income (% of FPL) in Medicaid expansion states and nonexpansion states and in each expanded geographic region. For example, in the West South Central region, the estimated prevalence of insurance coverage at the time of interview was 47.0% among adults with household income <100% of FPL, 72.9% among adults with household income ≥100%–≤400% of FPL, and 94.8% among adults with household income >400% of FPL; in the New England region, these percentages were 79.4%, 88.0%, and 97.9%, respectively (Table 3).

**TABLE 2:** Crude and age-standardized* prevalence estimates of health insurance coverage, usual source of health care, routine checkup within the preceding 12 months, and cost barrier to health care need during the preceding 12 months among adults aged 18–64 years, by state Medicaid expansion status and expanded geographic regions — Behavioral Risk Factor Surveillance System, United States, 2014

Category	Insured at the time of interview^†^		Usual source of health care		Routine checkup within preceding 12 mos		Cost barrier to health care need during preceding 12 mos	
	Crude	Age standardized	Crude	Age standardized	Crude	Age standardized	Crude	Age standardized
	%^§^ (95% CI)	% (95% CI)	% (95% CI)	% (95% CI)	% (95% CI)	% (95% CI)	% (95% CI)	% (95% CI)
**State Medicaid expansion status**								
Medicaid expansion states^¶^	85.8 (85.4–86.2)	85.3 (84.8–85.7)	75.0 (74.5–75.4)	73.8 (73.4–74.3)	65.8 (65.4–66.3)	65.0 (64.5–65.5)	15.0 (14.6–15.4)	15.3 (14.9–15.7)
Medicaid nonexpansion states**	79.7 (79.3–80.1)	79.1 (78.7–79.5)	70.9 (70.5–71.4)	69.7 (69.3–70.2)	65.7 (65.3–66.2)	64.9 (64.4–65.3)	18.3 (17.9–18.7)	18.5 (18.1–18.9)
**Expanded geographic regions^††^**								
New England	91.5 (90.9–92.0)	91.0 (90.5–91.6)	84.9 (84.2–85.5)	83.6 (82.8–84.3)	71.4 (70.6–72.2)	70.2 (69.3–71.0)	11.3 (10.8–11.9)	11.6 (11.0–12.3)
Middle Atlantic	86.5 (85.8–87.1)	85.7 (85.0–86.5)	79.4 (78.6–80.2)	78.2 (77.3–79.0)	70.8 (69.9–71.6)	69.7 (68.8–70.6)	14.8 (14.1–15.4)	15.2 (14.5–15.9)
East North Central	86.5 (85.8–87.2)	85.9 (85.1–86.6)	77.9 (77.1–78.7)	76.7 (75.9–77.5)	65.4 (64.5–66.2)	64.3 (63.4–65.2)	15.2 (14.6–15.9)	15.5 (14.8–16.2)
West North Central	87.0 (86.5–87.6)	86.5 (85.9–87.1)	74.5 (73.8–75.1)	73.3 (72.6–74.0)	63.6 (62.9–64.4)	62.6 (61.8–63.4)	12.9 (12.4–13.5)	13.3 (12.8–13.9)
South Atlantic	79.0 (78.3–79.7)	78.3 (77.5–79.1)	70.3 (69.5–71.1)	68.8 (68.0–69.7)	68.4 (67.6–69.2)	67.4 (66.6–68.2)	20.0 (19.3–20.7)	20.3 (19.6–21.0)
East South Central	82.9 (82.0–83.8)	82.3 (81.3–83.3)	71.9 (70.8–73.0)	70.8 (69.6–71.9)	68.8 (67.7–69.9)	68.0 (66.8–69.1)	19.5 (18.6–20.4)	19.6 (18.7–20.6)
West South Central	73.7 (72.6–74.7)	73.3 (72.2–74.4)	65.1 (63.9–66.2)	64.4 (63.2–65.5)	63.9 (62.8–65.0)	63.4 (62.3–64.5)	19.8 (18.9–20.7)	19.9 (19.0–20.8)
Mountain	82.5 (81.9–83.1)	82.1 (81.4–82.7)	66.8 (66.1–67.5)	66.0 (65.3–66.7)	57.5 (56.7–58.2)	56.8 (56.1–57.6)	17.2 (16.6–17.8)	17.4 (16.8–18.0)
Pacific	83.6 (82.7–84.5)	83.2 (82.2–84.1)	70.8 (69.8–71.9)	69.9 (68.8–71.0)	61.9 (60.8–63.1)	61.3 (60.2–62.5)	15.0 (14.2–15.9)	15.2 (14.4–16.1)
**Overall**	**83.0 (82.7–83.3)**	**82.4 (82.1–82.7)**	**73.1 (72.8–73.4)**	**71.9 (71.6–72.3)**	**65.8 (65.5–66.1)**	**64.9 (64.6–65.3)**	**16.5 (16.3–16.8)**	**16.8 (16.5–17.1)**

**Abbreviations:** CI = confidence interval; n = sample size; N = weighted population.

* Age standardized to the 2000 projected population for the United States.

^†^ A person was defined as insured if he or she had any kind of health care coverage, including health insurance, prepaid plans such as health maintenance organizations, government plans such as Medicare, or Indian Health Service at the time of interview (overall: n = 297,734, N = 197,368,555; Medicaid expansion states: n = 150,079, N = 105,640,775; Medicaid nonexpansion states: n = 147,655, N = 91,727,780).

^§^ Weighted percentage.

^¶^ States that expanded Medicaid as of January 1, 2014 (n = 26): Arizona, Arkansas, California, Colorado, Connecticut, Delaware, District of Columbia, Hawaii, Illinois, Iowa, Kentucky, Maryland, Massachusetts, Michigan, Minnesota, Nevada, New Jersey, New Mexico, New York, North Dakota, Ohio, Oregon, Rhode Island, Vermont, Washington, and West Virginia.

** States that did not expand Medicaid as of January 1, 2014 (n = 25): Alabama, Alaska, Florida, Georgia, Idaho, Indiana, Kansas, Louisiana, Maine, Mississippi, Missouri, Montana, Nebraska, New Hampshire, North Carolina, Oklahoma, Pennsylvania, South Carolina, South Dakota, Tennessee, Texas, Utah, Virginia, Wisconsin, and Wyoming.

^††^ Expanded geographic regions are the four census regions subdivided into nine regions. For this report, the nine census regions were modified by moving Delaware, District of Columbia, and Maryland into the Middle Atlantic region*. New England:* Connecticut, Maine, Massachusetts, New Hampshire, Rhode Island, and Vermont. *Middle Atlantic:* Delaware, District of Columbia, Maryland, New Jersey, New York, and Pennsylvania. *East North Central*: Illinois, Indiana, Michigan, Ohio, and Wisconsin. *West North Central:* Iowa, Kansas, Minnesota, Missouri, Nebraska, North Dakota, and South Dakota. *South Atlantic:* Florida, Georgia, North Carolina, South Carolina, Virginia, and West Virginia. *East South Central:* Alabama, Kentucky, Mississippi, and Tennessee. *West South Central:* Arkansas, Louisiana, Oklahoma, and Texas. *Mountain:* Arizona, Colorado, Idaho, Montana, Nevada, New Mexico, Utah, and Wyoming. *Pacific:* Alaska, California, Hawaii, Oregon, and Washington.

**TABLE 3:** Crude and age-standardized* prevalence estimates of health insurance coverage, usual source of health care, routine checkup within the preceding 12 months, and cost barrier to health care need during the preceding 12 months among adults aged 18–64 years, by federal poverty level, state Medicaid expansion status, and expanded geographic regions — Behavioral Risk Factor Surveillance System, United States, 2014

Category	Insured at time of interview^†^		Usual source of health care		Routine checkup within preceding 12 mos		Cost barrier to health care need during preceding 12 mos	
	Crude	Age standardized	Crude	Age standardized	Crude	Age standardized	Crude	Age standardized
	%^§^ (95% CI)	% (95% CI)	% (95% CI)	% (95% CI)	% (95% CI)	% (95% CI)	% (95% CI)	% (95% CI)
**State Medicaid expansion status**								
**Medicaid expansion states^¶^**								
<100 of FPL	71.4 (70.1–72.7)	71.4 (70.1–72.7)	63.0 (61.6–64.4)	63.9 (62.5–65.2)	60.8 (59.4–62.2)	61.2 (59.8–62.5)	27.6 (26.4–28.9)	28.1 (26.8–29.3)
≥100%–≤400% of FPL	83.8 (83.1–84.4)	83.3 (82.6–84.1)	72.5 (71.7–73.3)	71.3 (70.4–72.1)	63.9 (63.0–64.8)	63.0 (62.1–63.9)	18.7 (18.0–19.4)	18.8 (18.1–19.5)
>400% of FPL	96.4 (96.0–96.7)	96.1 (95.6–96.5)	85.0 (84.4–85.6)	82.9 (82.1–83.6)	69.9 (69.1–70.6)	68.5 (67.7–69.4)	4.8 ( 4.5–5.2)	5.0 ( 4.6–5.4)
Unknown	83.6 (82.5–84.5)	83.2 (82.1–84.2)	72.2 (71.0–73.3)	72.6 (71.4–73.7)	66.6 (65.4–67.7)	66.1 (64.9–67.3)	15.3 (14.4–16.2)	16.1 (15.2–17.1)
Medicaid nonexpansion states^**^								
<100 of FPL	55.8 (54.5–57.1)	55.9 (54.6–57.1)	56.9 (55.6–58.1)	57.7 (56.5–59.0)	58.2 (57.0–59.5)	58.5 (57.3–59.8)	38.1 (36.9–39.4)	38.5 (37.3–39.8)
≥100%–≤400% of FPL	78.8 (78.1–79.5)	78.3 (77.6–79.0)	69.8 (69.1–70.6)	68.7 (67.9–69.5)	64.5 (63.8–65.3)	63.7 (63.0–64.5)	20.2 (19.5–20.8)	20.2 (19.5–20.8)
>400% of FPL	95.7 (95.3–96.1)	95.4 (94.9–95.8)	82.7 (82.0–83.3)	80.0 (79.2–80.8)	71.7 (70.9–72.4)	70.1 (69.2–70.9)	5.5 ( 5.1–5.9)	5.6 ( 5.2–6.1)
Unknown	76.2 (75.0–77.4)	75.3 (73.9–76.6)	65.6 (64.3–66.9)	66.2 (64.8–67.6)	64.9 (63.5–66.1)	64.9 (63.5–66.3)	17.6 (16.6–18.7)	18.9 (17.7–20.1)
**Expanded geographic regions^††^**								
**New England**								
<100 of FPL	79.5 (77.0–81.7)	79.4 (77.0–81.6)	74.2 (71.5–76.7)	74.8 (72.3–77.2)	67.4 (64.6–70.2)	68.0 (65.3–70.6)	22.3 (20.0–24.9)	22.6 (20.3–25.0)
≥100%–≤400% of FPL	88.4 (87.4–89.3)	88.0 (86.9–89.0)	84.4 (83.2–85.5)	82.7 (81.3–84.0)	69.6 (68.1–71.0)	67.8 (66.2–69.4)	15.6 (14.5–16.8)	15.7 (14.5–17.0)
>400% of FPL	98.1 (97.6–98.4)	97.9 (97.2–98.4)	92.0 (91.2–92.7)	90.3 (89.2–91.3)	74.0 (72.8–75.2)	72.3 (70.8–73.8)	4.1 ( 3.6–4.7)	4.1 ( 3.5–4.7)
Unknown	89.8 (88.5–91.0)	89.6 (88.3–90.8)	79.4 (77.7–81.0)	80.0 (78.4–81.6)	71.2 (69.5–73.0)	71.2 (69.5–72.9)	13.0 (11.7–14.4)	13.6 (12.3–15.0)
**Middle Atlantic**								
<100 of FPL	71.6 (69.2–73.9)	71.8 (69.4–74.0)	69.8 (67.3–72.2)	70.2 (67.8–72.5)	67.3 (64.8–69.8)	67.6 (65.1–70.0)	30.1 (27.8–32.5)	30.0 (27.7–32.4)
≥100%–≤400% of FPL	83.3 (81.9–84.6)	82.7 (81.2–84.0)	77.5 (76.0–78.9)	76.3 (74.7–77.7)	69.5 (67.9–71.0)	68.6 (67.0–70.2)	19.3 (18.0–20.7)	19.5 (18.2–20.9)
>400% of FPL	96.7 (96.1–97.2)	96.5 (95.8–97.1)	87.0 (86.0–87.9)	85.2 (84.0–86.4)	73.1 (71.9–74.3)	72.2 (70.8–73.6)	5.0 ( 4.4–5.6)	5.2 ( 4.5–5.9)
Unknown	83.2 (81.1–85.2)	82.1 (79.7–84.3)	74.7 (72.4–76.9)	74.3 (71.8–76.6)	71.1 (68.8–73.3)	69.4 (66.9–71.8)	13.7 (12.0–15.5)	14.9 (13.0–17.0)
**East North Central**								
<100 of FPL	70.6 (67.9–73.1)	70.4 (67.8–72.9)	66.6 (63.9–69.2)	67.8 (65.2–70.3)	61.3 (58.5–64.0)	61.8 (59.1–64.4)	29.7 (27.2–32.3)	30.7 (28.2–33.3)
≥100%–≤400% of FPL	84.8 (83.5–85.9)	84.2 (82.9–85.5)	76.5 (75.1–77.8)	75.1 (73.7–76.6)	63.4 (61.9–64.9)	62.4 (60.8–63.9)	18.2 (17.0–19.4)	18.2 (17.0–19.5)
>400% of FPL	97.1 (96.4–97.7)	96.8 (95.9–97.6)	86.6 (85.5–87.6)	84.5 (83.1–85.8)	69.6 (68.2–70.9)	67.9 (66.3–69.5)	4.7 ( 4.1–5.4)	4.8 ( 4.1–5.5)
Unknown	83.7 (82.2–85.1)	83.3 (81.7–84.7)	74.6 (72.9–76.3)	74.8 (73.1–76.5)	64.8 (63.1–66.5)	64.2 (62.4–65.9)	16.8 (15.4–18.2)	17.6 (16.1–19.1)
**West North Central**								
<100 of FPL	67.7 (65.4–70.0)	67.5 (65.3–69.7)	64.3 (62.0–66.6)	66.1 (63.9–68.2)	56.0 (53.7–58.4)	57.3 (55.0–59.5)	30.7 (28.5–32.9)	31.9 (29.8–34.1)
≥100%–≤400% of FPL	85.6 (84.6–86.5)	85.3 (84.3–86.2)	71.8 (70.7–72.9)	70.9 (69.7–72.1)	60.5 (59.2–61.7)	59.7 (58.4–60.9)	15.4 (14.5–16.4)	15.5 (14.6–16.5)
>400% of FPL	97.2 (96.6–97.7)	96.7 (96.0–97.4)	82.4 (81.4–83.3)	79.7 (78.4–80.9)	69.8 (68.8–70.9)	68.4 (67.1–69.7)	3.9 ( 3.4–4.4)	3.9 ( 3.4–4.6)
Unknown	81.8 (79.8–83.6)	81.4 (79.3–83.3)	70.3 (68.1–72.4)	70.6 (68.4–72.8)	62.6 (60.4–64.8)	61.9 (59.6–64.2)	13.9 (12.3–15.7)	16.0 (14.1–18.1)
**South Atlantic**								
<100 of FPL	55.9 (53.8–58.1)	55.9 (53.8–58.0)	55.8 (53.6–58.0)	56.5 (54.4–58.6)	59.4 (57.3–61.5)	59.5 (57.4–61.6)	40.5 (38.4–42.6)	40.9 (38.9–43.0)
≥100%–≤400% of FPL	77.9 (76.7–79.1)	77.4 (76.1–78.6)	69.3 (67.9–70.6)	67.8 (66.4–69.1)	67.8 (66.5–69.1)	66.9 (65.5–68.2)	21.5 (20.5–22.7)	21.6 (20.5–22.8)
>400% of FPL	95.1 (94.3–95.8)	94.6 (93.6–95.4)	82.6 (81.4–83.7)	79.6 (78.2–81.0)	74.6 (73.3–75.8)	72.7 (71.3–74.2)	6.1 ( 5.5–6.8)	6.3 ( 5.6–7.1)
Unknown	76.6 (74.5–78.6)	75.0 (72.7–77.2)	65.7 (63.4–67.9)	65.9 (63.5–68.2)	68.0 (65.9–70.1)	67.6 (65.3–69.9)	19.9 (18.0–21.9)	21.1 (19.0–23.4)
**East South Central**								
<100 of FPL	67.5 (64.9–70.0)	67.7 (65.1–70.1)	64.3 (61.5–67.0)	64.7 (62.1–67.3)	64.5 (62.3–66.6)	63.7 (61.0–66.3)	37.5 (35.0–40.1)	37.4 (34.9–40.0)
≥100%–≤400% of FPL	82.8 (81.2–84.3)	82.2 (80.5–83.7)	71.3 (69.5–72.9)	69.8 (68.0–71.6)	68.7 (66.7–70.7)	67.3 (65.4–69.1)	20.4 (19.0–21.9)	20.3 (18.8–21.8)
>400% of FPL	96.7 (95.8–97.4)	96.3 (95.2–97.1)	81.4 (79.4–83.2)	77.5 (75.1–79.7)	73.6 (71.6–75.6)	71.1 (68.6–73.5)	5.6 ( 4.6–6.8)	6.0 ( 4.9–7.5)
Unknown	79.6 (76.8–82.2)	79.9 (77.0–82.5)	68.0 (64.9–70.8)	69.6 (66.6–72.4)	68.7 (65.8–71.5)	69.1 (66.1–72.0)	18.0 (15.9–20.4)	18.5 (16.3–21.0)
**West South Central**								
<100 of FPL	46.8 (44.2–49.4)	47.0 (44.4–49.6)	50.5 (47.8–53.1)	51.2 (48.6–53.8)	56.5 (53.9–59.1)	56.7 (54.1–59.3)	38.9 (36.4–41.5)	39.3 (36.7–41.8)
≥100%–≤400% of FPL	73.1 (71.3–74.8)	72.9 (71.1–74.7)	63.9 (62.0–65.8)	63.3 (61.4–65.2)	62.5 (60.6–64.4)	62.1 (60.2–64.0)	21.5 (20.0–23.0)	21.4 (19.9–23.0)
>400% of FPL	94.9 (93.9–95.7)	94.8 (93.8–95.6)	80.6 (78.9–82.3)	78.4 (76.3–80.3)	72.5 (70.6–74.3)	71.4 (69.2–73.4)	5.2 ( 4.3–6.2)	5.1 ( 4.3–6.1)
Unknown	70.1 (67.3–72.8)	69.5 (66.5–72.3)	57.6 (54.6–60.5)	59.0 (56.1–61.9)	60.3 (57.2–63.3)	61.4 (58.3–64.4)	18.6 (16.5–20.9)	20.2 (17.9–22.8)
**Mountain**								
<100 of FPL	65.2 (63.3–67.1)	65.5 (63.6–67.4)	53.8 (51.8–55.8)	55.3 (53.4–57.3)	50.8 (48.8–52.8)	51.6 (49.6–53.6)	30.3 (28.5–32.1)	31.1 (29.3–32.9)
≥100%–≤400% of FPL	80.5 (79.4–81.5)	80.4 (79.2–81.4)	64.3 (63.0–65.5)	63.8 (62.5–65.0)	54.1 (52.9–55.4)	53.8 (52.6–55.0)	20.5 (19.5–21.5)	20.5 (19.5–21.5)
>400% of FPL	95.9 (95.4–96.4)	95.6 (95.0–96.2)	78.3 (77.3–79.3)	76.0 (74.7–77.3)	64.8 (63.7–65.8)	62.6 (61.3–64.0)	6.1 ( 5.6–6.7)	6.3 ( 5.7–7.1)
Unknown	78.3 (76.4–80.2)	77.5 (75.4–79.4)	63.0 (60.9–65.1)	63.1 (60.9–65.2)	57.5 (55.3–59.6)	56.8 (54.6–59.0)	17.8 (16.1–19.6)	19.3 (17.4–21.4)
**Pacific**								
<100 of FPL	70.1 (67.5–72.5)	69.8 (67.2–72.3)	58.8 (56.2–61.4)	59.4 (56.8–62.0)	57.7 (55.0–60.3)	57.8 (55.1–60.4)	25.9 (23.7–28.2)	26.3 (24.0–28.7)
≥100%–≤400% of FPL	82.3 (80.7–83.8)	82.0 (80.4–83.6)	67.6 (65.6–69.5)	66.7 (64.7–68.6)	60.4 (58.3–62.4)	59.8 (57.7–61.8)	18.4 (16.8–20.0)	18.4 (16.9–20.0)
>400% of FPL	95.1 (94.1–95.9)	94.7 (93.5–95.6)	83.3 (81.8–84.6)	80.8 (79.1–82.5)	65.1 (63.3–66.9)	63.7 (61.7–65.7)	5.0 ( 4.2–5.9)	5.3 ( 4.3–6.5)
Unknown	82.0 (79.1–84.5)	82.2 (79.4–84.7)	68.9 (65.7–72.0)	69.8 (66.6–72.8)	64.5 (61.2–67.8)	64.8 (61.5–68.0)	13.6 (11.5–16.0)	14.0 (11.8–16.5)
**Overall**								
<100 of FPL	64.2 (63.2–65.1)	64.2 (63.2–65.1)	60.2 (59.2–61.1)	61.0 (60.1–61.9)	59.6 (58.7–60.6)	59.9 (59.0–60.9)	32.5 (31.6–33.4)	32.9 (32.1–33.8)
≥100%–≤400% of FPL	81.2 (80.8–81.7)	80.8 (80.3–81.3)	71.1 (70.6–71.7)	70.0 (69.4–70.5)	64.2 (63.6–64.8)	63.4 (62.8–64.0)	19.4 (19.0–19.9)	19.5 (19.0–20.0)
>400% of FPL	96.1 (95.8–96.3)	95.8 (95.5–96.1)	84.0 (83.5–84.4)	81.6 (81.1–82.2)	70.7 (70.2–71.2)	69.2 (68.6–69.8)	5.1 ( 4.9–5.4)	5.3 ( 5.0–5.6)
Unknown	80.5 (79.7–81.2)	80.0 (79.1–80.8)	69.4 (68.5–70.3)	70.0 (69.1–70.8)	65.8 (65.0–66.7)	65.6 (64.7–66.5)	16.3 (15.6–16.9)	17.2 (16.5–18.0)

**Abbreviations:** CI = confidence interval; FPL = federal poverty level; n = sample size; N = weighted population.

* Age-standardized to the 2000 projected population for the United States.

^†^ A person was defined as insured if he or she had any kind of health care coverage, including health insurance, prepaid plans such as health maintenance organizations, government plans such as Medicare, or Indian Health Service at the time of interview (overall: n = 297,734, N = 197,368,555; Medicaid expansion states: n = 150,079, N = 105,640,775; Medicaid nonexpansion states: n = 147,655, N = 91,727,780).

^§^ Weighted percentage.

^¶^ States that expanded Medicaid as of January 1, 2014 (n = 26): Arizona, Arkansas, California, Colorado, Connecticut, Delaware, District of Columbia, Hawaii, Illinois, Iowa, Kentucky, Maryland, Massachusetts, Michigan, Minnesota, Nevada, New Jersey, New Mexico, New York, North Dakota, Ohio, Oregon, Rhode Island, Vermont, Washington, and West Virginia.

** States that did not expand Medicaid as of January 1, 2014 (n = 25): Alabama, Alaska, Florida, Georgia, Idaho, Indiana, Kansas, Louisiana, Maine, Mississippi, Missouri, Montana, Nebraska, New Hampshire, North Carolina, Oklahoma, Pennsylvania, South Carolina, South Dakota, Tennessee, Texas, Utah, Virginia, Wisconsin, and Wyoming.

^††^ Expanded geographic regions are the four census regions subdivided into nine regions. For this report, the nine census regions were modified by moving Delaware, District of Columbia, and Maryland into the Middle Atlantic region. *New England:* Connecticut, Maine, Massachusetts, New Hampshire, Rhode Island, and Vermont. *Middle Atlantic:* Delaware, District of Columbia, Maryland, New Jersey, New York, and Pennsylvania. *East North Central:* Illinois, Indiana, Michigan, Ohio, and Wisconsin. *West North Central*: Iowa, Kansas, Minnesota, Missouri, Nebraska, North Dakota, and South Dakota. *South Atlantic:* Florida, Georgia, North Carolina, South Carolina, Virginia, and West Virginia*. East South Central:* Alabama, Kentucky, Mississippi, and Tennessee. *West South Central*: Arkansas, Louisiana, Oklahoma, and Texas. *Mountain:* Arizona, Colorado, Idaho, Montana, Nevada, New Mexico, Utah, and Wyoming. *Pacific:* Alaska, California, Hawaii, Oregon, and Washington.

### Other Health Care Access Measures

In Medicaid expansion states, the median estimated prevalence of working-aged adults who had a usual source of health care was 74.6% and ranged from 57.2% in Nevada to 86.6% in Massachusetts (Table 1). In nonexpansion states, the median estimated prevalence of adults who had a usual source of health care was 70.3% and ranged from 61.8% in Alaska to 83.9% in Maine. The estimated prevalence of adults who had a routine checkup within the preceding 12 months varied by approximately 19 percentage points in Medicaid expansion states (median: 64.8%, range: 56.0% in Oregon to 75.5% in Rhode Island) and nonexpansion states (median: 63.1%, range: 52.1% in Idaho to 71.1% in Louisiana), respectively. The estimated prevalence of adults who had unmet health care need because of cost varied by 13.9 percentage points in Medicaid expansion states (median: 14.7%, range: 8.0% in North Dakota to 21.9% in Arkansas) and 11.2 percentage points in nonexpansion states (median: 16.8%, range: 11.9% in South Dakota to 23.1% in Mississippi), respectively (Table 1).

In the nine expanded geographic regions, the estimated prevalence of adults who had a usual source of health care ranged from 64.4% in the West South Central region to 83.6% in the New England region (Table 2). The estimated prevalence of adults who had a routine checkup within the preceding 12 months ranged from 56.8% in the Mountain region to 70.2% in the New England region. The estimated prevalence of adults who had unmet health care need because of cost ranged from 11.6% in the New England region to 20.3% in the South Atlantic region.

By FPL, the estimated prevalence of adults who had a usual source of health care and no unmet health care need because of cost increased with increasing level of income (% of FPL) in Medicaid expansion states and nonexpansion states and in each expanded geographic region, although the magnitude of these estimates varied across the examined categories. For example, in Medicaid expansion states, the estimated prevalence of a usual source of health care was 63.9% among adults with household income <100% of FPL, 71.3% among adults with household income ≥100%–≤400% of FPL, and 82.9% among adults with household income >400% of FPL; in nonexpansion states, these percentages were 57.7%, 68.7%, and 80.0%, respectively (Table 3). In another example, the estimated prevalence of unmet health care need because of cost among adults with household income <100% of FPL ranged from 22.6% in the New England region to 40.9% in the South Atlantic region. Among adults with household income ≥100%–≤400% of FPL, estimates ranged from 15.5% in the West North Central region to 21.6% in the South Atlantic region. Among adults with household income >400% of FPL, estimates ranged from 3.9% in the West North Central region to 6.3% in both the Mountain region and the South Atlantic region (Table 3). In contrast, the estimated prevalence of a routine checkup within the preceding 12 months had less variability by FPL category (except for increasing estimates with increasing level of income in the South Atlantic region and the West South Central region and in nonexpansion states) (Table 3).

### Selected Clinical Preventive Services

#### Colorectal Cancer Screening for Adults Aged 50–64 Years

Among adults aged 50–64 years, the median estimated prevalence of having received colorectal cancer screening as recommended by USPSTF was 59.1% and ranged from 49.2% in Nevada to 72.4% in Massachusetts. Crude and age-standardized state-specific estimates for CPS use (including colorectal cancer screening) are available (supplemental Table S3 https://stacks.cdc.gov/view/cdc/43255). The estimated prevalence was 61.1% in Medicaid expansion states and 58.6% in nonexpansion states (Table 4). Among expanded geographic regions, the estimated prevalence ranged from 54.1% in the West South Central region to 70.5% in the New England region (Table 4).

**TABLE 4:** Crude and age-standardized* prevalence estimates of clinical preventive services use among adults, by state Medicaid expansion status and expanded geographic regions — Behavioral Risk Factor Surveillance System, United States, 2014

Category	Colorectal cancer screening^†^		HIV test^§^		Influenza vaccination^¶^	
	Crude	Age standardized	Crude	Age standardized	Crude	Age standardized
	%** (95% CI)	% (95% CI)	% (95% CI)	% (95% CI)	% (95% CI)	% (95% CI)
State Medicaid expansion status						
Medicaid expansion states^††^	61.8 (61.1–62.6)	61.1 (60.3–61.8)	41.8 (41.3–42.3)	43.2 (42.7–43.7)	34.3 (33.8–34.8)	33.6 (33.1–34.1)
Medicaid nonexpansion states^§§^	59.5 (58.7–60.2)	58.6 (57.8–59.3)	42.0 (41.5–42.4)	43.3 (42.8–43.8)	33.4 (32.9–33.8)	32.8 (32.3–33.2)
**Expanded geographic regions^¶¶^**						
New England	71.1 (70.0–72.2)	70.5 (69.3–71.7)	41.1 (40.2–42.0)	43.4 (42.5–44.3)	39.0 (38.1–39.9)	38.2 (37.2–39.1)
Middle Atlantic	62.8 (61.4–64.1)	62.2 (60.9–63.5)	46.3 (45.3–47.2)	47.8 (46.8–48.7)	34.3 (33.4–35.2)	33.5 (32.6–34.4)
East North Central	60.8 (59.5–62.0)	60.1 (58.9–61.4)	35.5 (34.6–36.3)	37.2 (36.3–38.2)	32.8 (32.0–33.6)	32.1 (31.3–33.0)
West North Central	61.1 (60.0–62.1)	60.3 (59.2–61.4)	32.0 (31.3–32.8)	33.7 (32.9–34.4)	38.1 (37.4–38.9)	37.4 (36.6–38.1)
South Atlantic	61.2 (60.0–62.4)	60.1 (58.9–61.3)	48.5 (47.7–49.4)	50.0 (49.1–50.9)	32.0 (31.2–32.7)	31.3 (30.6–32.1)
East South Central	58.4 (56.9–60.0)	57.4 (55.8–59.0)	43.2 (42.0–44.4)	44.8 (43.6–46.0)	35.0 (33.9–36.1)	34.2 (33.1–35.3)
West South Central	54.9 (53.0–56.8)	54.1 (52.2–56.0)	40.4 (39.2–41.6)	41.1 (40.0–42.3)	34.5 (33.5–35.7)	34.1 (33.0–35.2)
Mountain	57.4 (56.3–58.4)	56.2 (55.1–57.3)	37.2 (36.4–38.0)	38.1 (37.3–38.8)	32.7 (32.0–33.4)	32.3 (31.7–33.0)
Pacific	61.0 (58.9–62.9)	60.0 (58.0–62.0)	43.8 (42.5–45.0)	44.8 (43.6–46.1)	32.6 (31.4–33.8)	32.0 (30.9–33.2)
**Overall**	**60.7 (60.2–61.2)**	**59.9 (59.4–60.4)**	**41.9 (41.5–42.2)**	**43.2 (42.9–43.6)**	**33.9 (33.5–34.2)**	**33.2 (32.9–33.5)**

**Abbreviations:** CI = confidence interval; n = sample size; N = weighted population.

* Age standardized to the 2000 projected population for the United States.

^†^ Adults aged 50–64 years who have had a fecal occult blood test (FOBT) within the past year, or sigmoidoscopy within the past 5 years and an FOBT within the past 3 years, or colonoscopy within the past 10 years (n = 133,620, N = 57,378,775).

^§^ Adults aged 18–64 years ever tested for HIV (n = 269,889, N = 174,047,283).

^¶^ Adults aged 18–64 years who received an influenza vaccination within the preceding 12 months (n = 281,684, N = 181,381,790).

** Weighted percentage.

**^††^** States that expanded Medicaid as of January 1, 2014 (n = 26): Arizona, Arkansas, California, Colorado, Connecticut, Delaware, District of Columbia, Hawaii, Illinois, Iowa, Kentucky, Maryland, Massachusetts, Michigan, Minnesota, Nevada, New Jersey, New Mexico, New York, North Dakota, Ohio, Oregon, Rhode Island, Vermont, Washington, and West Virginia.

^§§^ States that did not expand Medicaid as of January 1, 2014 (n = 25): Alabama, Alaska, Florida, Georgia, Idaho, Indiana, Kansas, Louisiana, Maine, Mississippi, Missouri, Montana, Nebraska, New Hampshire, North Carolina, Oklahoma, Pennsylvania, South Carolina, South Dakota, Tennessee, Texas, Utah, Virginia, Wisconsin, and Wyoming.

^¶¶^ Expanded geographic regions are the four census regions subdivided into nine regions. For this report, the nine census regions were modified by moving Delaware, District of Columbia, and Maryland into the Middle Atlantic region. *New England:* Connecticut, Maine, Massachusetts, New Hampshire, Rhode Island, and Vermont*. Middle Atlantic:* Delaware, District of Columbia, Maryland, New Jersey, New York, and Pennsylvania. *East North Central:* Illinois, Indiana, Michigan, Ohio, and Wisconsin. *West North Central:* Iowa, Kansas, Minnesota, Missouri, Nebraska, North Dakota, and South Dakota. *South Atlantic:* Florida, Georgia, North Carolina, South Carolina, Virginia, and West Virginia. *East South Central:* Alabama, Kentucky, Mississippi, and Tennessee. *West South Central:* Arkansas, Louisiana, Oklahoma, and Texas. *Mountain:* Arizona, Colorado, Idaho, Montana, Nevada, New Mexico, Utah, and Wyoming. *Pacific:* Alaska, California, Hawaii, Oregon, and Washington.

The estimated prevalence of colorectal cancer screening generally increased with increasing level of income (% of FPL) in Medicaid expansion states and nonexpansion states and in each expanded geographic region (except for similar estimates among adults with household income <100% of FPL and those with household income ≥100%–≤400% of FPL in the New England and Middle Atlantic regions), although the magnitude of these estimates varied across the examined categories. For example, the estimated prevalence of colorectal cancer screening among adults with household income <100% of FPL ranged from 38.7% in the West South Central region to 57.7% in the New England region. Among adults with household income ≥100%–≤400% of FPL, estimates ranged from 50.0% in the Mountain region to 64.8% in the New England region. Among adults with household income >400% of FPL, estimates ranged from 65.4% in the Mountain region to 77.2% in the New England region (Table 5).

**TABLE 5:** Crude and age-standardized* prevalence estimates of selected clinical preventive services use among adults, by federal poverty level, state Medicaid expansion status, and expanded geographic regions — Behavioral Risk Factor Surveillance System, United States, 2014

Category	Colorectal cancer screening^†^		HIV test^§^		Influenza vaccination^¶^	
	Crude	Age standardized	Crude	Age standardized	Crude	Age standardized
	%** (95% CI)	% (95% CI)	% (95% CI)	% (95% CI)	% (95% CI)	% (95% CI)
State Medicaid expansion status						
**Medicaid expansion states^††^**						
<100 of FPL	46.8 (43.9–49.6)	46.8 (44.0–49.7)	48.5 (47.0–50.0)	49.1 (47.6–50.6)	29.5 (28.2–30.9)	29.9 (28.6–31.3)
≥100%–≤400% of FPL	56.5 (55.2–57.9)	55.2 (53.8–56.5)	43.0 (42.1–43.9)	43.9 (43.0–44.9)	30.9 (30.1–31.7)	30.2 (29.4–31.1)
>400% of FPL	70.0 (69.0–71.0)	69.6 (68.5–70.6)	39.4 (38.6–40.2)	40.6 (39.7–41.5)	41.0 (40.3–41.8)	39.4 (38.5–40.3)
Unknown	62.1 (60.2–63.9)	60.9 (59.0–62.8)	37.8 (36.5–39.0)	41.5 (40.3–42.8)	31.6 (30.4–32.8)	32.0 (30.8–33.2)
**Medicaid nonexpansion states^§§^**						
<100 of FPL	43.9 (41.5–46.3)	43.8 (41.5–46.2)	52.3 (51.0–53.7)	52.4 (51.1–53.7)	26.3 (25.2–27.4)	26.5 (25.4–27.6)
≥100%–≤400% of FPL	56.3 (55.2–57.5)	55.2 (54.0–56.3)	42.9 (42.1–43.7)	44.0 (43.2–44.8)	31.4 (30.7–32.1)	30.8 (30.1–31.5)
>400% of FPL	68.6 (67.5–69.6)	67.9 (66.9–69.0)	38.4 (37.6–39.2)	39.0 (38.1–39.9)	40.6 (39.9–41.4)	39.1 (38.2–40.0)
Unknown	56.3 (54.2–58.5)	54.7 (52.5–56.9)	34.4 (33.0–35.8)	38.7 (37.1–40.2)	30.7 (29.4–32.0)	30.5 (29.2–31.9)
**Expanded geographic regions^¶¶^**						
**New England**						
<100 of FPL	57.7 (52.4–62.9)	57.7 (52.4–62.9)	50.6 (47.4–53.8)	51.9 (48.9–54.8)	31.5 (28.7–34.5)	31.9 (29.1–34.8)
≥100%–≤400% of FPL	65.9 (63.7–68.0)	64.8 (62.6–67.0)	42.4 (40.7–44.0)	44.3 (42.5–46.1)	36.1 (34.6–37.7)	35.0 (33.4–36.7)
>400% of FPL	77.5 (76.0–79.0)	77.2 (75.6–78.7)	37.6 (36.3–39.0)	40.3 (38.7–41.8)	44.9 (43.6–46.3)	43.9 (42.2–45.6)
Unknown	67.3 (64.1–70.3)	66.3 (63.0–69.5)	41.3 (39.2–43.4)	44.0 (42.0–46.0)	35.9 (34.0–37.9)	36.6 (34.7–38.5)
**Middle Atlantic**						
<100 of FPL	53.4 (48.6–58.0)	53.3 (48.7–57.8)	58.0 (55.2–60.7)	58.0 (55.2–60.6)	31.9 (29.4–34.5)	32.1 (29.7–34.6)
≥100%–≤400% of FPL	58.5 (56.1–60.8)	57.5 (55.1–60.0)	47.7 (46.0–49.4)	48.7 (47.0–50.4)	29.8 (28.3–31.3)	29.0 (27.5–30.5)
>400% of FPL	68.9 (67.1–70.6)	68.6 (66.8–70.3)	42.8 (41.5–44.2)	44.2 (42.6–45.7)	39.4 (38.1–40.8)	37.8 (36.3–39.3)
Unknown	60.5 (56.5–64.3)	59.5 (55.5–63.5)	39.6 (36.9–42.4)	44.5 (41.6–47.4)	33.6 (31.2–36.2)	33.1 (30.6–35.7)
**East North Central**						
<100 of FPL	46.7 (41.6–51.8)	47.1 (42.2–52.1)	49.2 (46.3–52.2)	49.6 (46.7–52.4)	27.7 (25.2–30.4)	28.3 (25.8–31.0)
≥100%–≤400% of FPL	56.8 (54.6–59.0)	55.5 (53.2–57.8)	34.5 (33.0–36.1)	36.0 (34.4–37.6)	30.5 (29.1–32.0)	29.8 (28.4–31.3)
>400% of FPL	67.5 (65.7–69.3)	67.2 (65.3–69.0)	29.4 (28.0–30.8)	30.7 (29.2–32.2)	40.3 (38.8–41.7)	38.7 (37.1–40.4)
Unknown	60.5 (57.8–63.1)	59.9 (57.2–62.6)	37.4 (35.6–39.2)	40.6 (38.8–42.5)	28.2 (26.6–29.8)	28.3 (26.7–29.9)
**West North Central**						
<100 of FPL	45.0 (40.3–49.8)	45.1 (40.4–49.9)	44.8 (42.4–47.2)	46.1 (43.8–48.4)	33.3 (31.1–35.6)	34.2 (32.0–36.5)
≥100%–≤400% of FPL	54.4 (52.5–56.2)	53.1 (51.2–55.0)	33.9 (32.7–35.2)	34.8 (33.6–36.1)	34.3 (33.2–35.5)	33.7 (32.5–34.9)
>400% of FPL	69.8 (68.3–71.2)	69.3 (67.8–70.7)	28.2 (27.1–29.4)	29.3 (28.0–30.6)	45.1 (43.9–46.3)	43.5 (42.1–44.9)
Unknown	59.2 (55.8–62.5)	57.9 (54.4–61.3)	25.3 (23.1–27.5)	30.7 (28.2–33.2)	34.0 (31.8–36.2)	34.7 (32.4–37.0)
**South Atlantic**						
<100 of FPL	45.4 (41.6–49.2)	44.9 (41.3–48.7)	56.9 (54.7–59.1)	57.1 (55.0–59.3)	24.0 (22.2–25.8)	24.2 (22.4–26.0)
≥100%–≤400% of FPL	58.9 (56.9–60.8)	57.6 (55.6–59.6)	49.5 (48.1–50.9)	50.8 (49.4–52.2)	30.6 (29.4–31.8)	30.0 (28.8–31.2)
>400% of FPL	70.3 (68.5–72.1)	69.7 (67.8–71.5)	45.6 (44.2–47.0)	46.2 (44.6–47.8)	39.5 (38.2–40.8)	38.1 (36.6–39.6)
Unknown	59.1 (55.7–62.5)	56.7 (53.2–60.1)	41.3 (38.9–43.8)	45.9 (43.3–48.5)	29.0 (27.0–31.1)	28.5 (26.5–30.7)
**East South Central**						
<100 of FPL	43.9 (39.8–48.0)	43.6 (39.6–47.7)	55.1 (52.3–57.9)	54.6 (51.8–57.3)	27.5 (25.2–29.9)	27.8 (25.5–30.2)
≥100%–≤400% of FPL	55.5 (52.9–58.0)	54.3 (51.7–56.9)	44.8 (42.9–46.7)	46.5 (44.6–48.5)	33.8 (32.1–35.5)	32.9 (31.2–34.7)
>400% of FPL	68.0 (65.4–70.6)	67.5 (64.9–70.1)	36.9 (34.8–39.2)	38.6 (36.0–41.2)	44.1 (42.0–46.3)	42.9 (40.4–45.4)
Unknown	59.7 (55.6–63.7)	57.5 (53.1–61.7)	35.0 (31.9–38.2)	38.4 (35.2–41.8)	32.1 (29.3–34.9)	32.3 (29.4–35.3)
**West South Central**						
<100 of FPL	38.8 (33.6–44.2)	38.7 (33.6–44.1)	48.0 (45.2–50.8)	48.0 (45.2–50.8)	26.6 (24.4–29.0)	26.8 (24.6–29.2)
≥100%–≤400% of FPL	51.8 (48.6–54.9)	50.3 (47.1–53.5)	41.0 (39.1–43.0)	41.7 (39.8–43.6)	33.3 (31.5–35.1)	32.9 (31.1–34.7)
>400% of FPL	66.6 (63.8–69.3)	65.8 (62.9–68.6)	38.6 (36.6–40.7)	38.3 (36.2–40.5)	42.6 (40.6–44.6)	41.4 (39.1–43.7)
Unknown	47.8 (42.4–53.3)	47.3 (41.7–52.9)	31.7 (28.7–34.9)	35.3 (32.0–38.8)	31.8 (28.8–34.8)	31.9 (28.9–35.0)
**Mountain**						
<100 of FPL	42.5 (38.7–46.4)	42.3 (38.4–46.2)	41.1 (39.0–43.2)	41.9 (39.9–43.9)	28.3 (26.6–30.2)	29.1 (27.3–30.9)
≥100%–≤400% of FPL	51.6 (49.7–53.5)	50.0 (48.1–51.9)	38.1 (36.8–39.4)	38.4 (37.2–39.7)	30.2 (29.1–31.3)	30.0 (28.8–31.1)
>400% of FPL	66.3 (64.7–67.7)	65.4 (63.9–66.9)	35.9 (34.8–37.1)	36.1 (34.8–37.4)	38.6 (37.6–39.7)	36.7 (35.4–38.0)
Unknown	56.3 (53.1–59.4)	53.8 (50.4–57.1)	32.3 (30.0–34.7)	36.3 (33.8–38.8)	30.6 (28.6–32.7)	30.7 (28.6–32.9)
**Pacific**						
<100 of FPL	43.1 (37.2–49.1)	43.2 (37.3–49.3)	44.1 (41.1–47.0)	45.0 (42.1–48.0)	28.1 (25.5–30.9)	28.2 (25.7–31.0)
≥100%–≤400% of FPL	55.1 (51.6–58.6)	53.6 (50.0–57.1)	46.4 (44.2–48.7)	46.9 (44.7–49.1)	28.3 (26.5–30.3)	27.8 (25.9–29.7)
>400% of FPL	71.4 (68.6–74.0)	70.6 (67.8–73.3)	44.5 (42.6–46.5)	45.3 (43.2–47.4)	39.8 (37.9–41.7)	38.0 (36.0–40.1)
Unknown	65.9 (59.2–72.1)	62.8 (55.7–69.3)	32.5 (28.6–36.7)	37.1 (32.9–41.4)	31.4 (27.3–35.7)	33.2 (29.1–37.5)
**Overall**						
<100 of FPL	45.4 (43.5–47.2)	45.4 (43.5–47.2)	50.3 (49.3–51.3)	50.7 (49.7–51.7)	28.0 (27.1–28.8)	28.3 (27.4–29.2)
≥100%–≤400% of FPL	56.4 (55.5–57.3)	55.2 (54.2–56.1)	43.0 (42.3–43.6)	44.0 (43.4–44.6)	31.2 (30.6–31.7)	30.5 (30.0–31.1)
>400% of FPL	69.4 (68.6–70.1)	68.8 (68.1–69.6)	38.9 (38.4–39.5)	39.9 (39.2–40.5)	40.9 (40.3–41.4)	39.3 (38.6–39.9)
Unknown	59.5 (58.1–60.9)	58.1 (56.7–59.5)	36.3 (35.4–37.2)	40.4 (39.4–41.4)	31.2 (30.3–32.1)	31.4 (30.5–32.3)

**Abbreviations:** CI = confidence interval; FPL = federal poverty level; HIV = human immunodeficiency virus; n = sample size; N = weighted population.

* Age standardized to the 2000 projected population for the United States.

^†^ Adults aged 50–64 years who have had a fecal occult blood test (FOBT) within the past year, or sigmoidoscopy within the past 5 years and an FOBT within the past 3 years, or colonoscopy within the past 10 years (n = 133,620, N = 57,378,775).

**^§^** Adults aged 18–64 years ever tested for HIV (n = 269,889, N = 174,047,283).

^¶^ Adults aged 18–64 years who received an influenza vaccination within the preceding 12 months (n = 281,684, N = 181,381,790).

** Weighted percentage.

**^††^** States that expanded Medicaid as of January 1, 2014 (n = 26): Arizona, Arkansas, California, Colorado, Connecticut, Delaware, District of Columbia, Hawaii, Illinois, Iowa, Kentucky, Maryland, Massachusetts, Michigan, Minnesota, Nevada, New Jersey, New Mexico, New York, North Dakota, Ohio, Oregon, Rhode Island, Vermont, Washington, and West Virginia.

^§§^ States that did not expand Medicaid as of January 1, 2014 (n = 25): Alabama, Alaska, Florida, Georgia, Idaho, Indiana, Kansas, Louisiana, Maine, Mississippi, Missouri, Montana, Nebraska, New Hampshire, North Carolina, Oklahoma, Pennsylvania, South Carolina, South Dakota, Tennessee, Texas, Utah, Virginia, Wisconsin, and Wyoming.

^¶¶^ Expanded geographic regions are the four census regions subdivided into nine regions. For this report, the nine census regions were modified by moving Delaware, District of Columbia, and Maryland into the Middle Atlantic region. *New England:* Connecticut, Maine, Massachusetts, New Hampshire, Rhode Island, and Vermont. *Middle Atlantic*: Delaware, District of Columbia, Maryland, New Jersey, New York, and Pennsylvania. *East North Central:* Illinois, Indiana, Michigan, Ohio, and Wisconsin. *West North Central*: Iowa, Kansas, Minnesota, Missouri, Nebraska, North Dakota, and South Dakota. *South Atlantic:* Florida, Georgia, North Carolina, South Carolina, Virginia, and West Virginia. *East South Central:* Alabama, Kentucky, Mississippi, and Tennessee. *West South Central*: Arkansas, Louisiana, Oklahoma, and Texas*. Mountain:* Arizona, Colorado, Idaho, Montana, Nevada, New Mexico, Utah, and Wyoming. *Pacific:* Alaska, California, Hawaii, Oregon, and Washington.

#### HIV Testing for Adults Aged 18–64 Years

Among adults aged 18–64 years, the median estimated prevalence of having ever been tested for HIV was 40.4% and ranged from 24.4% in Utah to 74.4% in the District of Columbia. Crude and age-standardized state-specific estimates for CPS use (including HIV testing) are available (supplemental Table S3 https://stacks.cdc.gov/view/cdc/43255). No difference in the estimated prevalence by state Medicaid expansion status was found (Table 4). Among expanded geographic regions, the estimated prevalence ranged from 33.7% in the West North Central region to 50.0% in the South Atlantic region (Table 4).

The estimated prevalence of HIV testing generally decreased with increasing level of income (% of FPL) in Medicaid expansion states and nonexpansion states, and in each expanded geographic region, except in the Pacific region where no difference by FPL category was found and in the West South Central region where no difference among adults with household income ≥100%–≤400% of FPL and those with household income >400% of FPL was found (Table 5).

#### Influenza Vaccination

Among adults aged 18–64 years, the median estimated prevalence of influenza vaccination within the preceding 12 months was 34.6% and ranged from 24.1% in Florida to 44.7% in South Dakota. Crude and age-standardized state-specific estimates for CPS use (including influenza vaccination) are available (supplemental Table S3 https://stacks.cdc.gov/view/cdc/43255). No difference in the estimated prevalence by state Medicaid expansion status was found (Table 4). Among expanded geographic regions, the estimated prevalence ranged from 31.3% in the South Atlantic region to 38.2% in the New England region (Table 4).

In Medicaid expansion states, the estimated prevalence of influenza vaccination was similar among adults with household income <100% of FPL (29.9%) and those with household income ≥100%–≤400% of FPL (30.2%); in nonexpansion states, the estimated prevalence of influenza vaccination increased with increasing level of income (% of FPL). Estimates of influenza vaccination were similar among adults with household income <100% of FPL and those with household income ≥100%–≤400% of FPL in six of the nine expanded geographic regions (New England, Middle Atlantic, East North Central, West North Central, Mountain, and Pacific), whereas estimates of influenza vaccination increased with increasing level of income (% of FPL) in the South Atlantic, East South Central, and West South Central regions (Table 5).

#### Breast Cancer Screening for Women Aged 50–64 Years

Among women aged 50–64 years, the median estimated prevalence of having a mammogram within the preceding 2 years was 76.2% and ranged from 62.9% in Arkansas to 87.3% in Massachusetts. Crude and age-standardized state-specific estimates for CPS use (including breast cancer screening) are available (supplemental Table S3 https://stacks.cdc.gov/view/cdc/43255). The estimated prevalence was 78.5% in Medicaid expansion states and 75.5% in nonexpansion states (Table 6). Among expanded geographic regions, the estimated prevalence ranged from 71.7% in the Mountain region to 84.3% in the New England region (Table 6).

**TABLE 6:** Crude and age-standardized* prevalence estimates of clinical preventive services use among women, by state Medicaid expansion status and expanded geographic regions — Behavioral Risk Factor Surveillance System, United States, 2014

Category	Mammogram^†^		Pap test^§^	
	Crude	Age standardized	Crude	Age standardized
	%^¶^ (95% CI)	% (95% CI)	% (95% CI)	% (95% CI)
**State Medicaid expansion status**				
Medicaid expansion states**	78.6 (77.8–79.4)	78.5 (77.6–79.3)	83.0 (82.4–83.6)	83.0 (82.3–83.6)
Medicaid nonexpansion states^††^	75.6 (74.8–76.4)	75.5 (74.7–76.3)	81.5 (80.8–82.1)	81.5 (80.9–82.1)
**Expanded geographic regions^§§^**				
New England	84.3 (83.1–85.4)	84.3 (83.1–85.4)	87.1 (86.1–88.0)	87.0 (85.9–87.9)
Middle Atlantic	78.1 (76.6–79.6)	78.1 (76.5–79.5)	83.0 (81.9–84.1)	83.0 (81.7–84.1)
East North Central	76.8 (75.3–78.1)	76.7 (75.3–78.1)	82.1 (81.0–83.2)	82.2 (81.0–83.3)
West North Central	76.3 (75.0–77.6)	76.1 (74.8–77.4)	83.3 (82.4–84.2)	83.3 (82.4–84.2)
South Atlantic	77.3 (76.0–78.6)	77.1 (75.8–78.5)	82.8 (81.7–83.7)	82.8 (81.8–83.8)
East South Central	75.3 (73.5–76.9)	75.0 (73.2–76.7)	83.6 (82.3–84.9)	83.6 (82.2–84.8)
West South Central	74.3 (72.2–76.3)	74.2 (72.1–76.2)	78.5 (76.9–80.0)	78.5 (76.8–80.0)
Mountain	72.1 (70.9–73.4)	71.7 (70.4–73.0)	80.3 (79.3–81.2)	80.3 (79.2–81.2)
Pacific	80.1 (77.9–82.1)	79.9 (77.6–81.9)	82.5 (80.9–84.0)	82.4 (80.8–83.9)
**Overall**	**77.2 (76.6–77.8)**	**77.1 (76.5–77.7)**	**82.3 (81.8–82.7)**	**82.3 (81.9–82.8)**

**Abbreviations:** CI = confidence interval; n = sample size; N = weighted population; Pap = Papanicolaou.

* Age standardized to the 2000 projected population for the United States.

^†^ Within the past 2 years among women aged 50–64 years (n = 79,880, N = 30,796,537).

^§^ Within the past 3 years among women aged 21–64 years who have not had a hysterectomy (n = 118,135, N = 68,270,683).

^¶^ Weighted percentage.

** States that expanded Medicaid as of January 1, 2014 (n = 26): Arizona, Arkansas, California, Colorado, Connecticut, Delaware, District of Columbia, Hawaii, Illinois, Iowa, Kentucky, Maryland, Massachusetts, Michigan, Minnesota, Nevada, New Jersey, New Mexico, New York, North Dakota, Ohio, Oregon, Rhode Island, Vermont, Washington, and West Virginia.

^††^ States that did not expand Medicaid as of January 1, 2014 (n = 25): Alabama, Alaska, Florida, Georgia, Idaho, Indiana, Kansas, Louisiana, Maine, Mississippi, Missouri, Montana, Nebraska, New Hampshire, North Carolina, Oklahoma, Pennsylvania, South Carolina, South Dakota, Tennessee, Texas, Utah, Virginia, Wisconsin, and Wyoming.

^§§^ Expanded geographic regions are the four census regions subdivided into nine regions. For this report, the nine census regions were modified by moving Delaware, District of Columbia, and Maryland into the Middle Atlantic region. *New England:* Connecticut, Maine, Massachusetts, New Hampshire, Rhode Island, and Vermont. *Middle Atlantic:* Delaware, District of Columbia, Maryland, New Jersey, New York, and Pennsylvania. *East North Central:* Illinois, Indiana, Michigan, Ohio, and Wisconsin. *West North Central*: Iowa, Kansas, Minnesota, Missouri, Nebraska, North Dakota, and South Dakota. *South Atlantic:* Florida, Georgia, North Carolina, South Carolina, Virginia, and West Virginia. *East South Central:* Alabama, Kentucky, Mississippi, and Tennessee. *West South Central:* Arkansas, Louisiana, Oklahoma, and Texas. *Mountain:* Arizona, Colorado, Idaho, Montana, Nevada, New Mexico, Utah, and Wyoming. *Pacific:* Alaska, California, Hawaii, Oregon, and Washington.

In Medicaid expansion states, the estimated prevalence of breast cancer screening was similar among women aged 50–64 years with household income <100% of FPL (71.4%) and those with household income ≥100%–≤400% of FPL (74.5%); in nonexpansion states, estimates of breast cancer screening increased with increasing level of income (% of FPL). Estimates of breast cancer screening were similar among women with household income <100% of FPL and those with household income ≥100%–≤400% of FPL in five of the nine expanded geographic regions (New England, Middle Atlantic, West South Central, Mountain, and Pacific); estimates increased with increasing level of income (% of FPL) in the remaining expanded geographic regions (Table 7).

**TABLE 7:** Crude and age-standardized* prevalence estimates of breast and cervical cancer screenings among women, by federal poverty level, state Medicaid expansion status, and expanded geographic regions — Behavioral Risk Factor Surveillance System, United States, 2014

Category	Mammogram^†^		Pap test^§^	
	Crude	Age standardized	Crude	Age standardized
	%^¶^ (95% CI)	% (95% CI)	% (95% CI)	% (95% CI)
**State Medicaid expansion status**				
Medicaid expansion states**				
<100 of FPL	71.3 (68.0–74.4)	71.4 (68.1–74.5)	78.1 (76.4–79.8)	77.2 (75.3–78.9)
≥100%–≤400% of FPL	74.7 (73.2–76.1)	74.5 (73.0–76.0)	81.0 (79.8–82.1)	81.0 (79.7–82.1)
>400% of FPL	83.3 (82.2–84.4)	83.3 (82.1–84.4)	89.3 (88.4–90.1)	89.3 (88.3–90.2)
Unknown	80.6 (78.6–82.4)	80.4 (78.2–82.3)	79.0 (77.3–80.7)	79.0 (77.3–80.7)
**Medicaid nonexpansion states^††^**				
<100 of FPL	63.9 (61.2–66.6)	64.0 (61.2–66.6)	73.8 (72.1–75.4)	72.2 (70.4–73.9)
≥100%–≤400% of FPL	72.3 (71.0–73.6)	72.1 (70.8–73.5)	80.3 (79.2–81.2)	80.3 (79.3–81.3)
>400% of FPL	83.6 (82.4–84.7)	83.5 (82.3–84.6)	89.9 (89.0–90.7)	90.1 (89.2–91.0)
Unknown	75.8 (73.4–78.1)	75.0 (72.4–77.5)	77.1 (75.0–79.0)	77.1 (75.0–79.0)
**Expanded geographic regions^§§^**				
**New England**				
<100 of FPL	77.9 (72.5–82.5)	77.9 (72.5–82.5)	81.3 (77.5–84.5)	81.5 (77.9–84.7)
≥100%–≤400% of FPL	79.5 (77.1–81.8)	79.4 (76.8–81.7)	86.1 (84.4–87.7)	86.2 (84.3–87.9)
>400% of FPL	88.6 (87.2–89.9)	88.7 (87.2–89.9)	92.0 (90.9–93.0)	92.5 (91.2–93.6)
Unknown	83.7 (80.5–86.5)	83.5 (80.0–86.4)	82.5 (79.8–84.8)	82.7 (80.1–85.0)
**Middle Atlantic**				
<100 of FPL	74.3 (69.2–78.8)	74.5 (69.5–78.9)	78.4 (75.2–81.4)	78.0 (74.8–80.9)
≥100%–≤400% of FPL	74.1 (71.3–76.7)	74.1 (71.2–76.8)	82.2 (80.3–84.0)	82.3 (80.3–84.2)
>400% of FPL	81.2 (79.0–83.2)	81.2 (79.0–83.1)	88.3 (86.5–89.8)	88.1 (86.1–89.9)
Unknown	81.3 (76.8–85.1)	81.1 (76.2–85.2)	75.2 (71.2–78.8)	74.8 (70.7–78.5)
**East North Central**				
<100 of FPL	63.9 (57.9–69.5)	63.9 (57.9–69.4)	78.1 (74.8–81.1)	75.8 (72.3–78.9)
≥100%–≤400% of FPL	72.8 (70.3–75.2)	72.5 (69.8–75.0)	79.1 (76.9–81.2)	79.2 (76.9–81.3)
>400% of FPL	82.6 (80.5–84.5)	82.6 (80.5–84.5)	88.9 (87.4–90.3)	89.2 (87.6–90.7)
Unknown	79.4 (76.4–82.2)	79.3 (76.2–82.1)	79.0 (76.5–81.4)	79.1 (76.5–81.4)
**West North Central**				
<100 of FPL	58.0 (52.0–63.8)	58.0 (52.0–63.8)	72.9 (69.6–75.9)	69.8 (66.4–73.0)
≥100%–≤400% of FPL	70.9 (68.6–73.1)	70.6 (68.2–72.8)	80.6 (78.9–82.2)	80.6 (78.9–82.2)
>400% of FPL	84.3 (82.6–85.9)	84.2 (82.5–85.8)	91.0 (90.0–92.0)	91.4 (90.3–92.4)
Unknown	77.4 (73.4–80.8)	76.7 (72.5–80.4)	81.0 (78.2–83.5)	81.0 (78.2–83.5)
**South Atlantic**				
<100 of FPL	64.6 (60.0–69.0)	64.6 (60.0–69.0)	73.5 (70.5–76.2)	72.0 (69.1–74.8)
≥100%–≤400% of FPL	74.0 (71.7–76.1)	73.8 (71.5–76.1)	81.5 (79.8–83.1)	81.7 (80.0–83.2)
>400% of FPL	86.5 (84.8–88.0)	86.4 (84.6–87.9)	91.8 (90.6–92.9)	91.9 (90.6–93.1)
Unknown	77.6 (74.1–80.8)	76.5 (72.6–79.9)	79.7 (76.3–82.7)	79.3 (75.9–82.4)
**East South Central**				
<100 of FPL	61.0 (55.9–65.8)	60.4 (55.3–65.3)	78.5 (75.3–81.4)	76.5 (73.3–79.4)
≥100%–≤400% of FPL	74.1 (71.4–76.6)	74.0 (71.2–76.6)	81.5 (79.1–83.6)	81.5 (79.1–83.6)
>400% of FPL	84.2 (81.4–86.6)	84.1 (81.3–86.5)	92.2 (90.3–93.7)	92.6 (90.7–94.2)
Unknown	75.9 (70.9–80.2)	75.6 (70.4–80.1)	83.0 (79.0–86.4)	83.1 (79.0–86.4)
**West South Central**				
<100 of FPL	67.4 (61.7–72.7)	67.5 (61.9–72.6)	71.9 (68.2–75.3)	70.7 (66.8–74.3)
≥100%–≤400% of FPL	72.4 (69.0–75.6)	72.2 (68.7–75.4)	77.9 (75.1–80.4)	77.8 (75.0–80.3)
>400% of FPL	80.7 (77.2–83.7)	80.7 (77.2–83.7)	87.5 (84.9–89.8)	87.8 (85.1–90.1)
Unknown	71.1 (64.2–77.2)	70.2 (62.9–76.6)	74.2 (69.3–78.5)	74.5 (69.8–78.7)
**Mountain**				
<100 of FPL	65.2 (60.7–69.4)	65.1 (60.6–69.4)	73.7 (71.1–76.2)	72.3 (69.7–74.8)
≥100%–≤400% of FPL	66.8 (64.6–69.0)	66.2 (63.8–68.5)	79.6 (78.0–81.2)	79.5 (77.8–81.0)
>400% of FPL	79.6 (77.8–81.2)	79.3 (77.6–81.0)	87.6 (86.1–88.9)	87.8 (86.2–89.3)
Unknown	70.4 (66.8–73.8)	68.8 (64.8–72.5)	74.2 (70.6–77.5)	74.3 (70.8–77.5)
**Pacific**				
<100 of FPL	72.1 (64.9–78.3)	72.3 (65.3–78.4)	78.8 (75.1–82.0)	77.7 (73.9–81.1)
≥100%–≤400% of FPL	76.8 (72.9–80.2)	76.7 (72.7–80.2)	79.8 (76.8–82.6)	79.8 (76.7–82.5)
>400% of FPL	85.0 (82.2–87.4)	84.8 (81.9–87.3)	89.1 (86.7–91.1)	88.9 (86.3–91.0)
Unknown	85.0 (78.7–89.7)	85.8 (79.7–90.3)	78.7 (72.9–83.6)	78.8 (73.1–83.5)
**Overall**				
<100 of FPL	67.7 (65.5–69.7)	67.7 (65.5–69.7)	76.0 (74.8–77.2)	74.8 (73.5–76.0)
≥100%–≤400% of FPL	73.5 (72.5–74.5)	73.3 (72.3–74.3)	80.6 (79.8–81.4)	80.7 (79.9–81.4)
>400% of FPL	83.4 (82.6–84.2)	83.4 (82.6–84.2)	89.5 (88.9–90.1)	89.7 (89.0–90.3)
Unknown	78.4 (76.8–79.8)	77.9 (76.3–79.5)	78.2 (76.9–79.5)	78.2 (76.9–79.5)

**Abbreviations:** CI = confidence interval; FPL = federal poverty level; n = sample size; N = weighted population; Pap = Papanicolaou.

* Age standardized to the 2000 projected population for the United States.

^†^ Within past 2 years among women aged 50–64 years (n = 79,880, N = 30,796,537).

^§^ Within past 3 years among women aged 21–64 years who have not had a hysterectomy (n = 118,135, N = 68,270,683).

^¶^ Weighted percentage.

** States that expanded Medicaid as of January 1, 2014 (n = 26): Arizona, Arkansas, California, Colorado, Connecticut, Delaware, District of Columbia, Hawaii, Illinois, Iowa, Kentucky, Maryland, Massachusetts, Michigan, Minnesota, Nevada, New Jersey, New Mexico, New York, North Dakota, Ohio, Oregon, Rhode Island, Vermont, Washington, and West Virginia.

**^††^** States that did not expand Medicaid as of January 1, 2014 (n = 25): Alabama, Alaska, Florida, Georgia, Idaho, Indiana, Kansas, Louisiana, Maine, Mississippi, Missouri, Montana, Nebraska, New Hampshire, North Carolina, Oklahoma, Pennsylvania, South Carolina, South Dakota, Tennessee, Texas, Utah, Virginia, Wisconsin, and Wyoming.

^§§^ Expanded geographic regions are the four census regions subdivided into nine regions. For this report, the nine census regions were modified by moving Delaware, District of Columbia, and Maryland into the Middle Atlantic region. *New England:* Connecticut, Maine, Massachusetts, New Hampshire, Rhode Island, and Vermont. *Middle Atlantic:* Delaware, District of Columbia, Maryland, New Jersey, New York, and Pennsylvania. *East North Central:* Illinois, Indiana, Michigan, Ohio, and Wisconsin. *West North Central:* Iowa, Kansas, Minnesota, Missouri, Nebraska, North Dakota, and South Dakota. *South Atlantic:* Florida, Georgia, North Carolina, South Carolina, Virginia, and West Virginia. *East South Central:* Alabama, Kentucky, Mississippi, and Tennessee. *West South Central:* Arkansas, Louisiana, Oklahoma, and Texas. *Mountain:* Arizona, Colorado, Idaho, Montana, Nevada, New Mexico, Utah, and Wyoming. *Pacific:* Alaska, California, Hawaii, Oregon, and Washington.

#### Cervical Cancer Screening for Women Aged 21–64 Years

Among women aged 21–64 years, the median estimated prevalence of having received cervical cancer screening within the preceding 3 years was 82.7% and ranged from 76.2% in Idaho to 87.8% in Massachusetts. Crude and age-standardized state-specific estimates for CPS use (including cervical cancer screening) are available (supplemental Table S3 https://stacks.cdc.gov/view/cdc/43255). The estimated prevalence was 83.0% in Medicaid expansion states and 81.5% in nonexpansion states (Table 6). Among expanded geographic regions, the estimated prevalence ranged from 78.5% in the West South Central region to 87.0% in the New England region (Table 6).

The estimated prevalence of cervical cancer screening ranged from 69.8% among women with household income <100% of FPL in the West North Central region to 92.6% among women with household income >400% of FPL in the East South Central region. The estimated prevalence of cervical cancer screening increased with increasing level of income (% of FPL) in Medicaid expansion and nonexpansion states. Among the expanded geographic regions, however, less variability by FPL category was found. For example, the estimated prevalence of cervical cancer screening was similar among women with household income <100% of FPL and those with household income ≥100%–≤400% of FPL in five of the nine expanded geographic regions (New England, Middle Atlantic, East North Central, East South Central, and Pacific). The estimated prevalence of cervical cancer screening among women with household income <100% of FPL ranged from 69.8% in the West North Central region to 81.5% in the New England region. Among women with household income ≥100%–≤400% of FPL, estimates ranged from 77.8% in the West South Central region to 86.2% in the New England region. Among women with household income >400% of FPL, estimates ranged from 87.8% in the Mountain and West South Central regions to 92.5% in the New England region and 92.6% in the East South Central region (Table 7).

### Primary Source of Health Insurance Coverage

#### Overall and by State Medicaid Expansion Status, State, and FPL

In the 43 states using questions from the optional module, 17.1% of adults aged 18–64 years had no primary source of health insurance coverage, 19.4% had public health plan coverage, and 63.4% had private health insurance coverage. In states expanding Medicaid eligibility, 15.0% had no primary source of health insurance, 21.1% had public health plan coverage, and 63.9% had private health insurance coverage. In nonexpansion states, 19.9% had no primary source of health insurance, 17.2% had public health plan coverage, and 62.9% had private health insurance coverage (Table 8).

**TABLE 8:** Crude and age-standardized* prevalence estimates of no primary source of health insurance coverage, public health plan coverage, and private health insurance coverage at the time of interview among adults aged 18–64 years, by state Medicaid expansion status and federal poverty level — Behavioral Risk Factor Surveillance System, 43 states,^†^ 2014

Category	n^§^	No primary source of health insurance coverage^¶^		Public health plan coverage**		Private health insurance coverage^††^	
		Crude	Age standardized	Crude	Age standardized	Crude	Age standardized
		%^§§^ (95% CI)	% (95% CI)	% (95% CI)	% (95% CI)	% (95% CI)	% (95% CI)
**Medicaid expansion states^¶¶^**	**124,776**	**14.2 (13.8–14.6)**	**15.0 (14.6–15.5)**	**21.1 (20.7–21.5)**	**21.1 (20.7–21.6)**	**64.7 (64.2–65.2)**	**63.9 (63.3–64.4)**
<100 of FPL	13,467	29.8 (28.4–31.2)	29.6 (28.2–31.0)	49.8 (48.2–51.3)	50.7 (49.2–52.2)	20.5 (19.2–21.8)	19.7 (18.6–21.0)
≥100%–≤400% of FPL	40,723	16.7 (16.0–17.5)	17.3 (16.6–18.2)	22.6 (21.9–23.4)	22.1 (21.3–22.9)	60.6 (59.7–61.5)	60.5 (59.6–61.5)
>400% of FPL	50,819	3.3 ( 2.9–3.6)	3.6 ( 3.2–4.0)	5.1 ( 4.7–5.4)	5.1 ( 4.7–5.6)	91.7 (91.2–92.1)	91.3 (90.7–91.9)
Unknown	19,767	17.7 (16.7–18.8)	18.3 (17.3–19.4)	25.4 (24.4–26.4)	25.7 (24.6–26.8)	56.9 (55.7–58.1)	56.0 (54.8–57.2)
Medicaid nonexpansion states***	**101,440**	**19.0 (18.6–19.5)**	**19.9 (19.4–20.4)**	**17.6 (17.2–18.0)**	**17.2 (16.8–17.6)**	**63.4 (62.9–63.9)**	**62.9 (62.3–63.4)**
<100 of FPL	13,194	41.7 (40.3–43.1)	41.6 (40.2–42.9)	37.1 (35.8–38.4)	37.9 (36.6–39.2)	21.2 (20.1–22.4)	20.6 (19.5–21.7)
≥100%–≤400% of FPL	39,400	20.1 (19.4–20.8)	20.7 (20.0–21.5)	17.5 (16.8–18.1)	16.7 (16.1–17.3)	62.5 (61.6–63.3)	62.6 (61.7–63.4)
>400% of FPL	36,609	3.8 ( 3.5–4.2)	4.4 ( 3.9–4.9)	5.9 ( 5.5–6.3)	5.6 ( 5.2–6.1)	90.2 (89.7–90.8)	90.0 (89.3–90.6)
Unknown	12,237	23.5 (22.1–24.8)	24.5 (23.1–26.0)	21.2 (20.0–22.4)	20.5 (19.3–21.7)	55.4 (53.9–56.8)	55.0 (53.5–56.6)
**Overall**	**226,216**	**16.3 (16.0–16.6)**	**17.1 (16.8–17.5)**	**19.6 (19.3–19.9)**	**19.4 (19.1–19.7)**	**64.1 (63.8–64.5)**	**63.4 (63.0–63.8)**
<100 of FPL	26,661	35.3 (34.3–36.3)	35.1 (34.1–36.1)	43.9 (42.9–44.9)	44.8 (43.8–45.8)	20.8 (19.9–21.7)	20.1 (19.3–21.0)
≥100%–≤400% of FPL	80,123	18.3 (17.8–18.9)	19.0 (18.4–19.5)	20.2 (19.7–20.7)	19.5 (19.0–20.1)	61.5 (60.9–62.1)	61.5 (60.8–62.1)
>400% of FPL	87,428	3.5 ( 3.3–3.8)	3.9 ( 3.6–4.3)	5.4 ( 5.2–5.7)	5.3 ( 5.0–5.7)	91.1 (90.7–91.4)	90.7 (90.3–91.2)
Unknown	32,004	19.8 (19.0–20.6)	20.5 (19.6–21.4)	23.9 (23.1–24.7)	24.0 (23.1–24.8)	56.3 (55.4–57.3)	55.6 (54.6–56.5)

**Abbreviations:** CI = confidence interval; FPL = federal poverty level; n = sample size.

* Age standardized to the 2000 projected population for the United States.

^†^ Alabama, Alaska, Arizona, Colorado, Connecticut, Delaware, District of Columbia, Georgia, Idaho, Illinois, Indiana, Iowa, Kentucky, Louisiana, Maine, Maryland, Massachusetts, Michigan, Minnesota, Mississippi, Missouri, Montana, Nebraska, Nevada, New Hampshire, New Jersey, New Mexico, New York, North Carolina, North Dakota, Ohio, Oklahoma, Oregon, Pennsylvania, Rhode Island, South Carolina, Tennessee, Utah, Vermont, Virginia, Washington, West Virginia, and Wisconsin. Data from Missouri are available from the Missouri Department of Health and Senior Services.

**^§^** Unweighted sample size.

^¶^ Includes persons who, when asked about their primary source of health care coverage, said they had none and persons who did not have any kind of health care coverage at the time of interview.

** Public health insurance includes Medicare; Medicaid or other state program; TRICARE (formerly CHAMPUS), Veterans Affairs, or military plan; Alaska Native, Indian Health Service, Tribal Health Services, or some other source.

^††^ Private health insurance includes a plan purchased through an employer or union (includes plans purchased through another person’s employer) or a plan that they or another family member purchased on their own.

^§§^ Weighted percentage.

^¶¶^ States that expanded Medicaid as of January 1, 2014 (n = 23): Arizona, Colorado, Connecticut, Delaware, District of Columbia, Illinois, Iowa, Kentucky, Maryland, Massachusetts, Michigan, Minnesota, Nevada, New Jersey, New Mexico, New York, North Dakota, Ohio, Oregon, Rhode Island, Vermont, Washington, and West Virginia.

*** States that did not expand Medicaid as of January 1, 2014 (n = 20): Alabama, Alaska, Georgia, Idaho, Indiana, Louisiana, Maine, Mississippi, Missouri, Montana, Nebraska, New Hampshire, North Carolina, Oklahoma, Pennsylvania, South Carolina, Tennessee, Utah, Virginia, and Wisconsin.

Variations in primary source of health insurance coverage existed by FPL category and state Medicaid expansion status. Among working-aged adults with no primary source of health insurance coverage or public health plan coverage, the magnitude of the variation diminished with increasing FPL category by expansion status, whereas among adults with private health insurance, estimates were similar by FPL category regardless of state Medicaid expansion status. For example, among adults with household income <100% of FPL in Medicaid expansion and nonexpansion states, the estimated prevalence of no primary source of health insurance coverage was 29.6% and 41.6%, respectively, and the estimated prevalence of public health plan coverage was 50.7% and 37.9%, respectively. Among adults with household income ≥100%–≤400% of FPL in Medicaid expansion and nonexpansion states, the estimated prevalence of no primary source of health insurance coverage was 17.3% and 20.7%, respectively, and the estimated prevalence of public health plan coverage was 22.1% and 16.7%, respectively. Among adults with household income >400% of FPL, similar prevalence patterns by primary source of insurance coverage were found in Medicaid expansion states and nonexpansion states (Table 8).

The median estimated prevalence of adults in 43 states who had Medicaid as their primary source of health insurance coverage was 7.8% and ranged from 3.5% in both Idaho and Utah to 17.9% in the District of Columbia. The median estimated prevalence of other public insurance was 11.1% and ranged from 6.5% in Iowa to 18.8% in Alaska. The median estimated prevalence of employer-based coverage was 53.8% and ranged from 41.3% in New Mexico to 63.1% in Iowa. The median estimated prevalence of other private coverage was 9.3% and ranged from 5.3% in Massachusetts to 14.7% in Idaho. The median estimated prevalence of no primary source of health insurance coverage was 16.5% and ranged from 5.9% in Massachusetts to 28.4% in Georgia. Crude and age-standardized state-specific estimates for primary source of health insurance coverage are available (supplemental Table S4 https://stacks.cdc.gov/view/cdc/43255).

#### Health Care Access and CPS Use

Among adults aged 18–64 years, those with no primary source of insurance were approximately half as likely (42.7% versus 79.8% and 81.0%, respectively) to have a usual source of health care, and approximately three fifths as likely (42.0% versus 74.4% and 68.9%, respectively) to have had a routine checkup within the preceding 12 months, compared with adults who had public health plan coverage and private health insurance coverage, respectively (Table 9). The estimated prevalence of having unmet health care need because of cost or unmet prescribed medication need because of cost was highest among adults with no primary source of insurance coverage (43.9% and 18.8%, respectively), followed by those with public health plan coverage (18.3% and 14.4%, respectively), and lowest among those with private health insurance coverage (9.0% and 6.3%, respectively). The estimated prevalence of medical debt was highest among adults with no primary source of insurance (27.8%), but similar among publically and privately insured adults (21.9% and 22.1%, respectively). For these health care access measures, similar prevalence patterns by primary source of health insurance coverage were found in Medicaid expansion states and nonexpansion states (Table 9).

**TABLE 9:** Crude and age-standardized* prevalence estimates of health care access and clinical preventive services use among adults aged 18–64 years with no primary source of health insurance coverage, public health plan coverage, or private health insurance coverage at the time of interview, by state Medicaid expansion status — Behavioral Risk Factor Surveillance System, 43 states,^†^ 2014

Category	n^§^	No primary source of health insurance coverage^¶^		Public health plan coverage^**^		Private health insurance coverage^††^	
		Crude	Age standardized	Crude	Age standardized	Crude	Age standardized
		%^§§^ (95% CI)	% (95% CI)	% (95% CI)	% (95% CI)	% (95% CI)	% (95% CI)
**Medicaid expansion states^¶¶^**							
**Health care access**							
Usual source of health care	124,317	41.3 (39.8–42.8)	42.5 (41.0–44.0)	80.8 (79.9–81.7)	79.8 (78.8–80.7)	83.7 (83.2–84.2)	82.0 (81.5–82.6)
Routine checkup within preceding 12 mos	123,374	42.1 (40.6–43.7)	42.7 (41.2–44.2)	73.6 (72.5–74.6)	72.7 (71.6–73.8)	70.3 (69.8–70.9)	69.0 (68.4–69.6)
Cost barrier to health care need during preceding 12 mos	124,504	41.5 (40.0–43.1)	41.7 (40.2–43.2)	18.3 (17.4–19.2)	18.3 (17.4–19.2)	8.1 (7.8–8.5)	8.4 (8.0–8.8)
Cost barrier to prescribed medication need during preceding 12 mos	124,142	16.1 (14.9–17.3)	16.6 (15.5–17.8)	13.8 (13.0–14.6)	13.7 (12.9–14.5)	5.6 (5.3–5.9)	5.7 (5.3–6.0)
Medical bills being paid off over time during preceding 12 mos	123,471	24.3 (23.0–25.6)	24.5 (23.3–25.9)	20.4 (19.5–21.3)	20.2 (19.2–21.2)	19.4 (18.9–19.9)	19.8 (19.3–20.3)
**CPS**							
Mammogram within past 2 yrs (women aged 50–64 yrs)	35,376	48.5 (44.7–52.2)	48.5 (44.8–52.3)	75.5 (73.7–77.3)	75.3 (73.4–77.1)	82.0 (81.1–82.8)	81.9 (81.0–82.7)
Pap test within past 3 yrs (women aged 21–64 yrs who have not had a hysterectomy)	51,860	67.5 (65.1–69.9)	66.2 (63.8–68.5)	80.7 (79.2–82.1)	80.6 (79.1–82.0)	87.0 (86.3–87.7)	86.9 (86.1–87.6)
Colorectal cancer screening*** (adults aged 50–64 yrs)	58,596	32.2 (29.5–35.0)	32.3 (29.6–35.1)	61.3 (59.6–63.0)	59.8 (58.0–61.5)	66.5 (65.6–67.3)	65.9 (65.0–66.7)
HIV test**^†††^** (adults aged 18–64 yrs)	112,626	43.7 (42.0–45.3)	42.7 (41.1–44.3)	54.4 (53.2–55.6)	56.0 (54.8–57.2)	36.5 (35.9–37.1)	38.6 (37.9–39.2)
Influenza vaccination within preceding 12 mos (adults aged 18–64 yrs)	117,754	18.2 (16.9–19.5)	18.5 (17.3–19.7)	36.6 (35.5–37.7)	35.6 (34.4–36.8)	37.9 (37.3–38.5)	36.7 (36.1–37.3)
**Medicaid nonexpansion states^§§§^**							
**Health care access**							
Usual source of health care	101,077	41.3 (40.1–42.6)	42.8 (41.6–44.0)	81.5 (80.5–82.5)	79.9 (78.7–80.9)	81.4 (80.8–81.9)	79.6 (79.0–80.2)
Routine checkup within preceding 12 mos	100,265	41.0 (39.7–42.4)	41.4 (40.1–42.7)	78.3 (77.3–79.3)	77.0 (75.8–78.1)	70.2 (69.6–70.7)	68.8 (68.1–69.4)
Cost barrier to health care need during preceding 12 mos	101,208	45.5 (44.1–46.8)	46.0 (44.7–47.3)	18.1 (17.2–19.1)	18.4 (17.4–19.5)	9.6 (9.2–10.0)	9.8 (9.4–10.2)
Cost barrier to prescribed medication need during preceding 12 mos	100,841	20.4 (19.3–21.4)	21.0 (20.0–22.0)	15.4 (14.6–16.3)	15.7 (14.7–16.7)	7.2 (6.8–7.5)	7.2 (6.8–7.6)
Medical bills being paid off over time during preceding 12 mos	100,304	30.7 (29.5–31.9)	31.0 (29.8–32.2)	24.8 (23.8–25.8)	24.5 (23.4–25.7)	24.7 (24.2–25.3)	25.2 (24.6–25.7)
**CPS**							
Mammogram within past 2 yrs (women aged 50–64 yrs)	27,283	47.2 (44.3–50.1)	47.1 (44.2–50.0)	73.8 (71.9–75.6)	73.6 (71.5–75.5)	81.0 (80.1–81.8)	80.8 (79.9–81.7)
Pap test within past 3 yrs (women aged 21–64 yrs who have not had a hysterectomy)	39,447	67.5 (65.5–69.4)	65.7 (63.8–67.6)	82.1 (80.5–83.6)	82.1 (80.5–83.6)	87.6 (86.9–88.2)	87.7 (87.0–88.4)
Colorectal cancer screening*** (adults aged 50–64 yrs)	45,802	29.1 (27.1–31.2)	29.1 (27.1–31.1)	64.7 (63.1–66.2)	63.1 (61.4–64.8)	65.4 (64.6–66.3)	64.7 (63.9–65.6)
HIV test**^†††^** (adults aged 18–64 yrs)	93,094	47.0 (45.6–48.4)	46.4 (45.1–47.8)	55.3 (54.1–56.6)	58.3 (57.0–59.6)	35.5 (34.9–36.1)	37.3 (36.6–38.0)
Influenza vaccination within preceding 12 mos (adults aged 18–64 yrs)	96,771	17.4 (16.4–18.4)	17.6 (16.6–18.6)	41.6 (40.4–42.9)	40.1 (38.7–41.4)	37.8 (37.2–38.4)	36.7 (36.1–37.4)
**Overall**							
**Health care access**							
Usual source of health care	225,394	41.3 (40.3–42.3)	42.7 (41.7–43.6)	81.1 (80.4–81.7)	79.8 (79.1–80.5)	82.7 (82.4–83.0)	81.0 (80.6–81.4)
Routine checkup within preceding 12 mos	223,639	41.6 (40.6–42.6)	42.0 (41.0–43.0)	75.4 (74.7–76.2)	74.4 (73.5–75.2)	70.3 (69.9–70.7)	68.9 (68.4–69.3)
Cost barrier to health care need during preceding 12 mos	225,712	43.5 (42.5–44.5)	43.9 (42.9–44.9)	18.2 (17.6–18.9)	18.3 (17.7–19.0)	8.8 (8.5–9.0)	9.0 (8.7–9.3)
Cost barrier to prescribed medication need during preceding 12 months	224,983	18.2 (17.5–19.0)	18.8 (18.1–19.6)	14.4 (13.9–15.0)	14.4 (13.8–15.1)	6.3 (6.1–6.5)	6.3 (6.1–6.6)
Medical bills being paid off over time during preceding 12 mos	223,775	27.5 (26.6–28.4)	27.8 (26.9–28.7)	22.1 (21.4–22.8)	21.9 (21.1–22.6)	21.7 (21.3–22.1)	22.1 (21.7–22.5)
**CPS**							
Mammogram within past 2 yrs (women aged 50–64 yrs)	62,659	47.8 (45.5–50.1)	47.8 (45.4–50.1)	74.8 (73.5–76.1)	74.6 (73.1–75.9)	81.5 (80.9–82.2)	81.4 (80.8–82.1)
Pap test within past 3 yrs (women aged 21–64 yrs who have not had a hysterectomy)	91,307	67.5 (66.0–69.0)	65.9 (64.4–67.4)	81.2 (80.1–82.3)	81.1 (80.0–82.2)	87.2 (86.8–87.7)	87.2 (86.7–87.7)
Colorectal cancer screening*** (adults aged 50–64 yrs)	104,398	30.6 (28.9–32.3)	30.6 (28.9–32.3)	62.7 (61.5–63.9)	61.2 (59.9–62.4)	66.0 (65.4–66.6)	65.4 (64.8–66.0)
HIV test**^†††^** (adults aged 18–64 yrs)	205,720	45.3 (44.3–46.4)	44.6 (43.6–45.7)	54.8 (53.9–55.7)	56.9 (56.0–57.8)	36.1 (35.6–36.5)	38.0 (37.5–38.5)
Influenza vaccination during preceding 12 mos (adults aged 18–64 yrs)	214,525	17.8 (17.0–18.6)	18.0 (17.2–18.8)	38.6 (37.7–39.4)	37.4 (36.5–38.2)	37.9 (37.4–38.3)	36.7 (36.3–37.2)

**Abbreviations:** CI = confidence interval; CPS = clinical preventive services; HIV = human immunodeficiency virus; n = sample size; Pap = Papanicolaou.

* Age standardized to the 2000 projected population for the United States.

^†^ Alabama, Alaska, Arizona, Colorado, Connecticut, Delaware, District of Columbia, Georgia, Idaho, Illinois, Indiana, Iowa, Kentucky, Louisiana, Maine, Maryland, Massachusetts, Michigan, Minnesota, Mississippi, Missouri, Montana, Nebraska, Nevada, New Hampshire, New Jersey, New Mexico, New York, North Carolina, North Dakota, Ohio, Oklahoma, Oregon, Pennsylvania, Rhode Island, South Carolina, Tennessee, Utah, Vermont, Virginia, Washington, West Virginia, and Wisconsin. Data from Missouri are available from the Missouri Department of Health and Senior Services.

^§^ Unweighted sample size.

^¶^ Includes persons who, when asked about their primary source of health care coverage, said they had none and persons who did not have any kind of health care coverage at the time of interview.

** Public health insurance includes Medicare; Medicaid or other state program; TRICARE (formerly CHAMPUS), Veterans Affairs, or military plan; Alaska Native, Indian Health Service, Tribal Health Services, or some other source.

^††^ Private health insurance includes a plan purchased through an employer or union (includes plans purchased through another person's employer) or a plan that they or another family member purchased on their own.

^§§^ Weighted percentage.

^¶¶^ States that expanded Medicaid as of January 1, 2014 (n = 23): Arizona, Colorado, Connecticut, Delaware, District of Columbia, Illinois, Iowa, Kentucky, Maryland, Massachusetts, Michigan, Minnesota, Nevada, New Jersey, New Mexico, New York, North Dakota, Ohio, Oregon, Rhode Island, Vermont, Washington, and West Virginia.

*** Adults aged 50–64 years who have had a fecal occult blood test (FOBT) within the past year, or sigmoidoscopy within the past 5 years and an FOBT within the past 3 years, or colonoscopy within the past 10 years.

^†††^ Adults aged 18–64 years ever tested for HIV.

^§§§^ States that did not expand Medicaid as of January 1, 2014 (n = 20): Alabama, Alaska, Georgia, Idaho, Indiana, Louisiana, Maine, Mississippi, Missouri, Montana, Nebraska, New Hampshire, North Carolina, Oklahoma, Pennsylvania, South Carolina, Tennessee, Utah, Virginia, and Wisconsin.

The estimated prevalence of financial barriers to health care (i.e., unmet health care need because of cost, unmet prescribed medication need because of cost, and medical debt) were generally lower among working-aged adults residing in Medicaid expansion states than those residing in nonexpansion states regardless of primary source of health insurance coverage. For example, in Medicaid expansion states, the estimated prevalence of having medical bills that were being paid off over time was 24.5% among adults without a primary source of health insurance coverage, 20.2% among adults with public health plan coverage, and 19.8% among adults with private health insurance coverage; in nonexpansion states, the estimated prevalence was 31.0%, 24.5%, and 25.2%, respectively (Table 9).

The median estimated prevalence of adults who had a cost barrier to prescribed medication need was 9.1% and ranged from 5.6% in North Dakota to 13.8% in Mississippi. The median estimated prevalence of having medical debt (i.e., medical bills being paid off over time) was 24.0% and ranged from 11.3% in the District of Columbia to 34.2% in West Virginia. Crude and age-standardized state-specific estimates of financial barriers to health care are available (supplemental Table S5 https://stacks.cdc.gov/view/cdc/43255).

Overall and by state Medicaid expansion status, the estimated prevalence of breast, cervical, and colorectal cancer screening was typically lowest among working-aged adults without a primary source of health insurance coverage, followed by those who had public health plan coverage, and highest among those who had private health insurance coverage. For example, in Medicaid expansion states, the estimated prevalence of having a mammogram within the preceding 2 years was 48.5% among women without a primary source of health insurance coverage, 75.3% among women with public health plan coverage, and 81.9% among women with private health insurance coverage; in nonexpansion states, the estimated prevalence was 47.1%, 73.6%, and 80.8%, respectively. In contrast, the estimated prevalence of having ever been tested for HIV was highest among adults with public health care plans and lowest among those with private health insurance coverage. The estimated prevalence of influenza vaccination was lowest among adults with no primary source of insurance and generally similar for publically and privately insured adults. Similar estimates of these CPS by primary source of health insurance coverage were reported in Medicaid expansion and nonexpansion states (except for having ever been tested for HIV among adults without a primary source of coverage [42.7% versus 46.4%] and influenza vaccination among publically insured adults [35.6% versus 40.1%]) (Table 9).

#### Number of Health Care Visits in the Preceding 12 Months

Among adults aged 18–64 years, 15.6% had no health care visits during the preceding 12 months, 37.1% had 1–2 visits, and 47.3% had ≥3 visits. Adults without a primary source of health insurance coverage were more than three times as likely (37.0% versus 9.7% and 11.8%, respectively) to not have seen a health care professional during the preceding 12 months, compared with those who had public health plan coverage and private health insurance coverage, respectively (Table 10). Similar prevalence patterns by primary source of insurance coverage were found in expansion and nonexpansion states. The median estimated prevalence of adults who had not seen a health care professional during the preceding 12 months was 15.6% and ranged from 10.8% in Massachusetts to 24.7% in Nevada. The median estimated prevalence of adults who had 1–2 visits to a health care professional during the preceding 12 months was 37.4% and ranged from 33.4% in both Oregon and West Virginia to 43.3% in North Dakota. The median estimated prevalence of adults who had ≥3 visits to a health care professional during the preceding 12 months was 47.7% and ranged from 38.7% in Idaho to 52.0% in Massachusetts. Crude and age-standardized state-specific estimates of number of health care visits are available (supplemental Table S6 https://stacks.cdc.gov/view/cdc/43255).

**TABLE 10:** Crude and age-standardized* prevalence estimates of number of medical care visits during the preceding 12 months among adults aged 18–64 years with no primary source of health insurance coverage, public health plan coverage, and private health insurance coverage at the time of interview, by state Medicaid expansion status — Behavioral Risk Factor Surveillance System, 43 states,^†^ 2014

Category	n^§^	No primary source of health insurance coverage^¶^		Public health plan coverage**		Private health insurance coverage^††^	
		Crude	Age standardized	Crude	Age standardized	Crude	Age standardized
		%^§§^ (95% CI)	% (95% CI)	% (95% CI)	% (95% CI)	% (95% CI)	% (95% CI)
**Medicaid expansion states^¶¶^**	**121,366**						
None	14,735	39.0 (37.4–40.6)	38.8 (37.3–40.4)	10.3 ( 9.5–11.0)	10.5 ( 9.8–11.4)	11.3 (10.9–11.6)	11.8 (11.4–12.3)
1–2 visits	43,392	36.4 (34.8–37.9)	36.0 (34.5–37.6)	27.8 (26.8–28.9)	28.4 (27.3–29.6)	39.1 (38.5–39.7)	39.3 (38.7–39.9)
≥3 visits	63,239	24.6 (23.3–26.0)	25.1 (23.8–26.5)	61.9 (60.8–63.1)	61.0 (59.8–62.2)	49.7 (49.1–50.3)	48.9 (48.2–49.5)
Medicaid nonexpansion states***	**98,600**						
None	13,774	35.4 (34.1–36.8)	35.2 (33.9–36.5)	8.0 ( 7.3–8.7)	8.4 ( 7.6–9.2)	11.4 (11.0–11.8)	11.9 (11.4–12.3)
1–2 visits	36,981	37.4 (36.1–38.7)	37.2 (35.9–38.5)	25.9 (24.8–27.0)	26.9 (25.7–28.2)	40.4 (39.7–41.0)	40.6 (40.0–41.3)
≥3 visits	47,845	27.2 (26.0–28.4)	27.6 (26.4–28.7)	66.1 (64.9–67.3)	64.7 (63.3–66.0)	48.2 (47.6–48.8)	47.5 (46.8–48.2)
**Overall**	**219,966**						
None	28,509	37.2 (36.2–38.2)	37.0 (36.0–38.0)	9.4 ( 8.8–9.9)	9.7 ( 9.2–10.3)	11.3 (11.0–11.6)	11.8 (11.5–12.2)
1–2 visits	80,373	36.9 (35.9–37.9)	36.6 (35.6–37.6)	27.1 (26.3–27.9)	27.9 (27.0–28.7)	39.6 (39.2–40.1)	39.9 (39.4–40.3)
≥3 visits	111,084	25.9 (25.0–26.8)	26.4 (25.5–27.3)	63.6 (62.7–64.4)	62.4 (61.5–63.3)	49.1 (48.6–49.5)	48.3 (47.8–48.7)

**Abbreviations:** CI = confidence interval; n = sample size.

* Age standardized to the 2000 projected population for the United States.

^†^ Alabama, Alaska, Arizona, Colorado, Connecticut, Delaware, District of Columbia, Georgia, Idaho, Illinois, Indiana, Iowa, Kentucky, Louisiana, Maine, Maryland, Massachusetts, Michigan, Minnesota, Mississippi, Missouri, Montana, Nebraska, Nevada, New Hampshire, New Jersey, New Mexico, New York, North Carolina, North Dakota, Ohio, Oklahoma, Oregon, Pennsylvania, Rhode Island, South Carolina, Tennessee, Utah, Vermont, Virginia, Washington, West Virginia, and Wisconsin. Data from Missouri are available from the Missouri Department of Health and Senior Services.

^§^ Unweighted sample size.

^¶^ Includes persons who, when asked about their primary source of health care coverage, said they had none and persons who did not have any kind of health care coverage at the time of interview.

** Public health insurance includes Medicare; Medicaid or other state program; TRICARE (formerly CHAMPUS), Veterans Affairs, or military plan; Alaska Native, Indian Health Service, Tribal Health Services, or some other source.

^††^ Private health insurance includes a plan purchased through an employer or union (includes plans purchased through another person’s employer) or a plan that they or another family member purchased on their own.

^§§^ Weighted percentage.

^¶¶^ States that expanded Medicaid as of January 1, 2014 (n = 23): Arizona, Colorado, Connecticut, Delaware, District of Columbia, Illinois, Iowa, Kentucky, Maryland, Massachusetts, Michigan, Minnesota, Nevada, New Jersey, New Mexico, New York, North Dakota, Ohio, Oregon, Rhode Island, Vermont, Washington, and West Virginia.

*** States that did not expand Medicaid as of January 1, 2014 (n = 20): Alabama, Alaska, Georgia, Idaho, Indiana, Louisiana, Maine, Mississippi, Missouri, Montana, Nebraska, New Hampshire, North Carolina, Oklahoma, Pennsylvania, South Carolina, Tennessee, Utah, Virginia, and Wisconsin.

#### Satisfaction with Received Health Care

Among adults aged 18–64 years who had received health care during the preceding 12 months, 62.3% were very satisfied with the health care they received, 33.7% were somewhat satisfied, and 4.0% were not at all satisfied. The median estimated prevalence of adults who were very or somewhat satisfied was 96.1% and ranged from 92.7% in Nevada to 98.0% in Colorado. Crude and age-standardized state-specific estimates for satisfaction with received health care are available (supplemental Table S6 https://stacks.cdc.gov/view/cdc/43255). Among adults who had received health care during the preceding 12 months, those in expansion and nonexpansion states reported similar levels of satisfaction with received health care by primary source of health insurance coverage. For example, overall and in Medicaid expansion states and nonexpansion states, adults without a primary source of insurance were more than twice as likely to report that they were not at all satisfied with the health care they received, compared with those with public health care plans (overall: 11.4% versus 4.6%; Medicaid expansion states: 11.3% versus 4.5%; nonexpansion states: 11.5% versus 4.8%). In addition, adults without a primary source of insurance were about five times as likely to report that they were not at all satisfied with the health care they received, compared with those with private health insurance coverage (Table 11).

**TABLE 11:** Crude and age-standardized* prevalence estimates of satisfaction with received health care during the preceding 12 months among adults aged 18–64 years with no primary source of health insurance coverage, public health plan coverage, and private health insurance coverage at the time of interview, by state Medicaid expansion status — Behavioral Risk Factor Surveillance System, 43 states,^†^ 2014

Category	n^§^	No primary source of health insurance coverage^¶^		Public health plan coverage**		Private health insurance coverage^††^	
		Crude	Age standardized	Crude	Age standardized	Crude	Age standardized
		%^§§^ (95% CI)	% (95% CI)	% (95% CI)	% (95% CI)	% (95% CI)	% (95% CI)
**Medicaid expansion states^¶¶^**	**105,704**						
Very satisfied	70,024	46.0 (43.9–48.1)	46.9 (44.9–48.9)	58.7 (57.5–60.0)	58.3 (57.0–59.6)	67.1 (66.5–67.7)	66.1 (65.4–66.8)
Somewhat satisfied	32,424	42.5 (40.4–44.6)	41.8 (39.8–43.9)	36.8 (35.6–38.0)	37.2 (35.9–38.5)	30.6 (30.0–31.2)	31.6 (30.9–32.2)
Not at all satisfied	3,256	11.5 (10.2–13.0)	11.3 (10.0–12.7)	4.5 ( 4.0–5.0)	4.5 ( 4.0–5.1)	2.3 ( 2.1–2.5)	2.3 ( 2.1–2.5)
Medicaid nonexpansion states***	**83,969**						
Very satisfied	54,670	45.4 (43.7–47.1)	45.9 (44.3–47.6)	61.4 (60.1–62.6)	60.6 (59.2–62.0)	66.9 (66.2–67.5)	66.3 (65.6–67.0)
Somewhat satisfied	26,484	42.9 (41.2–44.6)	42.5 (40.9–44.2)	34.1 (32.9–35.4)	34.6 (33.3–36.0)	30.7 (30.1–31.3)	31.2 (30.5–31.9)
Not at all satisfied	2,815	11.7 (10.6–12.9)	11.5 (10.5–12.7)	4.5 ( 4.0–5.1)	4.8 ( 4.1–5.5)	2.4 ( 2.2–2.7)	2.5 ( 2.2–2.7)
**Overall**	**189,673**						
Very satisfied	124,694	45.7 (44.4–47.0)	46.4 (45.1–47.7)	59.8 (58.9–60.7)	59.2 (58.2–60.2)	67.0 (66.6–67.5)	66.2 (65.7–66.7)
Somewhat satisfied	58,908	42.7 (41.4–44.0)	42.2 (40.9–43.5)	35.7 (34.8–36.6)	36.2 (35.3–37.2)	30.6 (30.2–31.1)	31.4 (30.9–31.9)
Not at all satisfied	6,071	11.6 (10.8–12.6)	11.4 (10.6–12.3)	4.5 ( 4.1–4.9)	4.6 ( 4.2–5.0)	2.3 ( 2.2–2.5)	2.4 ( 2.2–2.6)

**Abbreviations:** CI = confidence interval; n = sample size.

* Age standardized to the 2000 projected population for the United States.

^†^ Alabama, Alaska, Arizona, Colorado, Connecticut, Delaware, District of Columbia, Georgia, Idaho, Illinois, Indiana, Iowa, Kentucky, Louisiana, Maine, Maryland, Massachusetts, Michigan, Minnesota, Mississippi, Missouri, Montana, Nebraska, Nevada, New Hampshire, New Jersey, New Mexico, New York, North Carolina, North Dakota, Ohio, Oklahoma, Oregon, Pennsylvania, Rhode Island, South Carolina, Tennessee, Utah, Vermont, Virginia, Washington, West Virginia, and Wisconsin. Data from Missouri are available from the Missouri Department of Health and Senior Services.

^§^ Unweighted sample size.

^¶^ Includes persons who, when asked about their primary source of health care coverage, said they had none and persons who did not have any kind of health care coverage at the time of interview.

** Public health insurance includes Medicare; Medicaid or other state program; TRICARE (formerly CHAMPUS), Veterans Affairs, or military plan; Alaska Native, Indian Health Service, Tribal Health Services, or some other source.

^††^ Private health insurance includes a plan purchased through an employer or union (includes plans purchased through another person’s employer) or a plan that they or another family member purchased on their own.

^§§^ Weighted percentage.

^¶¶^ States that expanded Medicaid as of January 1, 2014 (n = 23): Arizona, Colorado, Connecticut, Delaware, District of Columbia, Illinois, Iowa, Kentucky, Maryland, Massachusetts, Michigan, Minnesota, Nevada, New Jersey, New Mexico, New York, North Dakota, Ohio, Oregon, Rhode Island, Vermont, Washington, and West Virginia.

*** States that did not expand Medicaid as of January 1, 2014 (n = 20): Alabama, Alaska, Georgia, Idaho, Indiana, Louisiana, Maine, Mississippi, Missouri, Montana, Nebraska, New Hampshire, North Carolina, Oklahoma, Pennsylvania, South Carolina, Tennessee, Utah, Virginia, and Wisconsin.

### Continuity of Health Insurance Coverage

#### Overall and by State Medicaid Expansion Status, State, and FPL

In the 43 states using questions from the optional module, 75.6% of adults aged 18–64 years had continuous health insurance coverage, 12.9% had a gap in coverage, and 11.5% had been uninsured for >12 months. In Medicaid expansion states, 77.4% were continuously insured during the preceding 12 months, 13.0% experienced a gap in insurance, and 9.6% were uninsured during the preceding 12 months. In nonexpansion states, 73.4% were continuously insured during the preceding 12 months, 12.8% experienced a gap in insurance, and 13.9% were uninsured during the preceding 12 months (Table 12).

**TABLE 12:** Crude and age-standardized* prevalence estimates of continuous health insurance during 12 months before interview, with a gap in health insurance during 12 months before interview, or no health insurance for >12 months before interview among adults aged 18–64 years, by state Medicaid expansion status and federal poverty level — Behavioral Risk Factor Surveillance System, 43 states,^†^ 2014

Category	n^§^	Continuously insured during 12 mos before interview		Gap in insurance during 12 mos before interview		No health insurance for >12 mos before interview	
		Crude	Age standardized	Crude	Age standardized	Crude	Age standardized
		%^¶^ (95% CI)	% (95% CI)	% (95% CI)	% (95% CI)	% (95% CI)	% (95% CI)
Medicaid expansion states**	**125,265**	**78.5 (78.0–78.9)**	**77.4 (76.9–77.9)**	**12.5 (12.1–12.8)**	**13.0 (12.6–13.4)**	**9.1 (8.7–9.4)**	**9.6 (9.3–10.0)**
<100 of FPL	13,524	57.4 (55.9–58.9)	57.6 (56.1–59.1)	22.0 (20.8–23.2)	21.9 (20.7–23.1)	20.6 (19.3–21.9)	20.6 (19.3–21.9)
≥100%–≤400% of FPL	40,770	73.2 (72.4–74.1)	72.4 (71.5–73.3)	15.6 (15.0–16.3)	16.1 (15.4–16.8)	11.1 (10.5–11.8)	11.5 (10.8–12.2)
>400% of FPL	50,798	94.0 (93.6–94.4)	93.5 (92.9–94.0)	4.1 ( 3.8–4.5)	4.6 ( 4.2–5.0)	1.8 ( 1.6–2.1)	2.0 ( 1.7–2.3)
Unknown	20,173	75.3 (74.2–76.4)	74.4 (73.2–75.5)	14.8 (14.0–15.8)	15.1 (14.2–16.0)	9.9 ( 9.1–10.7)	10.5 ( 9.7–11.4)
**Medicaid nonexpansion states^††^**	**101,883**	**74.5 (74.0–75.0)**	**73.4 (72.9–73.9)**	**12.2 (11.8–12.6)**	**12.8 (12.4–13.2)**	**13.3 (12.9–13.7)**	**13.9 (13.5–14.3)**
<100 of FPL	13,180	48.5 (47.1–49.9)	48.6 (47.3–50.0)	20.7 (19.6–21.9)	20.5 (19.4–21.6)	30.8 (29.5–32.1)	30.9 (29.6–32.2)
≥100%–≤400% of FPL	39,417	71.3 (70.5–72.0)	70.4 (69.5–71.2)	14.5 (13.9–15.1)	15.0 (14.3–15.6)	14.2 (13.6–14.9)	14.6 (14.0–15.3)
>400% of FPL	36,650	93.0 (92.5–93.4)	91.9 (91.2–92.6)	4.8 ( 4.4–5.2)	5.6 ( 5.1–6.2)	2.3 ( 2.0–2.6)	2.5 ( 2.1–2.8)
Unknown	12,636	72.1 (70.7–73.5)	70.1 (68.6–71.6)	12.8 (11.8–13.8)	13.3 (12.2–14.4)	15.1 (14.0–16.3)	16.6 (15.4–18.0)
**Overall**	**227,148**	**76.7 (76.4–77.1)**	**75.6 (75.3–76.0)**	**12.3 (12.1–12.6)**	**12.9 (12.6–13.2)**	**10.9 (10.7–11.2)**	**11.5 (11.2–11.7)**
<100 of FPL	26,704	53.3 (52.3–54.3)	53.5 (52.4–54.5)	21.4 (20.6–22.3)	21.2 (20.4–22.1)	25.3 (24.4–26.2)	25.3 (24.4–26.2)
≥100%–≤400% of FPL	80,187	72.3 (71.7–72.9)	71.4 (70.8–72.1)	15.1 (14.6–15.6)	15.6 (15.1–16.1)	12.6 (12.2–13.1)	13.0 (12.5–13.5)
>400% of FPL	87,448	93.6 (93.3–93.9)	92.8 (92.4–93.2)	4.4 ( 4.1–4.7)	5.0 ( 4.7–5.3)	2.0 ( 1.8–2.2)	2.2 ( 1.9–2.4)
Unknown	32,809	74.1 (73.3–75.0)	72.9 (72.0–73.8)	14.1 (13.4–14.8)	14.4 (13.7–15.1)	11.8 (11.2–12.5)	12.7 (12.0–13.4)

**Abbreviations:** CI = confidence interval; FPL = federal poverty level; n = sample size.

* Age standardized to the 2000 projected population for the United States.

^†^ Alabama, Alaska, Arizona, Colorado, Connecticut, Delaware, District of Columbia, Georgia, Idaho, Illinois, Indiana, Iowa, Kentucky, Louisiana, Maine, Maryland, Massachusetts, Michigan, Minnesota, Mississippi, Missouri, Montana, Nebraska, Nevada, New Hampshire, New Jersey, New Mexico, New York, North Carolina, North Dakota, Ohio, Oklahoma, Oregon, Pennsylvania, Rhode Island, South Carolina, Tennessee, Utah, Vermont, Virginia, Washington, West Virginia, and Wisconsin. Data from Missouri are available from the Missouri Department of Health and Senior Services.

^§^ Unweighted sample size.

^¶^ Weighted percentage.

** States that expanded Medicaid as of January 1, 2014 (n = 23): Arizona, Colorado, Connecticut, Delaware, District of Columbia, Illinois, Iowa, Kentucky, Maryland, Massachusetts, Michigan, Minnesota, Nevada, New Jersey, New Mexico, New York, North Dakota, Ohio, Oregon, Rhode Island, Vermont, Washington, and West Virginia.

^††^ States that did not expand Medicaid as of January 1, 2014 (n = 20): Alabama, Alaska, Georgia, Idaho, Indiana, Louisiana, Maine, Mississippi, Missouri, Montana, Nebraska, New Hampshire, North Carolina, Oklahoma, Pennsylvania, South Carolina, Tennessee, Utah, Virginia, and Wisconsin.

For every income group, estimated prevalence of adults with continuous insurance in Medicaid expansion states was higher relative to nonexpansion states whereas, for two of the three income groups, estimated prevalence of uninsured adults in expansion states was lower compared with nonexpansion states. For example, among working-aged adults with household income <100% of FPL, 57.6% of those in Medicaid expansion states were continuously insured during the preceding 12 months, compared with 48.6% in nonexpansion states. Among adults with household income <100% of FPL and ≥100%–≤400% of FPL, 20.6% and 11.5%, respectively, of those residing in expansion states were uninsured for >12 months compared with 30.9% and 14.6%, respectively, in nonexpansion states. Among adults with household income >400% of FPL, similar estimates of no health insurance coverage during the preceding year were found in Medicaid expansion states and nonexpansion states (Table 12).

The median estimated prevalence of adults who had continuous health insurance coverage during the preceding 12 months was 75.9% and ranged from 65.6% in Georgia to 89.0% in Massachusetts. The median estimated prevalence of adults who had a gap in coverage was 13.0% and ranged from 8.7% in Massachusetts to 17.9% in Kentucky. The median estimated prevalence of adults who were uninsured during the preceding 12 months was 11.2% and ranged from 2.3% in Massachusetts to 20.0% in Georgia. Crude and age-standardized state-specific estimates for continuity of health insurance coverage are available (supplemental Table S7 https://stacks.cdc.gov/view/cdc/43255).

#### Health Care Access and CPS Use

Among adults aged 18–64 years, those who were continuously insured were twice as likely to have a usual source of health care (82.1% versus 37.6%) and to have had a routine checkup within the preceding 12 months (71.6% versus 34.2%), compared with those who had no health insurance for >12 months (Table 13). Working-aged adults with continuous health insurance were approximately one sixth as likely (7.7% versus 47.1%) to have unmet health care need because of cost and about one third as likely (6.5% versus 18.4%) to have unmet prescribed medication need because of cost, compared with those with no health insurance during the preceding 12 months. The estimated prevalence of having a cost barrier to prescribed medication need or medical bills being paid off over time was highest among adults with a gap in health insurance coverage during the preceding 12 months (22.9% and 34.1%, respectively), followed by those without health insurance for >12 months (18.4% and 27.1%, respectively), and was lowest among those with continuous insurance (6.5% and 20.4%, respectively). For these health care access measures, similar prevalence patterns by health insurance status during the preceding 12 months were found in Medicaid expansion states and nonexpansion states (Table 13).

**TABLE 13:** Crude and age-standardized* prevalence estimates of health care access and clinical preventive services use among adults aged 18–64 years with continuous health insurance during 12 months before interview, with a gap in health insurance during 12 months before interview, or no health insurance for >12 months before interview, by state Medicaid expansion status — Behavioral Risk Factor Surveillance System, 43 states,^†^ 2014

Category	n^§^	Continuously insured during 12 mos before interview		Gap in insurance during 12 mos before interview		No health insurance for >12 mos before interview	
		Crude	Age standardized	Crude	Age standardized	Crude	Age standardized
		%^¶^ (95% CI)	% (95% CI)	% (95% CI)	% (95% CI)	% (95% CI)	% (95% CI)
Medicaid expansion states**							
**Health care access**							
Usual source of health care	124,779	84.5 (84.1–85.0)	83.0 (82.5–83.5)	63.9 (62.4–65.4)	64.8 (63.3–66.2)	35.3 (33.5–37.2)	36.2 (34.4–38.0)
Routine checkup within preceding 12 mos	123,846	72.7 (72.2–73.2)	71.4 (70.8–71.9)	59.0 (57.5–60.5)	59.3 (57.8–60.7)	32.6 (30.8–34.5)	33.0 (31.2–34.9)
Cost barrier to health care need during preceding 12 mos	124,994	7.3 (7.0–7.6)	7.3 (7.0–7.7)	40.4 (38.9–41.9)	40.8 (39.3–42.2)	45.4 (43.4–47.3)	45.1 (43.2–47.0)
Cost barrier to prescribed medication need during preceding 12 mos	125,023	5.9 (5.6–6.2)	5.9 (5.6–6.2)	21.7 (20.5–22.9)	22.1 (21.0–23.3)	15.6 (14.3–17.1)	15.8 (14.5–17.3)
Medical bills being paid off over time during preceding 12 mos	124,291	18.1 (17.7–18.5)	18.3 (17.8–18.7)	31.2 (29.9–32.6)	31.4 (30.1–32.8)	23.4 (21.9–25.1)	23.5 (22.0–25.1)
**CPS**							
Mammogram within past 2 yrs (women aged 50–64 yrs)	35,542	81.9 (81.0–82.6)	81.8 (80.9–82.6)	64.7 (61.5–67.8)	64.7 (61.5–67.7)	38.2 (33.8–42.8)	38.2 (33.8–42.9)
Pap test within past 3 yrs (women aged 21–64 yrs who have not had a hysterectomy)	51,970	86.0 (85.4–86.7)	85.9 (85.2–86.6)	77.5 (75.6–79.4)	76.8 (74.9–78.6)	61.8 (58.5–65.1)	60.6 (57.4–63.7)
Colorectal cancer screening^††^ (adults aged 50–64 yrs)	58,808	66.7 (65.9–67.5)	65.9 (65.1–66.7)	44.9 (42.3–47.4)	44.8 (42.3–47.4)	24.7 (21.6–28.0)	24.9 (21.8–28.2)
HIV test^§§^ (adults aged 18–64 yrs)	112,951	39.4 (38.8–40.0)	41.6 (41.0–42.2)	50.9 (49.4–52.5)	50.9 (49.4–52.4)	41.7 (39.7–43.7)	40.4 (38.5–42.4)
Influenza vaccination within preceding 12 mos (adults aged 18–64 yrs)	118,125	38.7 (38.2–39.3)	37.5 (36.9–38.1)	26.1 (24.7–27.5)	26.3 (25.0–27.7)	14.4 (13.1–15.9)	14.7 (13.4–16.2)
**Medicaid nonexpansion states^¶¶^**							
**Health care access**							
Usual source of health care	101,488	82.6 (82.2–83.1)	80.8 (80.3–81.4)	63.3 (61.7–64.9)	65.2 (63.8–66.6)	38.1 (36.6–39.7)	38.9 (37.4–40.3)
Routine checkup within preceding 12 mos	100,686	73.3 (72.8–73.8)	71.8 (71.3–72.4)	59.2 (57.7–60.8)	59.9 (58.4–61.4)	34.8 (33.3–36.4)	35.2 (33.7–36.7)
Cost barrier to health care need during preceding12 mos	101,648	8.2 (7.9–8.6)	8.2 (7.9–8.6)	41.5 (39.9–43.1)	41.9 (40.4–43.4)	48.5 (47.0–50.1)	48.7 (47.2–50.2)
Cost barrier to prescribed medication need during preceding 12 mos	101,740	7.3 (7.0–7.6)	7.3 (6.9–7.6)	23.2 (21.9–24.5)	23.9 (22.7–25.3)	20.4 (19.2–21.6)	20.7 (19.5–21.9)
Medical bills being paid off over time during preceding 12 mos	101,146	23.2 (22.7–23.7)	23.4 (22.9–24.0)	37.3 (35.8–38.8)	37.7 (36.2–39.2)	30.2 (28.8–31.6)	30.3 (28.9–31.7)
**CPS**							
Mammogram within past 2 yrs (women aged 50–64 yrs)	27,479	80.6 (79.8–81.4)	80.5 (79.7–81.3)	61.0 (57.6–64.3)	61.0 (57.6–64.3)	40.8 (37.7–44.0)	40.7 (37.5–43.9)
Pap test within past 3 yrs (women aged 21–64 yrs who have not had a hysterectomy)	39,471	87.3 (86.6–87.9)	87.5 (86.8–88.1)	79.4 (77.3–81.4)	78.0 (76.0–79.9)	63.1 (60.7–65.4)	61.5 (59.2–63.7)
Colorectal cancer screening**^††^** (adults aged 50–64 yrs)	46,090	66.5 (65.7–67.3)	65.6 (64.8–66.4)	44.7 (42.0–47.4)	44.4 (41.8–47.1)	24.3 (22.1–26.6)	24.3 (22.2–26.6)
HIV test^§§^ (adults aged 18–64 yrs)	93,395	38.3 (37.7–38.9)	40.5 (39.9–41.2)	50.0 (48.3–51.6)	49.5 (47.9–51.0)	46.8 (45.1–48.4)	46.1 (44.5–47.7)
Influenza vaccination within preceding 12 mos (adults aged 18–64 yrs)	97,135	39.6 (39.0–40.2)	38.4 (37.8–39.0)	26.2 (24.8–27.6)	26.6 (25.2–28.0)	14.9 (13.8–16.1)	15.1 (14.0–16.2)
**Overall**							
**Health care access**							
Usual source of health care	226,267	83.7 (83.4–84.1)	82.1 (81.7–82.4)	63.7 (62.6–64.7)	64.9 (63.9–65.9)	36.8 (35.6–38.0)	37.6 (36.5–38.8)
Routine checkup within preceding 12 mos	224,532	72.9 (72.6–73.3)	71.6 (71.2–72.0)	59.1 (58.0–60.2)	59.5 (58.5–60.6)	33.8 (32.6–35.0)	34.2 (33.0–35.4)
Cost barrier to health care need during preceding 12 mos	226,642	7.7 (7.5–7.9)	7.7 (7.5–8.0)	40.9 (39.8–42.0)	41.3 (40.2–42.3)	47.0 (45.8–48.3)	47.1 (45.8–48.3)
Cost barrier to prescribed medication need during preceding12 mos	226,763	6.5 (6.3–6.7)	6.5 (6.2–6.7)	22.3 (21.4–23.2)	22.9 (22.0–23.8)	18.1 (17.2–19.1)	18.4 (17.5–19.3)
Medical bills being paid off over time during preceding 12 mos	225,437	20.2 (19.9–20.6)	20.4 (20.1–20.8)	33.8 (32.8–34.9)	34.1 (33.1–35.1)	27.0 (26.0–28.1)	27.1 (26.1–28.1)
**CPS**							
Mammogram within past 2 years (women aged 50–64 yrs)	63,021	81.3 (80.8–81.9)	81.2 (80.6–81.8)	63.2 (60.8–65.4)	63.1 (60.8–65.4)	39.7 (37.1–42.4)	39.7 (37.1–42.4)
Pap test within past 3 yrs (women aged 21–64 yrs who have not had a hysterectomy)	91,441	86.5 (86.1–87.0)	86.6 (86.1–87.1)	78.3 (76.9–79.7)	77.3 (76.0–78.6)	62.6 (60.6–64.5)	61.1 (59.2–62.9)
Colorectal cancer screening^††^ (adults aged 50–64 yrs)	104,898	66.6 (66.1–67.2)	65.8 (65.2–66.3)	44.8 (42.9–46.7)	44.7 (42.8–46.5)	24.5 (22.6–26.4)	24.6 (22.8–26.5)
HIV test^§§^ (adults aged 18–64 yrs)	206,346	38.9 (38.5–39.4)	41.2 (40.7–41.6)	50.5 (49.4–51.7)	50.3 (49.2–51.4)	44.4 (43.1–45.7)	43.5 (42.2–44.7)
Influenza vaccination within preceding 12 mos (adults aged 18–64 yrs)	215,260	39.1 (38.7–39.5)	37.9 (37.5–38.3)	26.1 (25.1–27.1)	26.5 (25.5–27.4)	14.7 (13.8–15.6)	14.9 (14.1–15.8)

**Abbreviations:** CI = confidence interval; CPS = clinical preventive services; HIV = human immunodeficiency virus; n = sample size; Pap = Papanicolaou.

* Age standardized to the 2000 projected population for the United States.

^†^ Alabama, Alaska, Arizona, Colorado, Connecticut, Delaware, District of Columbia, Georgia, Idaho, Illinois, Indiana, Iowa, Kentucky, Louisiana, Maine, Maryland, Massachusetts, Michigan, Minnesota, Mississippi, Missouri, Montana, Nebraska, Nevada, New Hampshire, New Jersey, New Mexico, New York, North Carolina, North Dakota, Ohio, Oklahoma, Oregon, Pennsylvania, Rhode Island, South Carolina, Tennessee, Utah, Vermont, Virginia, Washington, West Virginia, and Wisconsin. Data from Missouri are available from the Missouri Department of Health and Senior Services.

^§^ Unweighted sample size.

^¶^ Weighted percentage.

** States that expanded Medicaid as of January 1, 2014 (n = 23): Arizona, Colorado, Connecticut, Delaware, District of Columbia, Illinois, Iowa, Kentucky, Maryland, Massachusetts, Michigan, Minnesota, Nevada, New Jersey, New Mexico, New York, North Dakota, Ohio, Oregon, Rhode Island, Vermont, Washington, and West Virginia.

^††^ Adults aged 50–64 years who had a fecal occult blood test (FOBT) within the past year, or sigmoidoscopy within the past 5 years and an FOBT within the past 3 years, or colonoscopy within the past 10 years.

^§§^ Adults aged 18–64 years ever tested for HIV.

^¶¶^ States that did not expand Medicaid as of January 1, 2014 (n = 20): Alabama, Alaska, Georgia, Idaho, Indiana, Louisiana, Maine, Mississippi, Missouri, Montana, Nebraska, New Hampshire, North Carolina, Oklahoma, Pennsylvania, South Carolina, Tennessee, Utah, Virginia, and Wisconsin.

Among adults with continuous coverage, 83.0% of those in Medicaid expansion states had a usual source of health care, compared with 80.8% in nonexpansion states. Similarly, among adults with continuous coverage, 7.3% of those in expansion states had unmet health care need because of cost, 5.9% had unmet prescription need because of cost, and 18.3% had medical bills being paid off over time, compared with 8.2%, 7.3%, and 23.4%, respectively, in nonexpansion states. Among adults who were without health insurance for >12 months, 45.1% of those in expansion states had unmet health care need because of cost, 15.8% had unmet prescription need because of because of cost, and 23.5% had medical bills being paid off over time, compared with 48.7%, 20.7%, and 30.3%, respectively, in nonexpansion states. Among adults who had a gap in coverage during the preceding 12 months, 31.4% of those in expansion states had medical bills being paid off over time, compared with 37.7% in nonexpansion states (Table 13).

Overall and by state Medicaid expansion status, the estimated prevalence of breast, cervical, and colorectal cancer screening and influenza vaccination was highest among adults who were continuously insured, followed by those who had a gap in insurance coverage, and lowest among those who were without health insurance for >12 months, respectively. For example, in Medicaid expansion states, the estimated prevalence of colorectal cancer screening as recommended by USPSTF was 65.9% among adults with continuous coverage, 44.8% among those who had a gap in coverage, and 24.9% among those uninsured for >12 months; in nonexpansion states, these percentages were 65.6%, 44.4%, and 24.3%, respectively. The estimated prevalence of having ever been tested for HIV was highest among adults who had a gap in insurance coverage during the preceding 12 months, compared with those who were either continuously insured or uninsured during the preceding 12 months. Similar estimates of these CPS by continuity of health insurance coverage during the preceding 12 months were reported in Medicaid expansion and nonexpansion states (except for cervical cancer screening among continuously insured women [85.9% versus 87.5%] and having ever been tested for HIV among uninsured adults [40.4% versus 46.1%]) (Table 13).

#### Number of Health Care Visits During the Preceding 12 Months

Among adults aged 18–64 years, those who were without health insurance for >12 months were four times as likely (43.5% versus 10.8%) to not have seen a health care professional during the preceding 12 months, compared with those who were continuously insured. Among adults with no health insurance during the preceding 12 months, the estimated prevalence of not having seen a health care professional during the preceding 12 months was higher in Medicaid expansion states (47.0%) than nonexpansion states (40.4%). The estimated prevalence of health care visits (i.e., none, 1–2, or ≥3) among adults who were continuously insured or who had a gap in coverage during the preceding 12 months was similar by state Medicaid expansion status (Table 14).

**TABLE 14:** Crude and age-standardized* prevalence estimates of number of medical care visits during preceding 12 months among adults aged 18–64 years with continuous health insurance during 12 months before interview, with a gap in health insurance during 12 months before interview, or no health insurance for >12 months before interview, by state Medicaid expansion status — Behavioral Risk Factor Surveillance System, 43 states,^†^ 2014

Category	n^§^	Continuously insured during 12 mos before interview		Gap in insurance during 12 mos before interview		No health insurance for >12 mos before interview	
		Crude	Age standardized	Crude	Age standardized	Crude	Age standardized
		%^¶^ (95% CI)	% (95% CI)	% (95% CI)	% (95% CI)	% (95% CI)	% (95% CI)
Medicaid expansion states**	**122,126**						
None	14,791	10.3 ( 9.9–10.6)	10.8 (10.4–11.2)	19.0 (17.8–20.3)	19.0 (17.8–20.2)	47.1 (45.2–49.1)	47.0 (45.0–48.9)
1–2 visits	43,642	36.7 (36.2–37.3)	37.2 (36.6–37.8)	35.2 (33.7–36.7)	35.0 (33.6–36.4)	34.8 (33.0–36.8)	34.7 (32.9–36.6)
≥3 visits	63,693	53.0 (52.4–53.6)	52.0 (51.4–52.6)	45.8 (44.3–47.3)	46.1 (44.6–47.6)	18.0 (16.6–19.5)	18.4 (17.0–19.8)
**Medicaid nonexpansion states^††^**	**99,381**						
None	13,838	10.2 ( 9.8–10.5)	10.7 (10.3–11.1)	17.3 (16.1–18.5)	17.3 (16.1–18.5)	40.7 (39.1–42.3)	40.4 (38.9–42.0)
1–2 visits	37,280	37.7 (37.1–38.3)	38.2 (37.6–38.8)	37.9 (36.3–39.5)	37.4 (35.9–39.0)	35.6 (34.1–37.1)	35.5 (34.0–37.0)
≥3 visits	48,263	52.2 (51.6–52.7)	51.1 (50.4–51.7)	44.8 (43.2–46.4)	45.3 (43.8–46.8)	23.7 (22.5–25.1)	24.1 (22.8–25.4)
**Overall**	**221,507**						
None	28,629	10.2 (10.0–10.5)	10.8 (10.5–11.1)	18.3 (17.4–19.2)	18.2 (17.4–19.1)	43.7 (42.5–45.0)	43.5 (42.2–44.7)
1–2 visits	80,922	37.1 (36.7–37.5)	37.6 (37.2–38.0)	36.3 (35.3–37.4)	36.0 (35.0–37.1)	35.2 (34.0–36.4)	35.1 (34.0–36.3)
≥3 visits	111,956	52.6 (52.2–53.1)	51.6 (51.2–52.1)	45.4 (44.3–46.5)	45.7 (44.6–46.8)	21.0 (20.1–22.0)	21.4 (20.5–22.4)

**Abbreviations:** CI = confidence interval; n = sample size.

* Age standardized to the 2000 projected population for the United States.

^†^ Alabama, Alaska, Arizona, Colorado, Connecticut, Delaware, District of Columbia, Georgia, Idaho, Illinois, Indiana, Iowa, Kentucky, Louisiana, Maine, Maryland, Massachusetts, Michigan, Minnesota, Mississippi, Missouri, Montana, Nebraska, Nevada, New Hampshire, New Jersey, New Mexico, New York, North Carolina, North Dakota, Ohio, Oklahoma, Oregon, Pennsylvania, Rhode Island, South Carolina, Tennessee, Utah, Vermont, Virginia, Washington, West Virginia, and Wisconsin. Data from Missouri are available from the Missouri Department of Health and Senior Services.

^§^ Unweighted sample size.

^¶^ Weighted percentage.

** States that expanded Medicaid as of January 1, 2014 (n = 23): Arizona, Colorado, Connecticut, Delaware, District of Columbia, Illinois, Iowa, Kentucky, Maryland, Massachusetts, Michigan, Minnesota, Nevada, New Jersey, New Mexico, New York, North Dakota, Ohio, Oregon, Rhode Island, Vermont, Washington, and West Virginia.

^††^ States that did not expand Medicaid as of January 1, 2014 (n = 20): Alabama, Alaska, Georgia, Idaho, Indiana, Louisiana, Maine, Mississippi, Missouri, Montana, Nebraska, New Hampshire, North Carolina, Oklahoma, Pennsylvania, South Carolina, Tennessee, Utah, Virginia, and Wisconsin.

#### Satisfaction with Received Health Care

Among adults aged 18–64 years, similar levels of satisfaction with received health care by continuity of health insurance coverage during the preceding 12 months were reported in Medicaid expansion and nonexpansion states. For example, overall and by state Medicaid expansion status, adults without health insurance coverage for >12 months were approximately five times as likely to report that they were not at all satisfied with the health care they received, compared with those who were continuously insured (overall: 13.1% versus 2.5%; Medicaid expansion states: 12.6% versus 2.4%; nonexpansion states: 13.4% versus 2.6%) (Table 15).

**TABLE 15:** Crude and age-standardized* prevalence estimates of satisfaction with received health care among adults aged 18–64 years with continuous health insurance during 12 months before interview, with a gap in health insurance during 12 months before interview, or no health insurance for >12 months before interview, by state Medicaid expansion status — Behavioral Risk Factor Surveillance System, 43 states,^†^ 2014

Category	n^§^	Continuously insured during 12 mos before interview		Gap in insurance during 12 mos before interview		No health insurance for >12 mos before interview	
		Crude	Age standardized	Crude	Age standardized	Crude	Age standardized
		%^¶^ (95% CI)	% (95% CI)	% (95% CI)	% (95% CI)	% (95% CI)	% (95% CI)
Medicaid expansion states**	**106,389**						
Very satisfied	70,469	66.8 (66.3–67.4)	66.0 (65.3–66.6)	46.8 (45.1–48.5)	47.1 (45.4–48.8)	45.1 (42.4–47.8)	46.2 (43.6–48.8)
Somewhat satisfied	32,649	30.7 (30.2–31.3)	31.6 (31.0–32.2)	45.3 (43.6–47.1)	45.0 (43.3–46.6)	42.0 (39.3–44.7)	41.2 (38.7–43.9)
Not at all satisfied	3,271	2.4 ( 2.3–2.6)	2.4 ( 2.3–2.6)	7.8 ( 7.0–8.8)	7.9 ( 7.0–8.9)	12.9 (11.2–14.9)	12.6 (10.9–14.5)
**Medicaid nonexpansion states^††^**	**84,673**						
Very satisfied	55,103	67.2 (66.6–67.8)	66.6 (66.0–67.3)	49.4 (47.6–51.2)	49.8 (48.0–51.5)	43.9 (41.9–45.9)	44.0 (42.0–46.1)
Somewhat satisfied	26,741	30.3 (29.7–30.8)	30.8 (30.1–31.4)	43.8 (42.0–45.6)	43.4 (41.7–45.1)	42.6 (40.6–44.7)	42.5 (40.6–44.6)
Not at all satisfied	2,829	2.5 ( 2.3–2.8)	2.6 ( 2.3–2.8)	6.8 ( 6.0–7.8)	6.8 ( 6.0–7.8)	13.5 (12.1–15.0)	13.4 (12.0–14.9)
**Overall**	**191,062**						
Very satisfied	125,572	67.0 (66.6–67.4)	66.2 (65.8–66.7)	47.9 (46.7–49.2)	48.3 (47.1–49.5)	44.4 (42.8–46.1)	44.9 (43.3–46.5)
Somewhat satisfied	59,390	30.5 (30.1–30.9)	31.3 (30.8–31.7)	44.7 (43.4–45.9)	44.3 (43.1–45.5)	42.3 (40.7–44.0)	42.0 (40.4–43.7)
Not at all satisfied	6,100	2.5 ( 2.3–2.6)	2.5 ( 2.4–2.7)	7.4 ( 6.8–8.1)	7.5 ( 6.8–8.1)	13.2 (12.1–14.5)	13.1 (12.0–14.3)

**Abbreviations:** CI = confidence interval; n = sample size.

* Age standardized to the 2000 projected population for the United States.

^†^ Alabama, Alaska, Arizona, Colorado, Connecticut, Delaware, District of Columbia, Georgia, Idaho, Illinois, Indiana, Iowa, Kentucky, Louisiana, Maine, Maryland, Massachusetts, Michigan, Minnesota, Mississippi, Missouri, Montana, Nebraska, Nevada, New Hampshire, New Jersey, New Mexico, New York, North Carolina, North Dakota, Ohio, Oklahoma, Oregon, Pennsylvania, Rhode Island, South Carolina, Tennessee, Utah, Vermont, Virginia, Washington, West Virginia, and Wisconsin. Data from Missouri are available from the Missouri Department of Health and Senior Services.

^§^ Unweighted sample size.

^¶^ Weighted percentage.

** States that expanded Medicaid as of January 1, 2014 (n = 23): Arizona, Colorado, Connecticut, Delaware, District of Columbia, Illinois, Iowa, Kentucky, Maryland, Massachusetts, Michigan, Minnesota, Nevada, New Jersey, New Mexico, New York, North Dakota, Ohio, Oregon, Rhode Island, Vermont, Washington, and West Virginia.

^††^ States that did not expand Medicaid as of January 1, 2014 (n = 20): Alabama, Alaska, Georgia, Idaho, Indiana, Louisiana, Maine, Mississippi, Missouri, Montana, Nebraska, New Hampshire, North Carolina, Oklahoma, Pennsylvania, South Carolina, Tennessee, Utah, Virginia, and Wisconsin.

## Discussion

Certain policy changes related to the ACA took effect on or before January 1, 2014, including required coverage without cost sharing of certain CPS, the option for states to broaden Medicaid eligibility and expand coverage, and tax credits to help make insurance coverage affordable. Post-2014 changes in health care access, such as source of health insurance coverage, attainment and continuity of coverage, financial barriers, preventive care services, and health outcomes, can be assessed and monitored with these 2014 state-specific data as baseline information.

The findings in this report indicate many disparities in health care access in 2014, including disparities between states that expanded Medicaid coverage and those states that did not expand it. In nonexpansion states, legal residents with household income between 100% and 400% of FPL could receive a tax credit to offset the cost of insurance premiums, but no new financial assistance was provided for those with household income below 100% of FPL (*58*). However, in Medicaid expansion states, legal residents with household income between 0% and 400% of FPL became eligible for affordable coverage options through tax credits and expanded Medicaid eligibility. Findings presented in this report show that prevalence estimates for many indicators of health care access, such as having health care coverage, having a usual source of care, having a routine checkup within the preceding 12 months, and not experiencing a cost barrier to health care need during the preceding 12 months, were higher among adults living below the poverty level in states that expanded Medicaid than in states that did not. Similarly, adults living below the poverty level in states that expanded Medicaid had higher prevalence estimates of breast and cervical cancer screening and influenza vaccination than those in states that did not expand Medicaid. These disparities could be partly explained by factors other than differences in eligibility for Medicaid because persons with income above the poverty level living in states that expanded Medicaid had higher rates of insurance coverage, having a usual source of health care, and not experiencing a cost barrier to health care need during the preceding 12 months than persons of comparable income living in states that did not expand Medicaid. Differences between the Medicaid expansion states and nonexpansion states in demographics, social, cultural, and economic factors, as well as state-level policy decisions, might also have a role in health care access and use among residents. However, the adjustment was made for differences in age, sex, race/ethnicity, and FPL and did not substantially change the results presented in this report. Further research is needed to clarify the effects of policy and investigate the effects of these and other factors such as health status, urbanicity (i.e., conditions that are unique to urban areas or more prevalent than in nonurban areas), and provider density on these disparities.

Researchers have found changes in health care access and use following expansion in Medicaid eligibility (*16*–*18*,*20*,*23*,*24*,*27*). In 2008, Oregon expanded Medicaid to persons selected by a lottery. Persons who were selected to receive Medicaid had an increased probability of having a usual source of health care, having a usual doctor, receiving CPS, and having improved self-reported mental and physical health compared with those who were not selected (*18*,*20*,*23*). Similarly, those selected for Medicaid also experienced reduced financial strain overall and a decreased probability of having financial trouble because of medical expenses (*18*,*20*,*23*). Another study found that mortality rates decreased in Maine, Arizona, and New York following expansions of Medicaid in those states, when compared with neighboring states (*16*). In future years it might be possible to use the BRFSS Health Care Access module to conduct similar studies to examine the effects of ACA on health care access and CPS use over time.

## Limitations

The findings in this report are subject to at least five limitations. First, the BRFSS survey design excludes persons living in institutions, nursing homes, long-term care facilities, military installations, and correctional institutions. In addition, persons who do not have either a landline number or cellular telephone are excluded from the survey. The optional module results might not be generalizable to the entire U.S. population aged 18–64 years, or subpopulations within this age group, overall or by state Medicaid expansion status because not all states fielded the module in 2014 (Arkansas, California, Florida, Hawaii, Kansas, South Dakota, Texas, and Wyoming did not). Second, BRFSS data are self-reported, so the information is subject to recall (e.g., time since last mammogram and type of colorectal cancer screening received) and social desirability bias (e.g., underreporting of HIV test and medical debt). Third, FPL was not calculated for persons who did not respond to the income question (~13%) or the number of children in the household question (<1%) or who had >14 adults in the household or were missing these data (<2%). In addition, the BRFSS question used to assess household income asks respondents to indicate whether their household income falls within specific income ranges; therefore, the mean of each range was used to estimate FPL and, for the top-end income level (i.e., ≥$75,000), the weighted mean of income from census data was used. Fourth, although BRFSS surveys are conducted in several languages other than English (i.e., Spanish, Mandarin, and Portuguese), the survey does not cover persons who speak other languages exclusively. Finally, although the measures examined in this report are reliable indicators of access to health care (e.g., insurance, usual health care provider, routine checkup, use of preventive health services, number of visits to a health care professional, and satisfaction with received health care), they also might be associated with other factors such as age, health status, and income. For example, persons with chronic conditions, whose health care need is ongoing, are more likely than those without chronic conditions to have health insurance coverage (*33*). Conversely, young adults and persons in good health might be less likely to have insurance coverage or to use health care services. Nevertheless, an examination of the effects of extending dependent coverage to young adults (aged 18–25 years) during 2009–2011 found that removal of one access barrier, no insurance coverage, accounted for almost all of the significant increases in past-year routine examination (from 44.1% to 47.8%) and past-year blood pressure screening (from 65.2% to 68.3%) and part of the significant increases in cholesterol screening (from 24.3% to 29.1%) and annual dental visit (55.2% to 60.9%) (*59*). Type of insurance coverage, continuity of coverage, or both also might be associated with other factors, such as health status, income level, and employment status (*19*,*31*,*33*,*34*,*37*). For example, poor health status or low income might increase the likelihood of having public health insurance because Medicaid eligibility includes having a severe disability, low income, or both (*33*).

## Conclusion

BRFSS is a cost-effective, timely, and flexible survey that makes data available to a broad range of audiences, including state health departments and policymakers, so they can assess and monitor the adverse health behaviors, chronic conditions, access to health care, use of CPS and other health care, and disabilities of state populations. The prevalence and trends from the BRFSS survey might differ from other national, state, and nongovernmental surveys because of differences in the survey instrument (e.g., wording of questions, number of questions focusing on a measure or topic, length of questionnaire, format of the questionnaire), mode of data collection (telephone, in person, online), and sampling frame. For example, unlike other federal surveys, BRFSS estimates of health insurance coverage include IHS coverage. In 2011, the core BRFSS question used to assess current health insurance coverage since 1993 was modified to include IHS as an example of health insurance. BRFSS 2014 data can be used to estimate the state-specific proportion of working-aged adults with IHS as their primary source of coverage in states that used the optional Health Care Access module.

One of the goals of BRFSS is to provide state-level data on health care access and use of CPS that can be used by state health departments, health care organizations, and policymakers. This report highlights findings from the 2014 BRFSS on health care access and use of preventive health services during the first year that many of the main provisions of the ACA were implemented. Adults who had been uninsured or who had inadequate coverage might experience improved access to health care, preventive health services and other health care, and, as a result, better health outcomes. Changes in health care access, CPS use, and health that might occur after 2014 can be assessed and monitored using BRFSS state-specific estimates from 2014.

## Footnotes

* Missouri included these questions in the state-added section of its survey; these data are available from the Missouri Department of Health and Senior Services.

† Michigan implemented Medicaid expansion on April 1, 2014, and is categorized as a Medicaid expansion state because the expansion was in effect for the majority of the year.

§ New Hampshire implemented Medicaid expansion on August 15, 2014, and is categorized as a Medicaid nonexpansion state because the expansion was not in effect for the majority of the year.
